# Tumor‐Derived CDC37 Inhibits Antigen Cross‐Presentation in Dendritic Cells and Impairs Anti‐Tumor Immunity in Breast Cancer

**DOI:** 10.1002/advs.202506518

**Published:** 2025-11-10

**Authors:** Ruxin Wang, Yunyi Zhang, Xiangyu Meng, Jianli Zhao, Yue Xing, Qian Ouyang, Ning Zhang, Huiping Chen, Nanyan Miao, Erwei Song, Di Huang

**Affiliations:** ^1^ Guangdong Provincial Key Laboratory of Malignant Tumor Epigenetics and Gene Regulation Guangdong‐Hong Kong Joint Laboratory for RNA Medicine Breast Tumor Center Sun Yat‐Sen Memorial Hospital Sun Yat‐Sen University Guangzhou 510120 China; ^2^ State Key Laboratory of Oncology in South China Sun Yat‐Sen University Guangzhou 510120 China; ^3^ Zenith Institute of Medical Sciences Guangzhou 510535 China; ^4^ State Key Laboratory of Proteomics Beijing 102206 China

**Keywords:** antigen cross‐presentation, CDC37, extracellular vesicles, immune checkpoint blockades, type 1 conventional dendritic cell

## Abstract

Tumor mutational burden (TMB), usually representing high immunogenicity, cannot always predict treatment response of immune checkpoint blockade (ICB). Here, it is showed that defective antigen cross‐presentation in type 1 conventional dendritic cells (cDC1) is responsible for lacking tumor‐specific cytotoxic T lymphocytes (CTLs) in triple‐negative breast cancer (TNBC) patients. Mechanistically, tumor cytosolic CDC37, shuttled via extracellular vesicles (EVs) into the endosomes of intratumor dendritic cells (DCs), inhibits antigen cross‐presentation by locking antigen binding to HSP90 and precluding their translocation from endosomes to cytoplasm. CDC37 knockdown in tumor cells or inhibiting CDC37/HSP90 interaction in DCs efficiently promotes antigen translocation and enhances their cross‐presentation, which improves ICB therapeutic responses. Clinically, high tumor CDC37 expression is associated with low infiltration of antigen‐specific CTLs and poor ICB efficacy in TNBC patients. Therefore, tumor EV‐shuttled CDC37 locks antigen/chaperone interaction and impairs antigen cross‐presentation in DCs. Moreover, targeting CDC37 is promising to enhance anti‐tumor immunity and reverse ICB resistance.

## Graphical abstract

Extracellular vesicle (EV)‐packaged CDC37 are released from TMB^hi^CTL^lo^ breast cancer cells with high CDC37 expression, and internalized into endo/phagosomes of dendritic cells (DCs). Within these compartments, CDC37 locked HSP90–antigen complex, preventing antigen release into the cytosol. Consequently, antigens are not processed by cytosolic proteasomes and fail to be presented on MHC class I molecules at the DC surface.

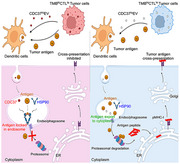

## 1 Introduction

Effective anti‐tumor immune responses depend heavily on efficient priming of tumor antigen specific cytotoxic CD8^+^ T lymphocytes (CTLs).^[^ ^1^ ^]^ In the past decade, immunotherapies that employ immune checkpoint blockade (ICB) to reactivate anti‐tumor immunity have achieved remarkable success in immune “hot” tumors with sufficient CTL infiltration, such as melanomas,^[^ ^2^ ^]^ non‐small cell lung carcinomas (NSCLC)^[^ ^3^ ^]^ and bladder carcinomas.^[^ ^4^ ^]^ However, therapeutic responses of immune “cold” tumors that lack CTL infiltration, including breast cancers, to ICB remain poor and the effects do not last long.^[^ ^5^, ^6^ ^]^ It was suggested that low tumor mutational burden (TMB) with low immunogenicity of tumor cells is responsible for the poor immunotherapeutic responses of immune “cold” tumors,^[^ ^6^, ^7^, ^8^, ^9^, ^10^, ^11^, ^12^ ^]^ as tumors with low TMB bear less neoantigens and thus are incapable of priming tumor antigen‐specific CTLs, which are ICB responders.^[^ ^13^ ^]^ Nevertheless, high TMB is not always associated with high lymphocyte infiltration in breast cancer^[^ ^14^ ^]^ and surprisingly, some cancer patients with high TMB but low lymphocyte infiltration do not respond well to ICB.^[^ ^10^, ^14^ ^]^ Therefore, other factors, especially capability of antigen‐presenting cells (APCs) to cross‐present tumor antigens to prime CD8^+^ T lymphocytes, may determine the anti‐tumor immune responses as well as ICB therapeutic efficacy in cancer patients.

Cross‐presentation of tumor antigens is mainly carried out by type 1 conventional dendritic cells (cDC1),^[^ ^15^ ^]^ which were derived from bone marrows and differentiated to functional dendritic cells (DCs) in the tumors.^[^ ^16^, ^17^ ^]^ Efficient antigen cross‐presentation depends on sufficient cDC1 infiltration and their capability to capture, process and cross‐present antigens onto MHC, which will then prime naive CD8^+^ T cells.^[^ ^18^ ^]^ In mouse models, cDC1 deficiency induced by BATF3 knockout^[^ ^19^ ^]^ or tumor‐mediated immune inhibition via β‐catenin^[^ ^20^ ^]^ led to tumor immune evasion and resistance to anti‐PD‐1 therapies. In addition, lack of DC infiltration,^[^ ^21^ ^]^ increased apoptosis,^[^ ^22^ ^]^ and decreased maturation of the tumor‐infiltrating DCs^[^ ^23^ ^]^ in mouse tumors impaired T‐cell mediated anti‐tumor immunity. Further, conditional knockout of Sec 22b gene, a helper of protein transfer from endoplasmic reticulum to endosome or phagosome (endo/phagosome), in mouse DCs diminished antigen cross‐presentation, leading to defective CTL priming and resistance to anti‐PD‐1 treatment.^[^ ^24^ ^]^ In human, cDC1 abundance was associated with CD8^+^ T cell infiltration and better immunotherapeutic effects in melanoma patients.^[^ ^21^, ^25^, ^26^, ^27^ ^]^ Therefore, strategies that aim to enhance the abundance and function of tumor‐infiltrating cDC1 (TiDC) could potentially improve the responsiveness of cancer patients to immunotherapy.^[^ ^28^ ^]^ Here, we aimed to investigate the roles of cDC1 in immunotherapies of breast cancer patients, and to explore whether enhancing antigen cross‐presentation in cDC1 can be a potential strategy to improve ICB therapeutic efficacy.

## 2 Results

### 2.1 Antigen Cross‐Presenting was Inhibited in Breast Cancers with High TMB and Poor CTL Infiltration

**Figure 1:**
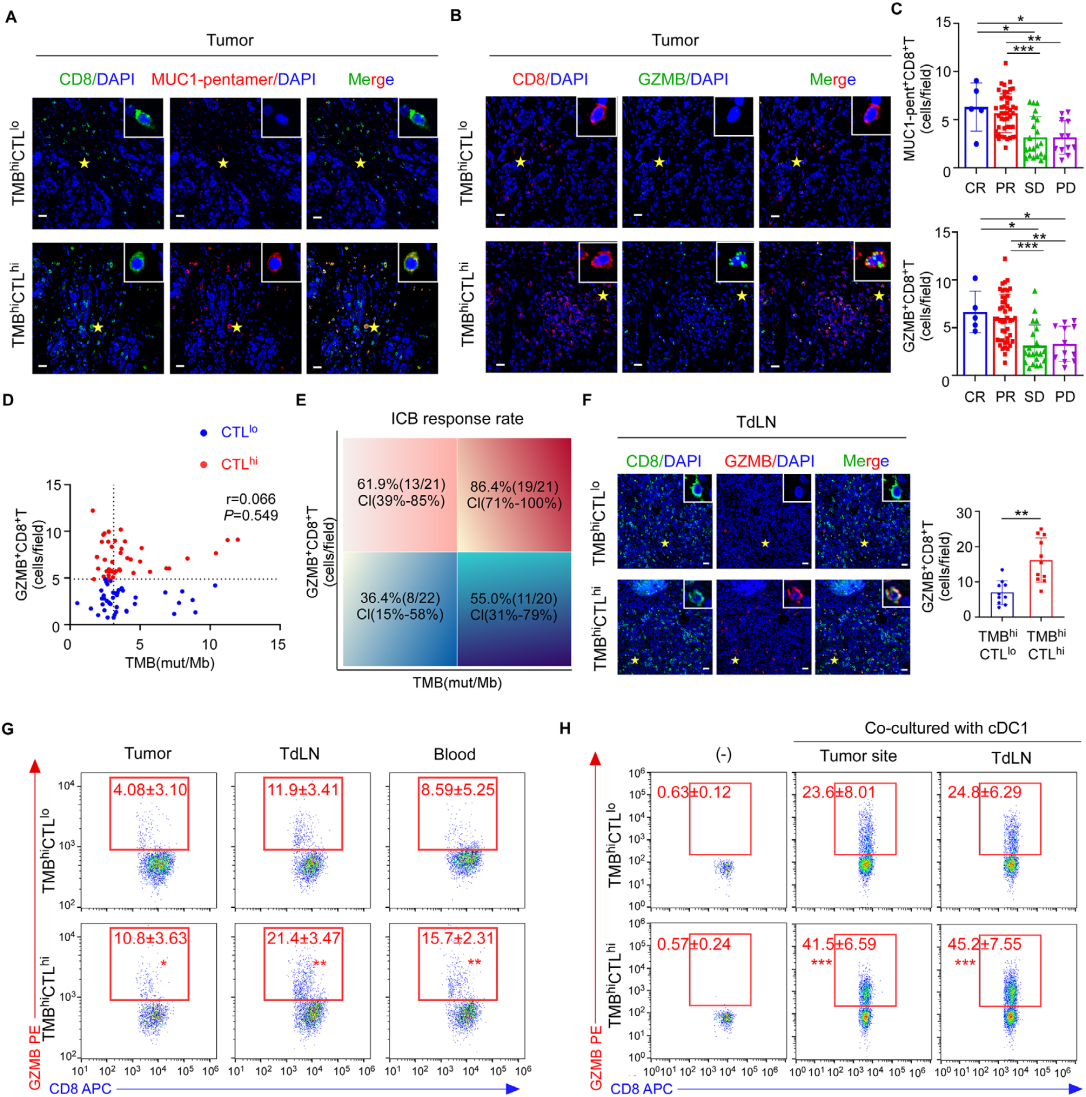
Antigen cross‐presenting was inhibited in breast cancers with high TMB and poor CTL infiltration. A,B). Representative immunofluorescence images of tumor‐specific CD8^+^ T cells in tumor site denoted by co‐staining of CD8^+^ and MUC1‐pentamer^+^ (A) or GZMB^+^ (B) in TNBC patients with high TMB and high CTL infiltration (TMB^hi^CTL^hi^, *n* = 21) or the ones with high TMB and low CTL infiltration (TMB^hi^CTL^lo^, *n* = 20). Asterisks denote the area of higher magnification images shown at the top right corner. Scale bar, 50 µm. C) The count of CD8^+^MUC1‐pentamer^+^ T cells (top) and CD8^+^GZMB^+^ T cells (bottom) in the tumor site of TNBC patients with different therapeutic responses to ICB. CR, complete response, *n* = 5; PR, partial response, *n* = 46; SD, stable disease, *n* = 21; PD, progressive disease, *n* = 12. D) Correlation between TMB and CD8^+^GZMB^+^ T cells in tumor biopsies of TNBC patients (*n* = 84. Spearman's correlation coefficient r and two‐tailed *P* value). Cutoffs of median of CD8^+^GZMB^+^ T cells and median of TMB are given by dashed vertical and horizontal lines, respectively. E) Response (PR and CR) rates in percentages and 95% confidence intervals (CI) in subgroups defined by the cutoffs given as dashed lines in (D). F) Representative images and quantification of CD8^+^GZMB^+^ T cells in TdLN of TNBC patients with TMB^hi^CTL^hi^ (*n* = 21) or TMB^hi^CTL^lo^ tumor (*n* = 20). Asterisks denote the area of higher magnification images shown at the top right corner. Scale bar, 50 µm. G) Representative flow cytometric plots and quantification of GZMB staining in the CD8^+^ T cells from tumor site, TdLN and blood of TMB^hi^CTL^hi^ (*n* = 9) and TMB^hi^CTL^lo^ (*n* = 6) TNBC patients. H) Naive CD8^+^ T cells were cultured alone (‐) or primed by cDC1 isolated from tumor site or TdLN of TMB^hi^CTL^hi^ (*n* = 9) or TMB^hi^CTL^lo^ (*n* = 6) TNBC patients. Representative flow cytometric plots and quantification of percentages of GZMB staining in the in vitro primed CD8^+^ T cells. Results are mean ± s.d. of independent experiments producing similar results (C, F–H). *P < 0.05, **P < 0.01, ***P < 0.001, compared with indicated group, were calculated using two‐tailed one‐way analysis of variance (ANOVA) with Tukey's multiple‐comparisons test (C) or compared with TMB^hi^CTL^lo^ group using two‐tailed Student's t test (F–H).

It remains controversial whether TMB of cancer patients could help to predict clinical outcomes of ICB therapy.^[^ ^13^, ^29^ ^]^ To evaluate the correlation of TMB and ICB treatment effects in breast cancer, we measured TMB and tumor‐specific CTL infiltration in the primary tumors of 84 triple‐negative breast cancer (TNBC) patients before ICB treatment and correlated their treatment responses with TMB as well as tumor antigen‐specific CTL infiltration. We observed that the infiltration of tumor‐specific CTLs, denoted by HLA‐A2‐restricted MUC1‐pentamer and CD8 double staining (**Figure** **1**A) or granzyme B (GZMB) and CD8 double staining (Figure 1B), was positively associated with ICB response (Figure 1C). Moreover, TMB, obtained from whole‐exome sequencing (WES), was significantly higher in the patients with complete response (CR) or partial response (PR) than those with disease progression (PD) and stable disease (SD) (Figure S1A, Supporting Information). In a multivariable analysis, either TMB or tumor‐specific CTL infiltration could independently predict ICB responses (Table S1, Supporting Information). However, we found no significant association of the tumor‐specific CTL (GZMB^+^CD8^+^ T cells) infiltration with TMB values (Spearman's r = 0.066, *P *= 0.549) (Figure 1D). Interestingly, upon stratifying the patients in groups based on the median of TMB (3.08 mut/Mb) as previously reported,^[^ ^14^, ^30^ ^]^ as well as the median number of tumor‐specific CTLs (4.86 cells/ field), we observed an ICB response rate of 86.4% in the patients with high TMB and high CTL infiltration (TMB^hi^CTL^hi^) in contrast to only 36.4% and 55.0% in the patients with low TMB and low CTL infiltration (TMB^lo^CTL^lo^) and the patients with high TMB and low CTL infiltration (TMB^hi^CTL^lo^), respectively (Figure 1E).

To further explore the regulatory mechanisms underlying low CTL infiltration in the tumors with high TMB which might have more neoantigens to prime T cells, we first examined the generation of tumor antigen‐specific CTLs. Since tumor antigen‐specific CTLs are primed by APCs in tumor draining lymph nodes (TdLNs) and then recruited to tumors through peripheral circulation,^[^ ^31^, ^32^ ^]^ we then evaluate the presence of tumor‐specific CTLs in the TdLNs and the peripheral blood of patients with high TMB. Examined by immunofluorescence staining (Figure 1F) and flow cytometric analysis (Figure 1G), the number of tumor‐specific CTLs in TdLNs and peripheral blood of TMB^hi^CTL^lo^ patients was much lower than that in the TMB^hi^CTL^hi^ group, suggesting that CD8^+^ T cells might not be properly primed in the TMB^hi^CTL^lo^ patients even in the presence of abundant tumor antigens.

Since priming of CD8^+^ T cells was carried out by mature cDC1 cells, which capture tumor antigens in the tumor sites and migrate to TdLNs to present antigens for T cell priming,^[^ ^33^ ^]^ we examined the number of cDC1 in both TdLN (CD11c^+^CD1a^+^) and tumor site (CD11c^+^CD141^+^) and found that the abundance of cDC1 was equivalent in TMB^hi^CTL^hi^ and TMB^hi^CTL^lo^ patients (Figure S1B,C, Supporting Information). Moreover, no significant difference of CD80 and CD86 expression in the cDC1 of TdLN, markers for cDC1 maturation,^[^ ^34^ ^]^ was observed between TMB^hi^CTL^hi^ and TMB^hi^CTL^lo^ groups (Figure S1D, Supporting Information), suggesting that the abundance and maturation of cDC1 were not the contributing factors for poor CD8^+^ T cell priming in TMB^hi^CTL^lo^ patients. To further evaluate the function of antigen cross‐presentation in the APCs, cDC1 were isolated from the tumor sites and TdLNs of TMB^hi^CTL^lo^ and TMB^hi^CTL^hi^ patients and co‐cultured with autologous naive CD8^+^ T cells, respectively. Interestingly, cDC1 from TMB^hi^CTL^lo^ patients could not efficiently prime naive CD8^+^ T cells, as the co‐cultured T cells exhibited much lower percentages of GZMB^+^ and MUC1‐pentamer^+^ CTLs (Figure 1H; Figure S1E, Supporting Information) and weaker cytotoxicity against autologous tumor cells (Figure S1F, Supporting Information), as compared with those co‐cultured with cDC1 from TMB^hi^CTL^hi^ tumors. These data suggested the antigen cross‐presentation of cDC1 in TMB^hi^CTL^lo^ tumors might be impaired.

### 2.2 Tumor Cells Suppressed cDC1 Antigen Cross‐Presentation by Checking Antigen Release From Endosomes to Cytoplasm

To evaluate the function of antigen uptake in TiDCs, we employed 3 µm latex beads for phagocytosis (Figure S2A, Supporting Information) and fluorescent dextran for endocytosis (Figure S2B, Supporting Information). No difference was observed regarding phagocytosis or endocytosis in TiDCs isolated from TMB^hi^CTL^hi^ and TMB^hi^CTL^lo^ patients (Figure S2A,B, Supporting Information). Moreover, upon being pulsed with Ovalbumin (OVA) antigen protein beads for 10 min and chased for 2 h,^[^ ^35^, ^36^ ^]^ similar amount of residual OVA protein on beads was detected in TiDCs from TMB^hi^CTL^hi^ and TMB^hi^CTL^lo^ tumors (Figure S2C, Supporting Information). These results suggested that the functions of antigen capturing and phagosomal degradation were not influenced in TiDCs from TMB^hi^CTL^lo^ tumors.

**Figure 2:**
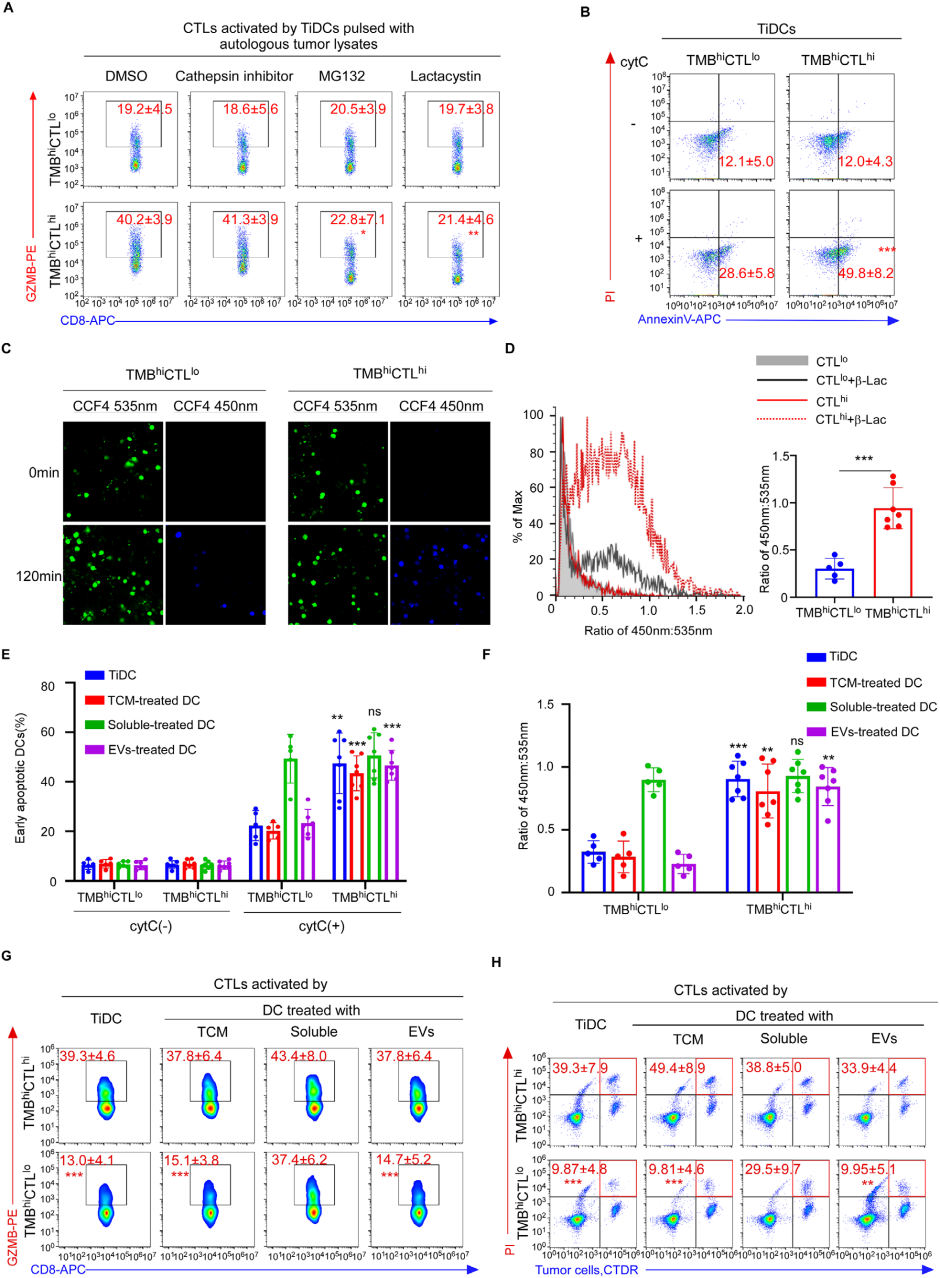
Tumor cells suppressed cDC1 antigen cross‐presentation by checking antigen release from endosomes to cytoplasm. A) Naive CD8^+^ T cells were primed by TiDCs of TMB^hi^CTL^hi^ (*n* =  3) or TMB^hi^CTL^lo^ (*n* =  3) breast cancer patients with or without lactacystin, MG132 or cathepsin inhibitor. Representative flow cytometric plots and quantification of percentages of GZMB staining in the in vitro primed CD8^+^ T cells. B) Representative flow cytometric plots and quantification of AnnexinV^+^propidium iodide (PI)^−^ staining in TiDCs isolated from TMB^hi^CTL^hi^ (*n* = 7) or TMB^hi^CTL^lo^ (*n* = 5) breast cancer patients and then treated with exogenous cytC. C,D) TiDCs from TMB^hi^CTL^hi^ (*n* = 7) or TMB^hi^CTL^lo^ (*n* = 5) breast cancer patients were loaded with CCF4 and then fed with β‐lactamase (β‐lac) for 120 min. Fluorescence resonance energy transfer (FRET) was detected by fluorescence microscope (C) and flow cytometry (D). 405 nm wavelength was used for excitation of CCF4. Emission at 535 nm (FRET green/intact probe) and 450 nm (deFRET blue/cleaved probe) were analyzed. C) Representative fluorescence images of FRET of CCF4, Scale bar, 5 µm. D) Representative flow cytometric plots and quantification of β‐lactamase activity represented by ratiometric values (450 nm: 535 nm) of CCF4. E–H) TiDCs were isolated from TMB^hi^CTL^hi^ (*n* = 7) or TMB^hi^CTL^lo^ (*n* = 5) breast cancer patients. Monocyte‐derived DCs (moDCs) were treated with bulk tumor conditioned media (TCM), soluble fraction of TCM or EVs of TMB^hi^CTL^hi^ (*n* = 7) or TMB^hi^CTL^lo^ (*n* = 5) breast tumor, respectively. E) Cell apoptosis induced by endosome‐cytosol export of internalized cytC in TiDCs or moDCs with indicated treatments were determined by flow cytometry. Percentages of AnnexinV^+^PI^−^ DCs are shown. F) FRET of CCF4 induced by endosome‐cytosol export of β‐lactamase in TiDCs or moDCs with indicated treatments were determined by flow cytometry. Ratio of 450 nm:535 nm is shown. G,H) Naive CD8^+^ T cells were primed by TiDCs or moDCs treated as (E). G) Representative flow cytometric plots and quantification of percentages of GZMB staining in the in vitro primed CD8^+^ T cells. H) The death of tumor cells pre‐stained with CellTracker Deep Red (CTDR) induced by in vitro primed CTLs was examined by PI uptake through flow cytometry. Numbers in the plots indicate percentages of PI^+^ tumor cells. Results are mean ± s.d. of independent experiments producing similar results. ns, not significant, *P* > 0.05, *P < 0.05, **P < 0.01, ***P < 0.001, compared with TMB^hi^CTL^lo^ group with indicated treatments, were determined by two‐tailed Student's t test (A, B, D–H).

To discriminate the cytosolic or vacuolar pathway for TiDCs to cross‐present tumor antigens, we found that activation of the co‐cultured CD8^+^ T cells was significantly inhibited by lactacystin and MG132, inhibitors for the cytosolic pathway,^[^ ^37^, ^38^ ^]^ but not by the cathepsin inhibitor that inhibits the vacuolar pathway (**Figure** **2**A),^[^ ^39^ ^]^ confirming that TiDCs exploited a cytosolic pathway to cross‐present tumor antigens for CD8^+^ T cell priming as reported previously.^[^ ^40^ ^]^ Since antigen release from endo/phagosomes into cytoplasm is a key step for the process of cross‐presentation via MHC‐I molecules,^[^ ^40^ ^]^ we next detected antigen release from endo/phagosomes to cytoplasm in TiDCs using cytochrome C (cytC) releasing assay, as the internalized cytC could induce apoptosis upon releasing from endosomes to cytoplasm.^[^ ^41^ ^]^ Interestingly, we observed that TiDC apoptosis induced by cytC translocation was lower in TMB^hi^CTL^lo^ patients, compared with the ones in TMB^hi^CTL^hi^ patients, suggesting that antigen release from endo/phagosomes to cytoplasm in cDC1 was suppressed in TMB^hi^CTL^lo^ patients (Figure 2B). To confirm this finding, we loaded TiDCs with a cytosolic CCF4 probe consisting of two fluorophores linked by a β‐lactam ring, which was switched from emitting green to blue fluorescence upon exposure to the internalized β‐lactamase that was translocated from endosome to cytoplasm.^[^ ^37^, ^42^ ^]^ Determined by fluorescence microscope (Figure 2C) and flow cytometric analysis of the fluorescence resonance energy transfer (FRET) (Figure 2D), we found that in the same time frame, fewer TiDCs from TMB^hi^CTL^lo^ patients underwent transition from green (535 nm) to blue (450 nm) fluorescence, further suggesting the suppression of antigen release to cytoplasm in APCs (Figure 2C,D).

To further investigate whether the functional suppression in TiDCs was mediated by tumor cells, we treated normal monocyte‐derived DCs (mo‐DCs) with the bulk tumor conditioned media (TCM) from TMB^hi^CTL^hi^ and TMB^hi^CTL^lo^ tumors, respectively. We found that TCM from the TMB^hi^CTL^lo^ tumors, rather than the TMB^hi^CTL^hi^ ones, significantly inhibited antigen release to cytoplasm in DCs (Figure 2E,F). This was consistent with the suppression of priming of the co‐cultured CTLs (Figure 2G), as well as the inhibition of cytotoxic effects against tumor cells (Figure 2H). Furthermore, upon separating the supernatant of TCM into soluble and extracellular vesicles (EVs) components (Figure S2D,E, Supporting Information), we found that the EVs, but not soluble components, from the TMB^hi^CTL^lo^ tumors significantly inhibited antigen release to cytoplasm in the DCs (Figure 2E,F) and dramatically suppressed their ability to activate the co‐cultured CTLs (Figure 2G,H). Moreover, EV release was inhibited in the MDA‐MB‐468 TNBC cell line through ALIX knockdown (Figure S2F, Supporting Information),^[^ ^43^ ^]^ which restored the antigen export to the cytoplasm, as indicated by enhanced apoptosis of DCs upon cytC treatment (Figure S2G, Supporting Information). This ultimately facilitated DC‐mediated cross‐priming of CD8^+^ T cells, leading to increased GZMB production (Figure S2H, Supporting Information) and effective lysis of co‐cultured tumor cells (Figure S2I, Supporting Information), thereby confirming that tumor‐derived EVs mediate this immunosuppressive effect. Collectively, these findings suggest that tumor cells suppress antigen translocation from endo/phagosomes to cytoplasm in cDC1 via EVs.

### 2.3 Tumor EVs Shuttled CDC37 to DC Endosomes to Suppress Antigen Cross‐Presentation

**Figure 3:**
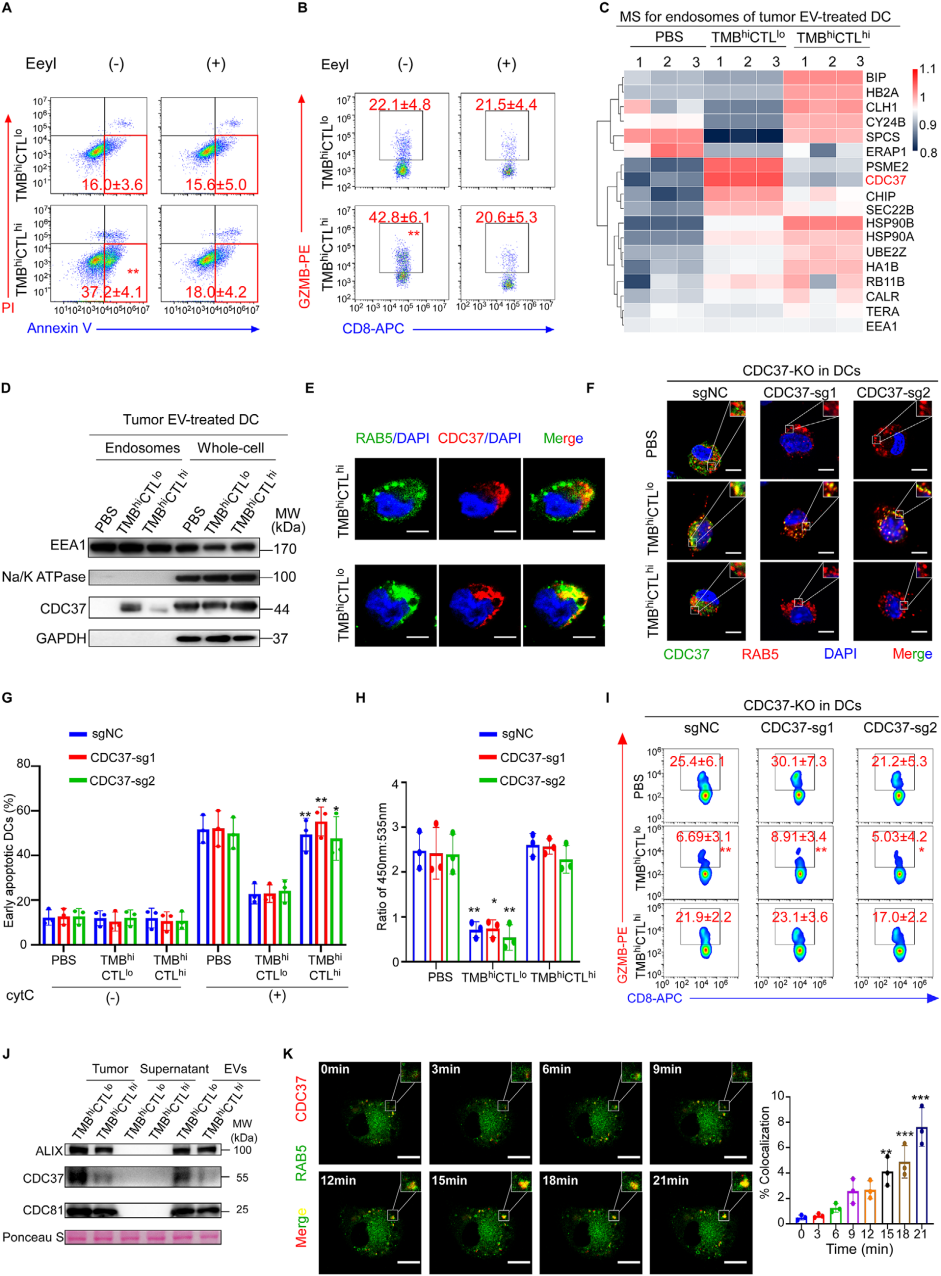
Tumor EVs shuttled CDC37 to DC endosomes to suppress antigen cross‐presentation. A,B) DCs were treated with EVs from TMB^hi^CTL^hi^ or TMB^hi^CTL^lo^ breat tumors, with or without the ERAD inhibitor Eeyarestatin I (EeyI). A) Representative flow cytometric plots and quantification of AnnexinV^+^PI^−^ DCs after exogenous cytC treatment. B) Representative flow cytometric plots and quantification of GZMB^+^CD8^+^T cells primed by DCs with indicated treatment. C,D) DCs were treated with PBS or EVs from TMB^hi^CTL^lo^ (*n* = 3) and TMB^hi^CTL^hi^ (*n* = 3) tumors, respectively. C) Heatmap displaying differentially expressed proteins in the endosomes of DCs with indicated treatment, determined by targeted mass spectrometry focusing on 18 proteins of interest, potentially related to ERAD in endosomes. Color scale, log_2_(fold change) normalized to endosome marker EEA1. D) Representative images of CDC37 expression in the endosomes and whole cell of DCs with indicated treatment, are shown by western blotting (*n* = 3 independent experiments). Na/K ATPase was used as a marker of cell membrane protein and EEA1 was used as a marker of endosomes. MW, molecular weight. E) Representative images of immunofluorescence staining for CDC37 and RAB5 in TiDCs of TMB^hi^CTL^lo^ (*n* = 3) and TMB^hi^CTL^hi^ (*n* = 3) breast tumor patients. Scale bar, 5 µm. Quantification is shown in Figure S3A (Supporting Information). F–I). DCs were transduced with control sgRNA (sgNC), sgRNAs targeting CDC37 (CDC37‐sg1, CDC37‐sg2) and then treated with EVs from TMB^hi^CTL^lo^ (*n* = 3) or TMB^hi^CTL^hi^ (*n* = 3) breast tumor, respectively. F) Representative immunofluorescence images of CDC37 and RAB5 co‐staining in DCs. Scale bar, 5 µm. Quantification is shown in Figure S3D (Supporting Information). G) Cell apoptosis induced by endosome‐cytosol export of internalized cytC in DCs were determined by flow cytometry. Percentages of AnnexinV^+^PI^−^ DCs are shown. H) Quantification of β‐lactamase activity represented by ratiometric values (450 nm: 535 nm) of CCF4 in DCs, determined by flow cytometry. I) DCs with indicated treatment were pulsed with tumor lysates and co‐cultured with autologous naive CD8^+^ T cells, respectively. Representative flow cytometric plots and quantification of the percentages of GZMB releasing CD8^+^ T cells. J) Representative images of CDC37 expression in tumor tissue, or in the supernatant and EV components of TCM from TMB^hi^CTL^lo^ (*n* = 3) and TMB^hi^CTL^hi^ (*n* = 3) breast cancer patients, are shown by western blotting (*n* = 3 independent experiments). ALIX and CD81 represent the markers of EVs, and ponceau S represents total protein loading for normalization. K) Representative live imaging for GFP‐RAB5 overexpressing DCs treated with EVs of mCherry‐CDC37 overexpressing SKBR3 cancer cells during 21 min (*n* = 3 independent experiments). Scale bar, 5 µm. Quantification of co‐localization of CDC37 and RAB5 at indicated time point is shown. Results are mean ± s.d. of independent experiments producing similar results. *P < 0.05, **P < 0.01, ***P < 0.001, compared with TMB^hi^CTL^lo^ group (A,B), DCs transduced with indicated sgRNA and treated with PBS (G‐I), or DCs without EV treatment (0 min) (K), were determined by two‐tailed Student's t test (A, B), or two‐tailed one‐way ANOVA with Dunnett's multiple‐comparisons test (G–I,K).

Endoplasmic reticulum associated degradation (ERAD), a process for unfolded or misfolded protein degradation, was exploited by cytoplasmic antigen translocation for cross‐presentation.^[^ ^44^, ^45^ ^]^ To investigate whether tumor EVs suppressed cytoplasmic antigen release by affecting the ERAD process in DCs, DCs were pre‐treated with Eeyarestatin I (EeyI), an inhibitor for ERAD pathway,^[^ ^38^ ^]^ followed by exposure to tumor EVs. Notably, EeyI treatment significantly suppressed the antigen translocation from endosome to cytoplasm, as determined by cytC releasing assay, in DCs exposed to TMB^hi^CTL^hi^ EVs (**Figure** **3**A), leading to reduced GZMB production in the co‐cultured CD8^+^ T cells (Figure 3B). In contrast, DCs treated with TMB^hi^CTL^lo^ tumor EVs showed no further changes following EeyI treatment, indicating that TMB^hi^CTL^lo^ tumor EVs suppressed cytoplasmic antigen release via an ERAD‐dependent pathway. Furthermore, we isolated endosomal proteins from mo‐DCs treated with EVs of TMB^hi^CTL^hi^ or TMB^hi^CTL^lo^ tumors, followed by targeted mass spectrometry (MS) analysis using 18 ERAD‐related proteins as candidate targets.^[^ ^44^, ^45^, ^46^ ^]^ Our findings revealed that CDC37, an oncogene responsible for proper protein folding in tumor cells,^[^ ^47^ ^]^ was the most prominent differentially expressed protein and was significantly upregulated in the endosomes of DCs treated with TMB^hi^CTL^lo^ tumor EVs, as compared with the endosomes of DCs treated with PBS or TMB^hi^CTL^hi^ EVs (Figure 3C). This was further validated by western blotting using whole‐cell or endosomal proteins (Figure 3D). Moreover, we evaluated the subcellular localization of CDC37 in TiDCs by co‐immunostaining with RAB5, an early endo/phagosome marker, respectively. Interestingly, CDC37 protein level was equivalent in the cytoplasm of TiDCs isolated from TMB^hi^CTL^hi^ and TMB^hi^CTL^lo^ tumors, while it was much higher in the endo/phagosomes of TiDCs from TMB^hi^CTL^lo^ than TMB^hi^CTL^hi^ tumors (Figure 3E; Figure S3A, Supporting Information). These data insinuated that TMB^hi^CTL^lo^ tumor EVs might not influence the intrinsic CDC37 expression in the cytoplasm of DCs, but affected its presence in the endo/phagosomes of DCs.

Furthermore, we employed single‐guide (sg) RNAs targeting CDC37 in DCs (Figure S3B, Supporting Information) treated with tumor EVs (Figure S3C, Supporting Information) and found that the endogenous cytoplasmic CDC37, rather than CDC37 in the endo/phagosomes, as denoted by CDC37 and RAB5 co‐immunostaining, was effectively silenced in DCs (Figure 3F; Figure S3D, Supporting Information). Functionally, silencing CDC37 in DCs did not influence the cytosolic antigen release, as assessed by cytC translocation (Figure 3G) and CCF4 cleavage assay (Figure 3H), nor did it influence the activation of the co‐cultured CD8^+^ T cells, noted as CD69 expression, GZMB secretion and cytotoxicity (Figure 3I; Figure S3E,F, Supporting Information), suggesting that intrinsic CDC37 expression in DCs did not affect their function of antigen cross‐presentation. Moreover, treating CDC37 knockout (CDC37^KO^) DCs with EVs isolated from the TMB^hi^CTL^lo^, but not from the TMB^hi^CTL^hi^ tumors, increased CDC37 level in their endo/phagosomes (Figure 3F; Figure S3D, Supporting Information). Functionally, treatment with TMB^hi^CTL^lo^ EVs effectively inhibited cytosolic antigen release in the DCs with or without CDC37 knockout (Figure 3G,H) and impaired their capability to prime the co‐cultured CD8^+^ T cells, as their CD69 expression, GZMB production and cytotoxicity to autologous tumor cells were greatly reduced (Figure 3I; Figure S3E,F, Supporting Information). In contrast, cytosolic antigen release and T cell priming capability of CDC37^KO^ DCs treated with TMB^hi^CTL^hi^ EVs remained unchanged (Figure 3G–I; Figure S3E,F, Supporting Information). Collectively, these data suggested that CDC37 in the endo/phagosomes of DCs might be shuttled from tumor EVs and responsible for the suppression of cytosolic antigen release and antigen cross‐presentation of DCs.

We next confirmed the presence of CDC37 in TMB^hi^CTL^lo^ EVs rather than in the TCM soluble constituents, and its level was much higher than that in TMB^hi^CTL^hi^ EVs (Figure 3J). To further investigate the source of EV‐packaged CDC37, we analyzed publicly available single‐cell RNA sequencing (scRNA‐seq) data of breast cancer patients.^[^ ^48^ ^]^ In TNBC samples, we performed cell clustering across most of the cell populations, including tumor cells, normal epithelial cells, DCs, macrophages, fibroblasts, endothelial cells, T cells, B cells, and so on (Figure S3G, Supporting Information). We observed that CDC37 was highly expressed in tumor cells and endothelial cells, with significantly lower levels in fibroblasts, immune cells, normal epithelial cells and other cell types (Figure S3H, Supporting Information). Given that tumor cells are present in much greater numbers while endothelial cells are scarce, tumor cells serve as the primary source of CDC37. Consistent with scRNA‐seq analysis, immunofluorescence staining of tumor tissue sections showed that CDC37 was primarily expressed in the tumor cells (CK^+^), instead of macrophages (CD68^+^) or fibroblasts (α‐SMA^+^) (Figure S3I,J, Supporting Information). To track CDC37 shuttling from tumor cells to DCs, we enforced the expression of CDC37 linked with mCherry fluorescence (mCherry‐CDC37) into SKBR3 breast tumor cells, which expressed low level of CDC37 (Figure S3K, Supporting Information), and co‐cultured them with mo‐DCs. Flow cytometric analysis showed that the mCherry signals could be detected in DCs 16 h following co‐culturing (Figure S3L, Supporting Information). Furthermore, immunofluorescence staining demonstrated the presence of mCherry signal in the endo/phagosomes of DCs upon treatment with EVs from mCherry‐CDC37 overexpressing tumor cells (Figure S3M, Supporting Information). Monitored by live‐cell imaging, mCherry signal from the EVs of mCherry‐CDC37 overexpressing tumor cells was found present at 12 min and maintained in the early endosomes of RAB5‐GFP overexpressing moDCs, denoted by the colocalization of GFP and mCherry fluorescence signaling (Figure 3K; and Video S1, Supporting Information). Notably, CDC37 remained robustly colocalized with RAB5 for up to 2 h (Figure S3N, Supporting Information). Moreover, inhibitors for macropinocytosis (Cytochalasin D and EIPA)^[^ ^49^, ^50^ ^]^ or caveolin‐mediated endocytosis (Genistein and LY294002),^[^ ^51^, ^52^ ^]^ rather than inhibitors for clathrin‐mediated endocytosis (Chlorpromazine and Dynasore),^[^ ^53^, ^54^ ^]^ significantly suppressed mCherry‐CDC37‐containing tumor EV absorption in DCs (Figure S3O, Supporting Information), suggesting that CDC37‐containing EVs were internalized into DCs through macropinocytosis or caveolin‐mediated endocytosis. In the contrast, compared with DCs, other cell types within the tumor microenvironment, including B cells, T cells, and fibroblasts, showed minimal uptake of mCherry‐CDC37‐containing tumor EVs, while macrophages showed comparable uptake of these EVs (Figure S3P,Q, Supporting Information). However, CDC37‐containing tumor EVs had no effect on antigen cross‐presentation in macrophages (Figure S3R, Supporting Information), which might be due to the reliance of macrophages on the vacuolar pathway for antigen presentation, a pathway that does not involve cytosolic antigen release.^[^ ^55^, ^56^ ^]^ Collectively, these findings confirmed that CDC37 was shuttled from tumor cells to the endo/phagosomes of TiDCs via EVs.

### 2.4 Targeting CDC37 in Tumor Cells Promoted Antigen Cross‐Presentation in DCs

**Figure 4:**
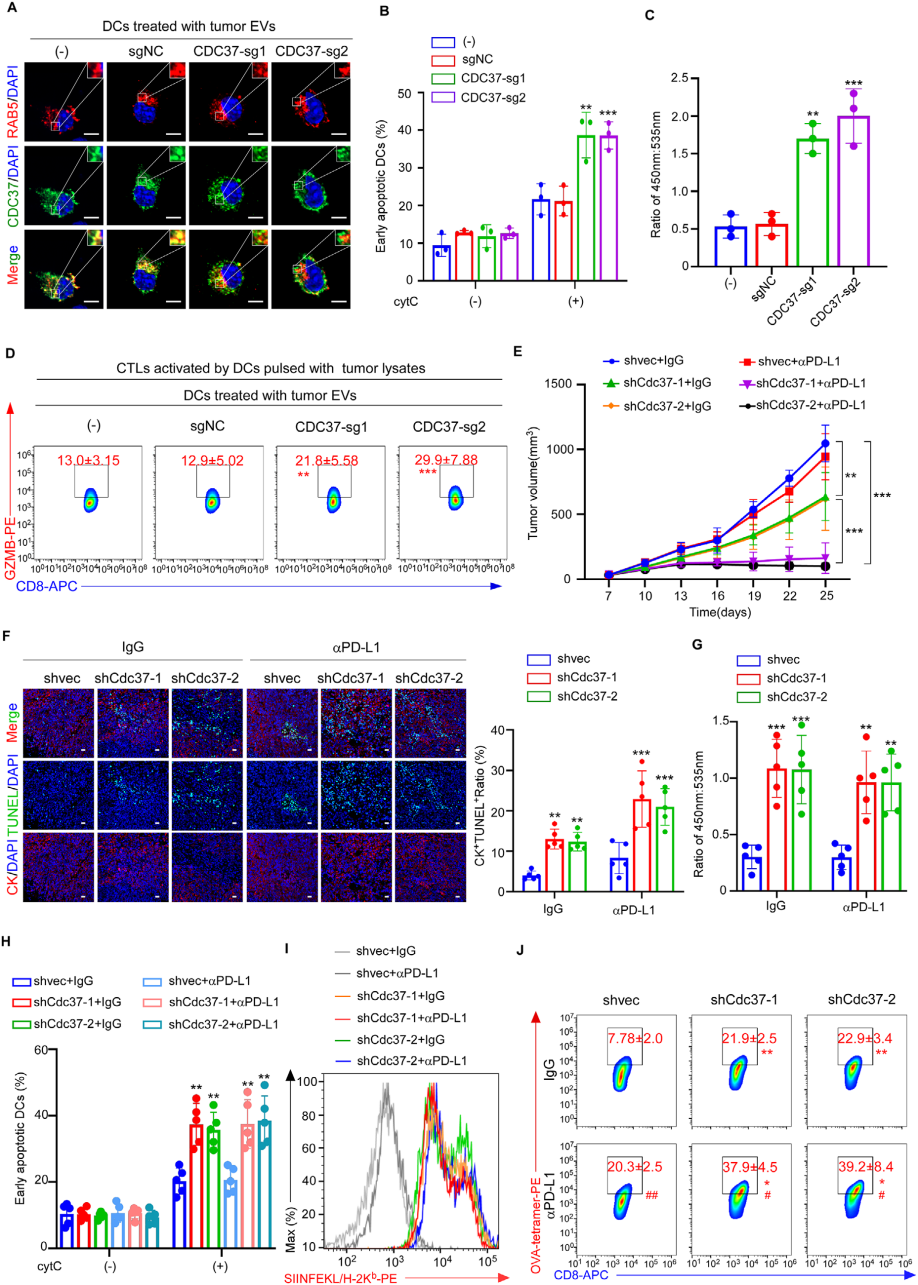
Targeting CDC37 in tumor cells promoted antigen cross‐presentation in DCs. A–D). DCs were treated with tumor EVs from wild type MDA‐MB‐468 breast cancer cells (‐) or MDA‐MB‐468 tumor cells transduced with sgNC, CDC37‐sg1, CDC37‐sg2, respectively. A) Representative images of immunofluorescence staining for CDC37 and RAB5 in DCs with indicated treatment (*n* = 3). Scale bar, 5 µm. Quantification is shown in Figure S4E (Supporting Information). B) Cell apoptosis induced by endosome‐cytosol export of internalized cytC in DCs was determined by flow cytometry. Percentages of AnnexinV^+^PI^−^ DCs are shown (*n* = 3). C) Quantification of FRET of CCF4 induced by export of β‐lactamase in DCs, determined by flow cytometric analysis of ratiometric values 450 nm:535 nm (*n* = 3). D) DCs treated with indicated tumor EVs were co‐cultured with autologous naive CD8^+^ T cells. Representative flow cytometric plots and quantification of the percentages of GZMB‐releasing CD8^+^ T cells (*n* = 3). E–J) EO771‐OVA mouse breast cancer cells transduced with shvec, shCdc37‐1 or shCdc37‐2 were inoculated to the mammary fat pads of immunocompetent syngeneic C57BL/6 mice, and intravenously administered the mice with anti‐PD‐L1 immunotherapy or IgG every three days initiated on the seventh day following tumor inoculation (*n* = 5 per group). E) Tumor volumes were monitored every 3 days after palpable tumor formation. **P < 0.01, ***P < 0.001, were determined by two‐tailed one‐way ANOVA with Tukey's multiple‐comparisons test. F) Representative immunofluorescence images and quantification of dead tumor cells denoted by co‐staining with CK and TUNEL in the harvested grafts (*n* = 5 per group). Scale bar, 50 µm. G) Quantification of β‐lactamase activity represented by ratiometric values (450 nm:535 nm) of CCF4 in TiDCs, determined by flow cytometry (*n* = 5 per group). H) Quantification of early cell apoptosis induced by endosome‐cytosol export of internalized cytC in TiDCs, determined by flow cytometry (*n* = 5 per group). I) Representative flow cytometric plots of SIINFEKL/H‐2K^b^ expression on TiDCs (*n* = 5 per group). Quantification is shown in Figure S4O (Supporting Information). J) Representative flow cytometric plots and quantification of the percentages of OVA‐tetramer staining in CD8^+^ T cells infiltrated in EO771‐OVA grafts with indicated treatment (*n* = 5 per group). Results are mean ± s.d. of independent experiments producing similar results. **P < 0.01, ***P < 0.001, compared with DCs treated with wild type MDA‐MB‐468 tumor EVs (‐) (B–D), or mice bearing shvec‐transduced EO771 tumors and receiving indicated treatment (F‐H), were determined by two‐tailed one‐way ANOVA with Dunnett's multiple‐comparisons test (B–D,F–H). *P < 0.05, **P < 0.01, compared with mice bearing shvec‐transduced tumors and receiving indicated treatment; ^#^ *P* < 0.05, ^##^ *P* < 0.01, compared with mice bearing indicated tumors and receiving IgG treatment, were determined by two‐tailed one‐way ANOVA with Tukey's multiple‐comparisons test (J).

To investigate the functional role of CDC37 in tumor cells, we knocked out CDC37 expression in MDA‐MB‐468 breast cancer cells that overexpress the molecule (Figure S4A, Supporting Information) and found that silencing CDC37 inhibited the proliferation, migration and invasion of the breast cancer cells (Figure S4B–D, Supporting Information). Moreover, silencing CDC37 in MDA‐MB‐468 cells abrogated its shuttling to the endo/phagosomes, but not the cytoplasm, of tumor EV‐treated DCs (**Figure** **4**A; Figure S4E, Supporting Information). Furthermore, cytC releasing assay (Figure 4B) and CCF4 cleavage assay (Figure 4C) demonstrated that cytosolic antigen release in DCs was restored by treating with EVs from the CDC37^KO^ tumor cells (Figure 4B,C). More importantly, silencing CDC37 expression in the breast tumor cells abrogated the suppression of antigen cross‐presentation in the tumor EV‐treated DCs, which could thus efficiently prime naive CD8^+^ T cells to produce GZMB (Figure 4D) and lyse tumor cells (Figure S4F, Supporting Information). In addition, tumor‐specific CD8^+^ T cells have comparable cytotoxicity against CDC37^WT^ and CDC37^KO^ tumor cells (Figure S4G, Supporting Information). In contrast, EVs released by SKBR3 breast cancer cells with CDC37 overexpression (OE) significantly suppressed antigen release into the cytosol (Figure S4H) and impaired subsequent antigen cross‐presentation in DCs (Figure S4I, Supporting Information). On the contrary, PSME2, a critical component of the immunoproteasome,^[^ ^57^ ^]^ and CHIP, an E3 ubiquitin ligase that facilitates proteasomal degradation,^[^ ^58^ ^]^ were also found to be upregulated in the endosomes of DCs treated with TMB^hi^CTL^lo^ tumor EVs, as determined by MS analysis and western blotting (Figure 3C; Figure S4J, Supporting Information). However, antigen export from endosome to cytoplasm remained unchanged in both PSME2^KO^ or CHIP^KO^ DCs (Figure S4K,L, Supporting Information), and DCs treated with PSME2^KO^ or CHIP^KO^ tumor EVs (Figure S4M, Supporting Information).

To investigate the role of CDC37 in immunotherapy responses in vivo, mouse breast cancer cells EO771 with CDC37 knockdown (CDC37^KD^) and OVA antigen overexpression were inoculated to the mammary fat pads of immunocompetent syngeneic C57BL/6 mice, and intraperitoneally administered the mice with anti‐PD‐L1 immunotherapy every three days initiated on the seventh day following tumor inoculation (Figure S4N, Supporting Information). Silencing CDC37 in tumor cells not only inhibited tumor growth (Figure 4E) as previously reported,^[^ ^59^ ^]^ but also enhanced anti‐PD‐L1 therapeutic efficacy, manifested by greater suppression of tumor growth (Figure 4E) and induction of tumor cell death (Figure 4F). Moreover, cytoplasmic antigen release in the TiDCs isolated from CDC37^KD^ tumor graft was much stronger than those from wildtype EO771‐OVA (CDC37^WT^) tumors, determined by CCF4 assays (Figure 4G) and cytC experiments (Figure 4H). More importantly, targeting CDC37 in the tumors enhanced antigen presentation of TiDCs, denoted by the presence of OVA peptide (257‐264)/MHC‐I complexes, SIINFEKL/H‐2K^b^, on DC surface (Figure 4I; Figure S4O, Supporting Information), as well as the enhanced infiltration of OVA(257‐264) tetramer^+^CD8^+^ T cells in the CDC37^KD^ tumor grafts (Figure 4J). Moreover, TiDCs isolated from CDC37^KD^ tumor were better in cross‐priming OT‐I CD8^+^ T cells in vitro, denoted by CD69 expression (Figure S4P, Supporting Information), and GZMB production (Figure S4Q, Supporting Information). Although anti‐PD‐L1 treatment did not further enhance antigen cross‐presentation in TiDCs (Figure 4G–I), it rendered more infiltration of OVA antigen‐specific CTLs (Figure 4J). Collectively, silencing CDC37 in tumor cells promoted antigen cross‐presentation in the TiDCs and thus enhanced ICB therapeutic efficacy.

### 2.5 CDC37 Checked Cytosolic Antigen Release by Binding to HSP90/Antigen Complex in DC Endosomes

**Figure 5:**
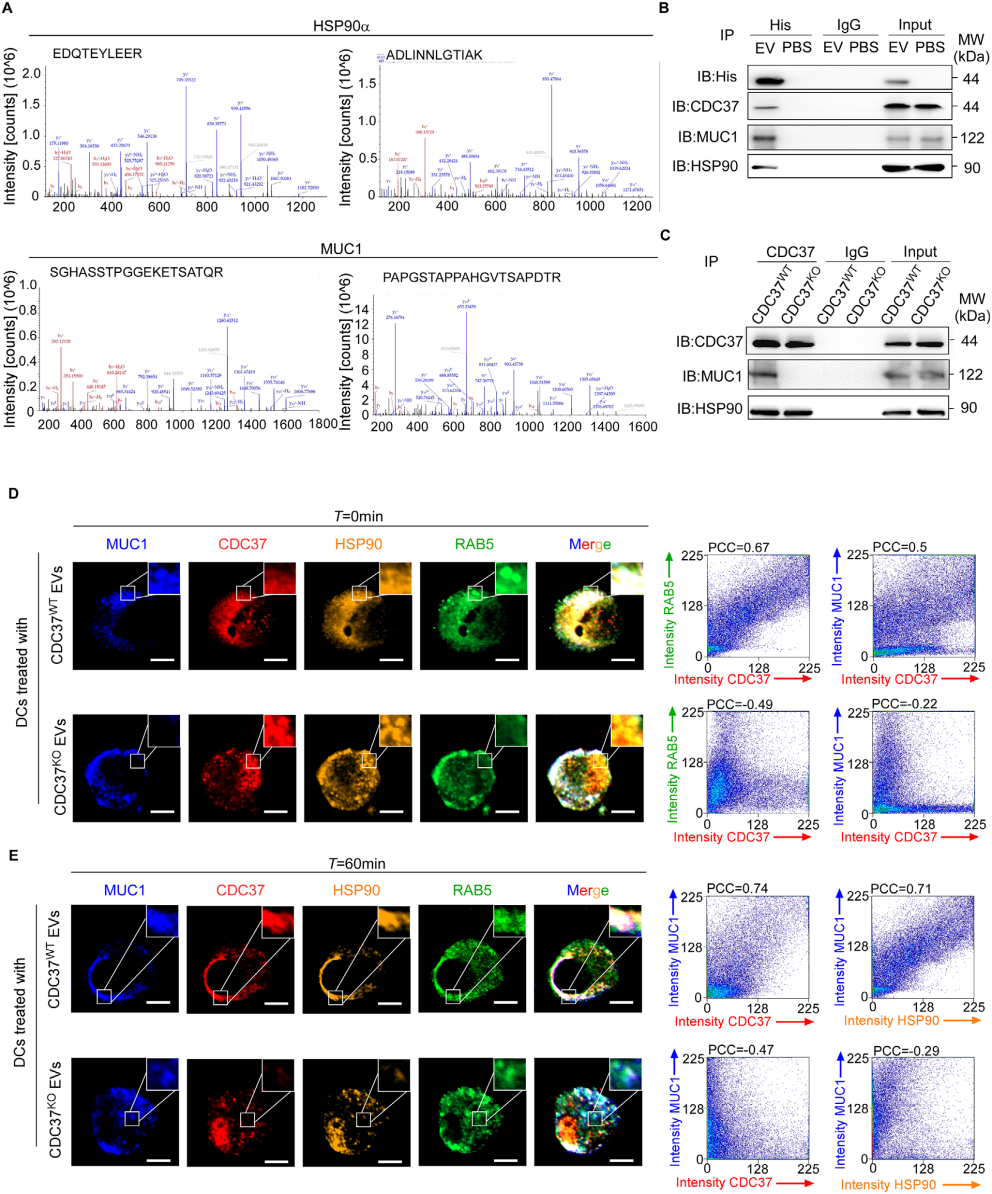
CDC37 checked cytosolic antigen release by binding to HSP90/antigen complex in DC endosomes. A–C) DCs were pulsed with tumor lysates (A) or MUC1 peptides (B,C) and treated with PBS, EVs from His‐CDC37‐overexpressing SKBR3 tumor cells (A‐B) or EVs from wild type (CDC37^WT^) MDA‐MB‐468 tumor cells or CDC37 knockout (CDC37^KO^) MDA‐MB‐468 tumor cells (C), respectively. A,B) Proteins binding to His‐CDC37 in DCs was detected by co‐immunoprecipitation (Co‐IP) with anti‐His antibody. (A) Co‐IP followed by mass spectrometry (MS) identified HSP90 and MUC1 as internalized CDC37 binding peptides (*n* = 3). (B) Binding of HSP90 and MUC1 to internalized His‐CDC37 in DCs was evaluated by Co‐IP with anti‐His antibody, followed by immunoblotting (*n* = 3 independent experiments). C) Binding of HSP90 and MUC1 to total CDC37 in DCs was evaluated by Co‐IP with anti‐CDC37 antibody, followed by immunoblotting (*n* = 3 independent experiments). D,E) DCs treated with EVs from CDC37^WT^ or CDC37^KO^ MDA‐MB‐468 tumor cells, respectively, were pulsed with MUC1 peptides for 1 h and then were washed and chased with medium for the indicated intervals. Representative images of immunofluorescence staining for CDC37, RAB5, HSP90, and MUC1 in DCs at 0 min (D) or 60 min (E) after being pulsed with MUC1 peptides. D) Scatterplots of CDC37 and RAB5 pixel intensities or CDC37 and MUC1 pixel intensities were shown with the Pearson correlation coefficient (PCC) above. E) Scatterplots of MUC1 and CDC37 pixel intensities or MUC1 and HSP90 pixel intensities were shown with the PCC above.

To further explore the molecular mechanisms how the EV‐shuttled CDC37 inhibited cytosolic antigen release in DCs, we treated moDCs with EVs from the SKBR3 cells with enforced expression of His‐tagged CDC37 (His‐CDC37). Interestingly, MS analysis of His‐CDC37 immunoprecipitates of DCs pulsed with tumor lysates revealed HSP90, an antigen co‐chaperone molecule,^[^ ^60^ ^]^ as the most prominent binding partner among numerous interacting proteins (Table S2, Supporting Information), along with a panel of tumor antigens, such as MUC1 (**Figure** **5**A). This was further validated by western blotting analysis (Figure 5B), suggesting that EV‐packaged CDC37 that was internalized into the DC endosomes could interact with the tumor antigen/HSP90 complex. Additionally, MUC1 tumor antigen peptide could be retrieved in the CDC37 immunoprecipitates of MUC1 peptide‐pulsed DCs pre‐treated with EVs from CDC37^WT^ but not CDC37^KO^ MDA‐MB‐468 breast cancer cells (Figure 5C), confirming that it was the EV‐packaged CDC37 internalized by DCs, but not the intrinsic CDC37 of the DCs, that bound tumor antigens.

**Figure 6:**
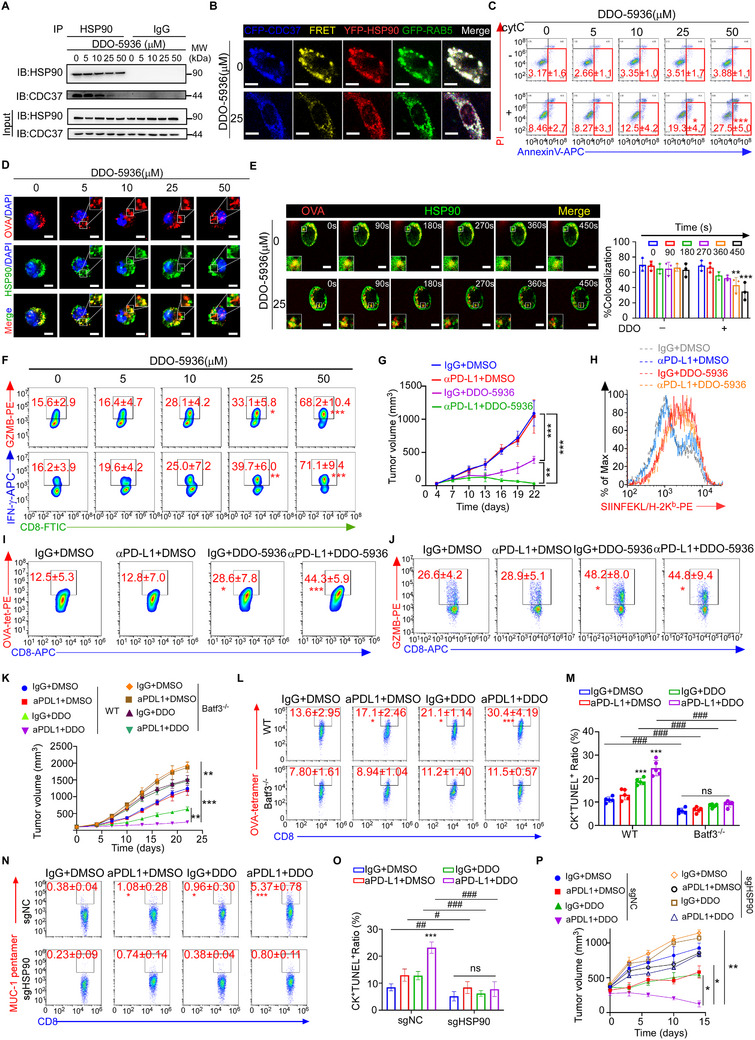
Blocking CDC37/HSP90 interaction promoted cytosolic antigen release and enhanced ICB efficacy. A) Binding of CDC37 to HSP90 in endosomes of DCs treated with MDA‐MB‐468 tumor EVs and different concentration of DDO‐5936 were determined by Co‐IP followed by immunoblotting (*n* = 3 independent experiments). B) DCs transfected with HSP90‐YFP and RAB5‐GFP plasmids were treated with CDC37‐CFP‐overexpressing SKBR3 tumor EVs and administrated with 25 µm DDO‐5936. The interaction between CDC37 and HSP90 in DC endosomes was detected by confocal fluorescence microscopy. Representative live‐cell imaging for FRET signals arising from the binding of CDC37‐CFP to HSP90‐YFP in the region of interest (ROI), based on RAB5 expression, by monitoring the fluorescence signals in the range of 550−620 nm with excitation of YFP at 405 nm (*n* = 3 independent experiments). Scale bar, 5µm. Quantification is shown in Figure S6B (Supporting Information). C) Representative flow cytometric plots and quantification of early cell apoptosis induced by endosome‐cytosol export of internalized cytC in DCs pre‐treated with tumor EVs from MDA‐MB‐468 and different concentration of DDO‐5936 (*n* = 3 independent experiments). Percentages of Annexin V^+^PI^−^ DCs are shown. D) DCs were pre‐treated with MDA‐MB‐468 tumor EVs and different concentrations of DDO‐5936 for 16 h, followed by the pulse with OVA antigen. Representative images of immunofluorescence staining for OVA and HSP90 in DCs (*n* = 3 independent experiments). Scale bar, 5µm. Quantification is shown in Figure S6C (Supporting Information). E) DCs transfected with HSP90‐GFP were treated with MDA‐MB‐468 tumor EVs and 25 µm DDO‐5936, followed by the pulse with mCherry‐OVA antigen. Representative live cell imaging for the binding of GFP‐HSP90 to mCherry‐OVA in DCs during OVA antigen treatment for 450 seconds (*n* = 3 independent experiments). Scale bar, 5 µm. Quantification of co‐localization of HSP90 and OVA at indicated time point is shown. F) DCs pre‐treated with tumor EVs from MDA‐MB‐468 and different concentrations of DDO‐5936 were pulsed with tumor lysates (*n* = 3 independent experiments). Representative flow cytometric plots and quantification of GZMB and IFN‐γ expression in the CD8^+^T cells primed by DCs. G–J) Mice bearing EO771‐OVA orthotopic grafts were treated with anti‐PD‐L1 antibody and/or DDO‐5936 (*n* = 5 per group). (G) Tumor growth curves of tumor‐bearing mice with indicated treatments. (H) Representative flow cytometric plots of SIINFEKL/H‐2K^b^ expression on TiDCs from tumor‐bearing mice with indicated treatments. Quantification is shown in Figure S6F (Supporting Information). I) Representative flow cytometric plots and quantification of the percentages of OVA‐tetramer staining in CD8^+^ T cells infiltrated in EO771‐OVA grafts with indicated treatment. J) Naive OT‐I CD8^+^T cells were co‐cultured with TiDCs isolated from mouse breast tumor. Representative flow cytometric plots and quantification of the percentages of GZMB releasing CD8^+^ T cells. K–M) EO771‐OVA mouse breast cancer cells were inoculated to the mammary fat pads of immunocompetent syngeneic C57BL/6 mice or Batf3^−/−^ transgenic mice, and intraperitoneally administered the mice with anti‐PD‐L1 immunotherapy or IgG every three days along with daily DDO‐5936 treatment, initiated on the seventh day following tumor inoculation (n = 5 per group). K) Tumor volumes were monitored every 3 days after palpable tumor formation. L) Representative flow cytometric plots and quantification of tumor‐specific OVA‐tetramer⁺ CD8⁺ T cells. Quantification is shown in Figure S6H (Supporting Information). M) Quantification of dead tumor cells denoted by co‐staining with CK and TUNEL in the harvested grafts. Representative immunofluorescence images are shown in S6I. N–P) NSG mice bearing patient‐derived xenografts (PDXs) from TMB^hi^CTL^lo^ patient with high CDC37 tumors were transfused with autologous tumor lysate‐pulsed DCs transduced with sgNC or sgHSP90 together with CD8^+^ T cells, followed by treatment with DDO‐5936, anti‐PD‐L1, or the combination (*n* = 3 per group). N) Representative flow cytometric plots and quantification of tumor‐specific MUC1‐pentamer⁺ CD8⁺ T cells. Quantification is shown in Figure S6J (Supporting Information). O) Quantification of dead tumor cells denoted by co‐staining with CK and TUNEL in the harvested grafts. Representative immunofluorescence images are shown in S6K. P) Tumor volumes were monitored every 3 days after palpable tumor formation. Results are mean ± s.d. of independent experiments producing similar results. *P < 0.05, **P < 0.01, ***P < 0.001, compared with DCs without DDO‐5936 (0 µm) (C, F), or tumor‐bearing mice treated with IgG and DMSO (IgG+DMSO) (**I**, J, L‐O), were determined by two‐tailed one‐way ANOVA with Dunnett's multiple‐comparisons test (C, F, I, J, L–O). *P < 0.05, **P < 0.01, were determined by two‐tailed one‐way ANOVA with Tukey's multiple‐comparisons test (G, K, P). ##*P* < 0.01, ###P < 0.001, compared with WT (M), or sgNC group(O), under the indicated treatment, were determined by two‐tailed one‐way ANOVA with Tukey's multiple‐comparisons test.

Consistently, immunofluorescence staining demonstrated most of the co‐localization of MUC1, CDC37, and HSP90 was observed in the early endo/phagosomes, stained with RAB5, of MUC1 peptide‐pulsed DCs pre‐treated with EVs from CDC37^WT^ MDA‐MB‐468 cells (55.7±8.0%) (Figure 5D), while only a small proportion of the MUC1 antigen co‐localized with HSP90 and CDC37 in the cytosol (8.4±3.1%) (Figure 5D). Furthermore, dynamic exposure revealed that MUC1 antigen peptide was locked in the complex with HSP90 and CDC37 in the tumor EV‐treated DCs’ endo/phagosomes for at least 60 min (Figure 5E). Knocking out CDC37 in MDA‐MB‐468 cells significantly reduced the presence of CDC37 in the MUC1/HSP90 complex (Figure 5D), and sped up the cytoplasmic antigen release (Figure 5E). Finally, co‐localization of CDC37 and HSP90 was confirmed by the FRET‐based immunofluorescence imaging between YFP‐HSP90, transfected in MoDCs, and CFP‐CDC37, transfected in CDC37^lo^ SKBR3 breast cancer cells, when EVs from the SKBR3 cells were extracted to treat the DCs (**Figure** **6**B).

To further investigate the role of CDC37/HSP90 complex on antigen cross‐presentation, we knocked out HSP90 in mo‐DCs pulsed with tumor lysates (Figure S5A, Supporting Information), followed by tumor EV treatment. Assessing the cytC translocation‐induced cell death of DCs (Figure S5B, Supporting Information) and the GZMB production from the co‐cultured CD8^+^ T cells (Figure S5C, Supporting Information), we found that the cytosolic antigen release and antigen cross‐presentation were markedly impaired in HSP90^KO^ DCs. Importantly, this impairment was not further exacerbated by the addition of tumor EVs containing CDC37 (Figure S5B,C, Supporting Information), demonstrating that CDC37 affects antigen cross‐presentation by modulating HSP90 function. Collectively, these findings suggested that CDC37 shuttled from tumor cells into DC endo/phagosomes via EVs bound the antigen/HSP90 complex and possibly locked the antigen from cytosolic release.

### 2.6 Blocking CDC37/HSP90 Interaction Promoted Cytosolic Antigen Release and Enhanced ICB Efficacy

To evaluate the therapeutic effects of targeting CDC37/HSP90 interaction, we employed a small molecule inhibitor blocking the binding of HSP90 and CDC37, DDO‐5936.^[^ ^61^ ^]^ Immunoblotting of the HSP90‐immunoprecipitates from DC endosomes (Figure 6A; Figure S6A, Supporting Information) and FRET‐based fluorescence imaging (Figure 6B; Figure S6B, Supporting Information) demonstrated that DDO‐5936 treatment inhibited the binding of HSP90 and the internalized CDC37 in the endo/phagosomes of DCs treated with EVs of CFP‐CDC37‐overexpressing SKBR3 or MDA‐MB‐468 (Figure 6A,B; Figure S6A,B, Supporting Information). Consistently, after DCs were pulsed with OVA antigen and treated by MDA‐MB‐468 EVs, DDO‐5936 treatment enhanced cytosolic antigen release (Figure 6C) by reducing OVA/HSP90 co‐localization in the endo/phagosomes of the DCs (Figure 6D; Figure S6C, Supporting Information). Moreover, live cell imaging demonstrated that DDO‐5936 accelerated the dissociation of GFP‐HSP90 and mCherry‐OVA antigen peptide in the endo/phagosomes of the GFP‐HSP90 overexpressing DCs in the presence of MDA‐MB‐468 EVs (Figure 6E; and Videos S2 and S3, Supporting Information). More importantly, DDO‐5936 treatment promoted the ability of DCs to prime CD8^+^ T cells, as the production of IFN‐γ and GZMB (Figure 6F) and the cytotoxicity of the in vitro primed CTLs were greatly enhanced (Figure S6D, Supporting Information). Therefore, the tumor EV‐packaged CDC37 shuttled into the endo/phagosomes of DCs inhibited antigen release into the DC cytosol by binding with HSP90 and locking the antigen/HSP90 complex.

In vivo, we explored whether combined application of ICB and DDO‐5936 may improve therapeutic efficacy in immunocompetent C57BL/6 mice bearing syngeneic EO771‐OVA tumors (Figure S6E, Supporting Information). Administration of DDO‐5936 alone by intraperitoneal injection could inhibit tumor growth and surprisingly, combined DDO‐5936 with monoclonal anti‐PD‐L1 antibody achieved more pronounced synergistic tumor inhibitory effects (Figure 6G). Moreover, DDO‐5936 alone could tremendously enhance anti‐tumor immunity in the tumors, as OVA antigen cross‐presentation in TiDCs (Figure 6H; Figure S6F, Supporting Information), infiltration of OVA‐specific CTLs (Figure 6I) and tumor cell death were significantly increased (Figure S6G, Supporting Information). Moreover, TiDC isolated from mice treated with DDO‐5936 were better in cross‐priming OT‐I CD8^+^ T cells in vitro, denoted by GZMB production (Figure 6J). Furthermore, DDO‐5936 combination with anti‐PD‐L1 revealed much stronger effects in increasing infiltration of OVA‐specific CTLs (Figure 6I), which resulted in more massive tumor cell death (Figure S6G, Supporting Information).

Given that DDO‐5936 alone could inhibit tumor growth by disrupting the interaction between CDC37 and HSP90 in tumor cells, we further investigated the involvement of immune regulation in this process. We utilized Batf3^−/−^ mice, which lack cDC1 responsible for antigen cross‐presentation,^[^ ^62^, ^63^ ^]^ transplanted with OVA‐EO771 tumor cells and treated with DDO‐5936 and anti‐PD‐L1. Compared with wild‐type mice, DDO‐5936 monotherapy modestly inhibited tumor growth in Batf3^−/−^ mice, but did not enhance the efficacy of anti‐PD‐L1 (Figure 6K). Flow cytometric analysis confirmed that DDO‐5936, either alone or in combination with anti‐PD‐L1, failed to increase tumor‐specific CTLs (Figure 6L; Figure S6H, Supporting Information) and augment CTL‐mediated cytotoxicity (Figure 6M; Figure S6I, Supporting Information) in Batf3^−/−^ mice. These findings thus underscore that the therapeutic benefit of DDO5936 also critically depends on cDC1‐mediated cross‐presentation.

To further validate the role of CDC37‐HSP90 interaction in antigen cross‐presentation by DCs in humanized mice, we employed an immunodeficient NOD scid γ ‐ null (NSG) mouse model which lacks functional T and B cells,^[^ ^64^ ^]^ engrafted with patient‐derived xenografts (PDXs) from a TMB^hi^CTL^lo^ breast cancer patient with high CDC37 expression. These mice received adoptive transfer of autologous CD8⁺ T cells together with DCs transfected with sgRNAs targeting a negative control (sgNC‐DCs) or HSP90 (HSP90^KO^‐DCs), followed by treatment with DDO‐5936 and anti‐PD‐L1. In mice receiving sgNC‐DCs, DDO‐5936 treatment alone increased the infiltration of tumor‐specific CTLs (Figure 6N; Figure S6J, Supporting Information), leading to enhanced tumor cell death (Figure 6O; Figure S6K, Supporting Information) and effective tumor control (Figure 6P), which could be significantly augmented by combining DDO‐5936 with anti‐PD‐L1 antibody (Figure 6N–P; Figure S6J,K, Supporting Information). In comparison, DDO‐5936 monotherapy exerted only modest tumor growth inhibition in mice receiving HSP90^KO^‐DCs, and failed to enhance the efficacy of anti‐PD‐L1 (Figure 6O,P; Figure S6K, Supporting Information). However, neither DDO‐5936 monotherapy, anti‐PD‐L1 monotherapy, nor their combination had any impact on tumor‐specific CTL infiltration in mice receiving HSP90^KO^‐DCs (Figure 6N; Figure S6J, Supporting Information). These data highlight that the antitumor efficacy of DDO‐5936 critically depends on functional HSP90‐CDC37 complexes in DCs.

### 2.7 CDC37 Expression was Associated with ICB Therapeutic Response of Breast Cancer Patients

**Figure 7:**
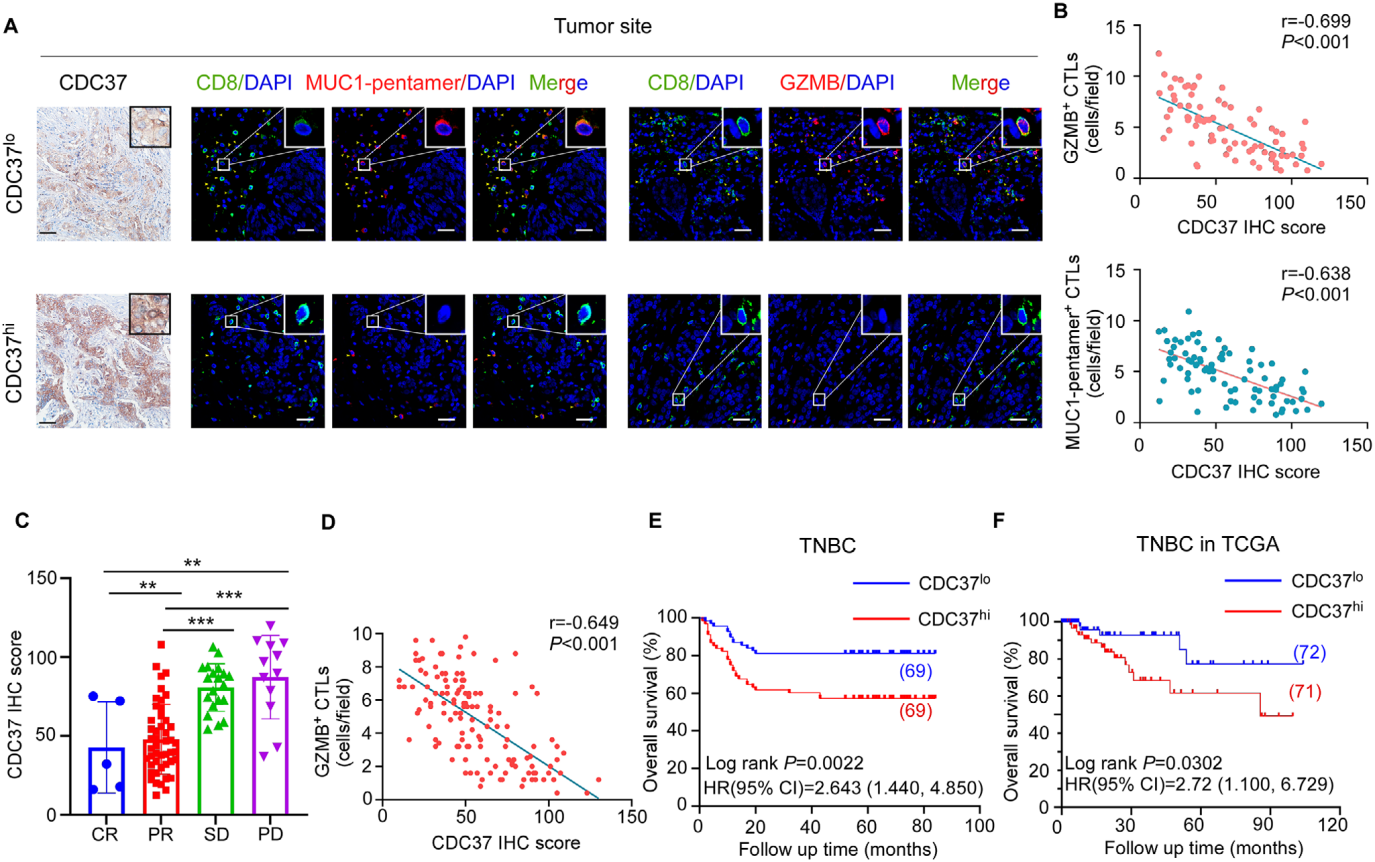
CDC37 expression was associated with ICB therapeutic response of breast cancer patients. A–C) TNBC patients who received ICB therapy were employed to detect CDC37 expression by immunohistochemical (IHC) staining in their pre‐treatment specimens and correlate CDC37 IHC scores to the infiltration of tumor‐specific CTLs and ICB efficacy (*n* = 84). A) Representative images of IHC staining for CDC37 and immunofluorescence staining for GZMB^+^CD8^+^ and MUC1‐pentamer^+^CD8^+^ T cells in tumors. Boxes denote the area of higher magnification images shown at the top right corner. Scale bar, 50 µm. B) Correlation between CDC37 IHC scores and the count of CD8^+^GZMB^+^ T cells or CD8^+^MUC1‐pentamer^+^ T cells in tumor sites of breast cancer patients, respectively (*n* = 84, Pearson's correlation coefficient r and two‐tailed *P* value are shown). C) The CDC37 IHC scores in the tumor site of TNBC patients with different therapeutic responses to ICB (mean ± s.d). CR, complete response, *n* = 5; PR, partial response, *n* = 46; SD, stable disease, n = 21; PD, progressive disease, *n* = 12. **P < 0.01; ***P < 0.001 by two‐tailed one‐way ANOVA with Tukey's multiple‐comparisons test. D). Correlation between CDC37 IHC scores and the count of CD8^+^GZMB^+^ T cells in TNBC patients (*n* = 138, Pearson's correlation coefficient r and two‐tailed *P* value are shown). E). Kaplan‐Meier curves for overall survival in TNBC patients with high (CDC37^hi^, >50.5, *n* = 69) and low (CDC37^lo^, <50.5, *n* = 69) CDC37 expression. Log‐rank *P*, hazard ratio (HR) and 95% confidence interval (95% CI) are shown. F). Kaplan‐Meier curves for overall survival in TNBC patients from TCGA dataset with high (>157.1, *n* = 71) and low CDC37 expression (<157.1, *n* = 72). Log‐rank *P*, HR and 95% CI are shown.

Clinically, we detected CDC37 expression in tumor slices of 137 cases of breast cancer patients by immunohistochemical staining (IHC) and observed that CDC37 IHC score was similar in various molecular subtypes of breast cancer (Figure S7A, Supporting Information), which was further validated by TCGA database (Figure S7B, Supporting Information). Then in the previous cohort of 84 cases of TNBC patients receiving ICB therapy in Sun‐Yat Sen Memorial Hospital, CDC37 IHC score was negatively associated with the infiltration of tumor‐specific CTLs, denoted by MUC1‐pentamer^+^CD8^+^ T cells (Pearson r = −0.638, *P* < 0.001) or GZMB^+^CD8^+^ T cells (Pearson r = −0.699, *P* < 0.001) (**Figure** **7**A,B). Also, the number of tumor‐specific CD8^+^ T cells in the TdLNs was negatively correlated with CDC37 expression in the primary tumors (Pearson r = −0.547, *P* < 0.001) (Figure S7C,D, Supporting Information). More importantly, CDC37 expression in the primary tumors was much higher in TNBC patients with poorer response to ICB therapy (Figure 7C). Based on the reanalysis of the publicly available scRNA‐seq dataset which comprised 12 cases of TNBC patients receiving neoadjuvant chemotherapy and ICB therapy,^[^ ^65^ ^]^ we found that CDC37 expression in tumor cells was higher in non‐responders than in responders to anti‐PD‐1 therapy (Figure S7E, Supporting Information).

To further evaluate the prognostic values of CDC37, we employed another cohort of 138 cases of TNBC patients from Sun‐Yat Sen Memorial hospital. Higher CDC37 expression (CDC37^hi^), demarcated according to the median value of CDC37 IHC score, was associated with larger tumor size (Table S3, Supporting Information), but negatively correlated with the infiltration of GZMB‐producing CTLs, denoted by immunofluorescence staining (Pearson r = −0.649, *P* < 0.001) (Figure 7D). Moreover, determined by Kaplan–Meier survival curves, higher CDC37 expression was significantly associated with shorter survival in TNBC patients (Figure 7E), which was confirmed by treatment‐naive TNBC cohorts from the TCGA database (Figure 7F). Based on univariable and multivariate Cox regression analyses, CDC37 was an independent prognostic factor for the patient outcome of TNBC (Table S4, Supporting Information). Collectively, CDC37 may potentially serve as a predictive biomarker for reduced efficacy of immunotherapy and worse patient prognosis of TNBC patients.

Furthermore, our analysis of TCGA pan‐cancer datasets revealed that CDC37 expression was generally elevated in tumors, compared with their normal counterparts (Figure S7F, Supporting Information). Notably, high CDC37 expression was significantly associated with poorer survival in lung adenocarcinoma (LUAD) and head and neck squamous cell carcinoma (HNSC) (Figure S7G, Supporting Information). Moreover, in patients with urothelial carcinoma (UC) and esophageal adenocarcinoma (ECA) who received anti–PD‐L1 immunotherapy, elevated CDC37 also predicted poorer prognosis (Figure S7H, Supporting Information). These findings align with our observations in TNBC, further supporting the potential prognostic relevance of CDC37 across multiple tumor types.

## 3 Discussion

The activated CTLs primed toward tumor antigens form the backbone of anti‐tumor immunity, which determines cancer patient outcome and immunotherapeutic efficacy.^[^ ^66^ ^]^ Effective generation of tumor‐antigen specific CTLs depends solely on tumor antigenicity and efficient antigen cross‐presentation by mature APCs.^[^ ^67^ ^]^ TMB, a conventional biomarker for antigenicity, has widely been used to correlate immune “hot” tumors with high CTL infiltration and to predict ICB therapeutic effects in miscellaneous types of solid tumors, but our present study and others^[^ ^10^, ^14^ ^]^ demonstrated a subset of cancer patients with high TMB lack tumor antigen‐specific CTL infiltration and responded poorly to ICB treatment. Our study went one step forward by revealing that tumor‐mediated inhibition of antigen cross‐presentation by cDC1 was the keystone contributing to defective generation of tumor‐specific CTLs and poor ICB therapeutic efficacy in breast cancer patients, even with high TMB and mature tumor‐associated DCs. Moreover, tumor cell derived CDC37, which served as a pivotal suppressor of antigen/chaperone release, was shuttled via EVs from tumor cells into cDC1 endo/phagosomes and inhibited the release of tumor antigens from their chaperone protein HSP90 in the endo/phagosomes. More importantly, CDC37 was a critical therapeutic target to restore efficient antigen cross‐presentation in the APCs and improve ICB effects.

DCs responsible for priming tumor antigen‐specific CTLs are recruited into the tumors, capture antigens released by live or dead tumor cells and undergo maturation upon migration to TdLNs, while they process the tumor antigens and cross‐present them to CD8^+^ T lymphocytes via MHC class‐I molecules.^[^ ^68^ ^]^ Recent studies have shown that tumor cells might impede DC maturation by inhibiting antigen uptake via a series of molecular events as modifying the “don't eat me”^[^ ^69^ ^]^ and “eat me” signals,^[^ ^70^ ^]^ masking the dead cell‐sensing receptors,^[^ ^71^, ^72^ ^]^ promoting the antigen degradation in endo/phagosomes of DCs^[^ ^73^ ^]^ and overexpressing the immune checkpoint molecules,^[^ ^74^ ^]^ which thus led to immune evasion and ICB treatment irresponsiveness. However, our present findings could not correlate cDC1 maturation status in the TdLNs with the abundance of tumor antigen‐specific CTLs, rather, we demonstrated that impaired cross‐presentation in the tumor‐associated cDC1 was responsible for ineffective CTL priming and the ensuing ICB ineffectiveness in breast cancer patients. In support of our findings, retarding antigen cross‐presentation in DCs by knocking out WDFY4 tremendously suppressed CD8^+^ T cell response against fibrosarcoma.^[^ ^75^ ^]^ Additionally, over‐activation of endoplasmic reticulum stress sensor, XBP1, by lipid peroxidation byproducts in the tumor‐associated DCs, induced abnormal lipid accumulation and inhibited cross‐priming of CD8^+^ T cells, resulting in ovarian cancer progression.^[^ ^76^ ^]^ Moreover, in a mouse model of lung carcinoma, DCs with accumulation of electrophilic oxidatively truncated lipids, which covalently bind to HSP70 and inhibit trafficking of the peptide‐MHC class I (pMHC) complexes from endosomes/lysosomes to the cell surface, are defective in stimulating CD8^+^ T cell responses.^[^ ^77^ ^]^ Furthermore, CY24B, a subunit of NOX2, regulates endosomal PH and antigen degradation, and its inhibition in DCs impairs tumor antigen cross‐presentation.^[^ ^78^, ^79^ ^]^ These findings suggest that molecules governing antigen cross‐presentation in tumor‐associated DCs may serve as important checkpoints for tumor immune evasion. In our study, mass spectrometry analysis identified a series of proteins that were differentially expressed in the endosomes of DCs following treatment with tumor‐derived EVs. Among them, endosomal CDC37 inhibits the release of tumor antigens from their chaperon HSP90 by binding to the HSP90/antigen complex to lock antigen in the endo/phagosome, representing a novel mechanism for modulating cross‐presentation, while the functions of other identified proteins, such as PSME2, CHIP, CY24B, and SPCS, remain to be elucidated.

Among the known antigen chaperone proteins, HSP90 is considered to play a pivotal role in helping antigen transfer to the protein channels of the endo/phagosomal membrane and release to cytoplasm for subsequent processing and presentation.^[^ ^60^ ^]^ Moreover, the Hsp90‐antigen complex internalized via endo/phagosomes promotes translocation of the chaperoned antigens for proteasomal degradation into peptides of appropriate size in the cytosol of APCs, facilitating their cross‐presentation via MHC‐I molecules.^[^ ^80^ ^]^ Here, we identified a critical suppressor of antigen/chaperone release, CDC37, which was previously reported to promote proliferation and enhance survival of prostate,^[^ ^81^ ^]^ hepatocellular,^[^ ^82^ ^]^ colorectal,^[^ ^82^ ^]^ and gastric cancer^[^ ^83^ ^]^ cells by facilitating HSP90 interaction with its client proteins, such as cyclin dependent kinases. In our present study, following the exclusion of other differentially expressed proteins, such as PSME2 and CHIP, on antigen release into the cytoplasm, we identified that tumor‐derived CDC37 could also serve as a checkpoint for antigen cross‐presentation in the APCs by binding with the HSP90/antigen complex in the endosomes of DCs. Another potential mechanism for the transfer of exogenous antigens into the cytosol is endo/phagosomal disruption, the pathway by which large exogenous molecules or complexes might enter the cytosol.^[^ ^40^, ^84^ ^]^ Indeed, we observed that CDC37‐antigen‐HSP90 complex in the cytosol, which might be derived from endo/phagosomal disruption, was significantly less than those in the endo/phagosome of DCs. However, these antigens were still locked in the CDC37‐antigen‐HSP90 complex and could not be released and processed, suggesting that as long as exogenous CDC37 remains in the complex with the HSP90‐antigen, it may still inhibit the dissociation of antigen from HSP90 in endo/phagosome or even in the cytosol, resulting in the inhibition of antigen processing and presentation. Thus, silencing CDC37 in tumor cells or blocking the interaction of HSP90 and CDC37 could not only inhibit proliferation of cancer cells,^[^ ^82^ ^]^ but also promote antigen cross‐presentation of DC. In vivo, targeting CDC37 in mouse breast cancer models could significantly enhance the efficacy of anti‐PD‐L1 therapy by inducing more antigen‐specific CTLs. In addition, CDC37‐HSP90 inhibitor exerts dual anti‐tumor effects by acting not only as a suppressor of tumor cell growth but also as a potent immunomodulator. Evidence from immunocompetent, immunodeficient, and humanized mouse models indicates that the primary therapeutic efficacy of CDC37‐HSP90 inhibitor relies on the enhanced antitumor immunity. Moreover, the synergy between CDC37‐HSP90 inhibitor and ICB highlights a potential novel strategy to improve the efficacy of ICB. Clinically, our findings, along with the analyses of publicly available online database, have demonstrated that high CDC37 expression correlated with low responsiveness to immunotherapy and worse prognosis across multiple cancer types. Therefore, CDC37 may serve as a therapeutic target to inhibit tumor growth and enhance anti‐tumor immune responses in turning immune “cold” tumors “hot”.

In recent years, a variety of immunotherapeutic strategies are being developed to turn immune “cold” tumors hot by increasing the number of tumor‐infiltrating and systemic tumor‐specific CTLs, which would greatly enhance ICB efficacy.^[^ ^85^, ^86^ ^]^ Among them, intratumor or intravenous administration of immune adjuvants or other strategies to enhance DC maturation and activation have been devised,^[^ ^18^ ^]^ including DNA repair inhibitors,^[^ ^87^ ^]^ cytokines,^[^ ^88^ ^]^ ligands for Toll‐like receptors,^[^ ^89^, ^90^ ^]^ innate immunomodulators,^[^ ^91^ ^]^ oncolytic viruses,^[^ ^92^ ^]^ etc. Our approach in the present study differs from previous ones as we promote tumor antigen release from their chaperone protein in the endosomes of cDC1 to enhance antigen release into cytosols for efficient cross‐presentation, which would overcome immune evasion mediated by persistent shuttling of tumor EV‐packaged CDC37. Moreover, our study suggested that targeting molecular cargoes shuttled via tumor EVs might promise an effective strategy to enhance anti‐tumor immune responses,^[^ ^93^ ^]^ since tumor cells could exploit EV shuttling among various inflammatory or immune infiltrates to evade host immune attack.^[^ ^72^, ^94^ ^]^

Collectively, our study highlighted the critical role of antigen cross‐presentation in anti‐tumor immunity and ICB therapeutic response. More importantly, we identified a suppressor of antigen/chaperone release that tumor cells exploit to inhibit antigen cross‐presentation of APCs, which may serve as a therapeutic target to devise novel tumor immunotherapeutic strategies. Future studies are needed to investigate feasible delivery methods for CDC37 targeting molecules for clinical application.

## 4 Experimental Section

### Patients and Tumor Specimens

Formalin‐fixed paraffin‐embedded (FFPE) tumor tissues and paired TdLN samples were obtained from 84 female patients diagnosed with TNBC, aged from 30 to 80 years, who received chemotherapy combined with anti‐PD‐1 therapy during 2018‐2023. Whole‐exome sequencing (WES) was performed on these samples, alongside CDC37 protein expression analysis using immunohistochemical (IHC) staining, and tumor‐specific CTL infiltration detection by immunofluorescence co‐staining of CD8 (Cat# 17 147, Abcam) and HLA‐A*02‐restricted MUC1‐pentamer (Cat# 208‐2A‐G, Proimmune) or GZMB (Cat# 22645‐206, Abcam). Therapeutic effects were evaluated according to the standard of Response Evaluation Criteria in Solid Tumors (RECIST).^[^ ^95^, ^96^ ^]^ Pathological complete response (pCR) was defined as no residual invasive carcinoma in any excised breast or lymph node tissue after neoadjuvant therapy detected by microscopy. Partial response (PR) was defined as at least a 30% reduction in the sum of the longest diameter of target lesions evaluated through clinical examination and imaging studies. Disease progressione (PD) was defined as at least a 20% increase in the sum of the longest diameter of target lesions detected by clinical examination and imaging studies, and stable disease (SD) was defined as neither sufficient shrinkage to qualify as PR nor sufficient increase to qualify as PD. pCR and PR were classified as treatment‐sensitive, while SD and PD were classified as treatment‐resistant.

Another cohort consisting of FFPE tumor samples from 138 TNBC patients from 2016 to 2022 was obtained for immunostaining of CDC37 expression and tumor‐specific CTL infiltration, followed by Kaplan‐Meier survival analysis and Cox regression analysis.

Fresh surgical tumor, TdLN and peripheral blood samples were collected from 15 breast cancer patients aged between 28 and 63 years for the isolation of primary tumor cells, T cells and DCs, followed by in vitro and in vivo experiments. Peripheral blood samples from healthy donors were collected for the isolation of monocyte‐derived DCs and T cells for in vitro experiments.

All human samples used in this study were collected from patients at Sun Yat‐Sen Memorial Hospital, Sun Yat‐Sen University, with informed consent. All experiments were conducted in accordance with the approvals and regulations of the internal review and ethics board at Sun Yat‐Sen Memorial Hospital.

### Cell Lines

Human breast cancer cell lines SKBR3, MDA‐MB‐468; human embryonic kidney cell line HEK293T were purchased from Cell Bank/Stem Cell Bank, Chinese Academy of Sciences. Mouse breast cancer cell line EO771 (94A001) was obtained from CH3 Biosystems. All cell lines were authenticated using short tandem repeat profiling (STR) and confirmed to be mycoplasma‐ and bacterial‐free.

The Cas9 lentivirus and guide RNA (gRNA) against CDC37 lentivirus were purchased from IGEbio and transduced into MDA‐MB‐468 cells (multiplicity of infection MOI = 1) with the addition of 8 µg ml^−1^ Polybrene (Cat# TR‐1003, Sigma) for 12–16 h, followed by a 2‐week selection period with 2 µg ml^−1^ puromycin. For transient knockdown and plasmid overexpression, Lipofectamine 3000 (Thermo Fisher Scientific, L3000075) was used to transfect siRNAs or plasmid according to the manufacturer's instructions. In brief, 1×10^5^ tumor cells were transfected with siRNAs or plasmids for 12–18 h. After 48–72 h, cells were collected for subsequent sample analysis. EO771‐OVA and Cdc37^KD^‐EO771‐OVA were generated by lentiviral transduction of EO771 using pCDH‐CMV‐MCS‐EF1‐copGFP‐T2A‐Puro vector carrying the OVAL (NCBI ID: 396 058) and pGLVH1‐GFP‐T2A‐Puro vector targeting Cdc37 (NCBI ID: 12 539). Transduction or transfection efficiencies were evaluated by the presence of GFP‐expressing cells using flow cytometry or the expression of target proteins using western blotting. Sequences of sgRNAs, siRNAs, and shRNAs are listed as follows:

CDC37‐sg1: GAACATCGACACGGCCAGTCT
CDC37‐sg2: GCAGTTCTTCACTAAGATTA
ALIX‐si1: AAGTACCTCAGTCTATATTGA
ALIX‐si2: AATCGAGACGCTCCTGAGATA
PSME2‐si1: GUGGAAGCUUUCCAGACAATT
PSME2‐si2: GGAGACUCAUGUAAUGGAUTT
CHIP‐si1: UGCCGCCACUAUCUGUGUAAUTT
CHIP‐si2: AUUACACAGAUAGUGGCGGCATT
shGFP:TTCTCCGAACGTGTCACGT
shCdc37‐1: GGAGGGCTTCAAGTATGAACT
shCdc37‐2: GGATGTACAGATGCTGCAAGA

### Animal Experiments

Female wild‐type (WT) C57BL/6 and NOD/SCIDγ‐ null (NSG) mice were purchased from Laboratory Animal Center of Sun Yat‐Sen University. OT‐I transgenic mice and Batf3 KO mice (Batf^−/−^) were purchased from the Jackson Laboratory. All mice were bred in the specific‐pathogen‐free (SPF) animal facility at the Laboratory Animal Centre of Sun Yat‐Sen University. Animal experiments were approved by the Institutional Animal Care and Use Committee of Sun Yat‐Sen University.

Orthotopic tumor models were established by inoculating 5 × 10^5^ EO771‐OVA or Cdc37^KD^‐EO771‐OVA cells into the fourth mammary fat pad of C57BL/6 mice, and 5 × 10^5^ EO771‐OVA cells into the Batf3^−/−^ mice. Tumor‐bearing mice were treated intraperitoneally (i.p.) with either control IgG2 (Cat# BE0090, Bio X Cell, clone LTF‐2) or αPD‐L1 (Cat# BE0101, Bio X Cell, clone 10F.9G2) at a dose of 5 mg kg^−1^ every three days. DDO‐5936 (Cat# 139 301, MCE) was administered i.p. at a dose of 100 mg kg^−1^ daily. Tumor growth and treatment response were monitored via caliper measurements every three days after tumor inoculation. T cells and DCs from the tumor and TdLN were isolated and purified for further analysis. Tissue was fixed in 4% paraformaldehyde (Cat# FB002, Thermo Fisher Scientific) for 24 h at 4 °C and washed three times with PBS. Fine sections (4 µm) were prepared from paraffin‐embedded tumor tissues and mounted on glass slides for immunostaining. The sizes of experimental groups were determined and approved by the regulatory authorities for animal welfare, taking into account statistical power, ethical considerations, and feasibility.^[^ ^97^, ^98^ ^]^

### Patient‐Derived Xenograft (PDX) Implantation

The primary tumor tissues of TMB^hi^CTL^lo^ breast cancer were collected from HLA‐A2^+^ patients with MUC1^+^CDC37^hi^ tumors who underwent surgical resection at Sun Yat‐Sen Memorial Hospital, Sun Yat‐Sen University, and were successfully used to establish PDX models before. All animal procedures were approved by the hospital's ethics committees and Clinical Research Committee. PDX establishment was followed previously published protocols.^[^ ^97^ ^]^ Briefly, Female NSG mice (4–5 weeks old) were engrafted with 1–2 mm^3^ tumor fragments into cleared inguinal mammary fat pads within 45–120 min of collection. Tumor growth was monitored and mice were euthanized when tumors reached ≈1500 mm^3^, or the study ended. At the end of the experiment, the tumor tissues were subjected to histology, primary‐cell isolation or stored frozen for future use. Tumor volumes were calculated as (length × width^2^)/2.

### Adoptive Cell Transfer Therapy

CD8^+^ T cells were isolated from the peripheral blood of breast cancer patients using CD8^+^ T Cell MicroBeads (Miltenyi Biotec, 130‐116‐478) and co‐cultured for 20 h with autologous tumor lysate‐pulsed DCs transduced with sgNC or sgHSP90. Once PDX tumors became palpable, 2.5 × 10^6^ CD8^+^ T cells and 0.5 × 10^6^ DCs were intravenously injected into tumor‐bearing NSG mice. In parallel, mice received intraperitoneal administration of αPD‐L1 (5 mg kg^−1^, every three days) or/and DDO‐5936 (100 mg kg^−1^, daily). Tumor growth was monitored, and tumor cells together with infiltrating DCs or lymphocytes were analyzed by flow cytometry or immunofluorescence.

### Tumor Mutational Burden (TMB) Analysis

WES was performed using the Illumina HiSeq 4000 platform (Illumina Inc., San Diego, CA). Raw sequencing data underwent a preliminary statistical analysis before being optimized using Cutadapt software, where primers, adapters, and bases with a quality score below 20 at both ends were removed, along with reads containing an N base ratio exceeding 10%. Post quality control (QC), clean data was compared with reference genomes by Sentieon, sequenced, and regenerated into BAM files. Somatic variation analysis was performed by Sentieon. Mutations annotated with Annovar were removed if they were synonymous mutations or present in dbSNP, but were retained if listed as somatic in COSMIC (version 70), and then filtered to include only those supported by at least 20 reads and with a variant allele frequency (VAF) of at least 5%.^[^ ^30^, ^99^, ^100^ ^]^ Finally, TMB was calculated as the total number of single nucleotide variants (SNVs) and indels divided by the 60 Mb of DNA sequenced.

### Immunohistochemical/ Immunofluorescence Staining

FFPE tissues from human or mouse tumors were deparaffinized and rehydrated. Antigen retrieval was performed by a pressure cooker for 3 min in EDTA buffer (pH 8.0).

For IHC, the slides were treated with 3% hydrogen peroxide (H_2_O_2_) solution for 10 min and blocked with 5% bovine serum albumin (BSA) for 30 min at room temperature. The slides were then incubated with mouse anti‐human CDC37 antibody (Cat# 66420‐1‐Ig, Proteintech, 1:100) overnight at 4 °C, washed with PBS for three times, and stained with an anti‐mouse/rabbit IHC Secondary Antibody Kit (Cat# GK500705, GTVision), following the manufacturer's instructions. The sections were counterstained with hematoxylin (Vector Laboratories) after developing with 0.02% 3,3′‐diaminobenzidine (DAB, Cat# GK500710, Gene Tech) substrate. The slides were digitalized on a BX53 biological upright microscope (Olympus). IHC images were evaluated by IHC Profiler, an ImageJ plug‐in, in a blinded manner as previously described.^[^ ^101^ ^]^ The staining intensity was scored using the following scale: 0, negative; 1, low positive; 2, positive; 3, high positive. The IHC score, which was determined by multiplying the staining intensity score and percentage of positive cells, was generated from all the grids of the slides. The average score was calculated for each sample. The theoretical limits of the scores ranged from 0 (0% cell staining) to 300 (100% cell staining at 3+ intensity). CDC37 IHC score was classified into low or high expression using the median value as the cut‐off point.^[^ ^102^ ^]^

For immunofluorescence, the slides were blocked with 5% BSA for 30 min at room temperature to avoid non‐specific binding. The samples were incubated with rabbit anti‐human GZMB antibody (Cat# 22645‐206, Abcam, 1:100), mouse anti‐human CD8 antibody (Cat#17 147, Abcam, 1:100), rabbit anti‐human CD11c antibody (Cat#52 638, Abcam, 1:100), mouse anti‐human CD1a antibody (Cat#201 337, Abcam, 1:100), mouse anti‐human CD141 antibody (Cat# 6980, Abcam, 1:100)overnight at 4 °C, followed by incubation with Alexa Fluor‐conjugated secondary antibodies (Cat# A21202, Cat# A31571, Cat# A31572, Cat# A21206, Cat# A31570, Cat# A21039, Thermo Fisher Scientific, 1:200) according to the experiment design. The cell nuclei were counterstained with Hoechst 33 342 (Cat# H21492, Thermo Fisher Scientific). Images were captured using laser scanning confocal microscopy (LSM800, Zeiss). Stained cells were counted in at least 10 fields per section and 5 sections per sample and the calculation was confirmed by two independent researchers. The count of tumor‐infiltrating CTLs was categorized into low or high infiltration based on the median value as the cut‐off point. Frozen biopsy samples of tumor tissue and TdLN were routinely cut into 4‐µm‐thick slides. The slides mounted on glass slides and fixed with 4% paraformaldehyde for 3 min at room temperature were co‐stained with MHC class I pentamer and anti‐CD8. Briefly, the slides were blocked with 20% human serum for 20 min at 4 °C before incubation with PE‐labeled HLA‐A*02‐restricted MUC1‐Pentamer (Cat#208‐2A‐G, Proimmune,1:20) for 10 min at room temperature. The slides were then washed twice with 0.01 mm PBS, pH 7.4, then stained with mouse anti‐human CD8 antibody (Cat#17 147, Abcam, 1:100) overnight at 4 °C and subsequently by Alexa Fluor 488–conjugated secondary antibody (Cat#A21202, Thermo Fisher Scientific, 1:200) for 1 h at room temperature. Hoechst 33 342 (Cat# H21492, Thermo Fisher Scientific) was then used to counterstain the nuclei. Moreover, cells for immunofluorescence were fixed with 4% paraformaldehyde for 25 min at room temperature, washed with PBS, permeabilized with or without 0.2% Triton X‐100 in PBS for 10 min, and incubated with the indicated antibodies as follows: rabbit anti‐human RAB5 antibody (Cat# 21 801, Abcam, 1:100), mouse anti‐human CDC37 antibody (Cat# 66420‐1‐Ig, Proteintech, 1:100), rabbit anti‐human HSP90 antibody (Cat# 4877, CST, 1:100), anti‐MUC1 (Cat# 4532, Immunoway, 1:100), anti‐OVA antibody (Cat# 27117‐90, Abcam, 1:100). Images were captured using laser scanning confocal microscopy (LSM800, Zeiss). Super‐resolution images were reconstructed with Nikon structured illumination microscopy (N‐SIM) by capturing spatial frequencies beyond the diffraction limit and computationally combining them via Fourier transform. Colocalization of CDC37 with RAB5, HSP90, or MUC1, and colocalization of OVA with HSP90 were analyzed using ImageJ software. Colocalization was quantified either as the percentage of colocalized area relative to total CDC37 or OVA area or using the Pearson correlation coefficient (PCC) for the indicated molecules.

### Primary Cell Isolation and Cultures

Primary cells were obtained from human or mouse blood and tissue samples as previously described.^[^ ^64^ ^]^ Briefly, tumor and TdLN tissues were thoroughly washed with PBS, cut into small pieces (1–2 mm in diameter), and enzymatically digested. Tumor tissues were digested using 5% FBS DMEM containing 1 mg mL^−1^ collagenase I (Cat# LS004197, Worthington), 1 mg mL^−1^ collagenase IV (Cat# LS004188, Worthington), and 1 mg mL^−1^ DNase (Cat# 10 104 159 001, Roche) at 37 °C for 2 h, while TdLN were digested for 0.5 h under the same conditions. The cell suspensions were sequentially filtered through 500 µm mesh, 100 and 70 µm cell strainers, and then centrifuged in a Beckman Allegra X‐15R centrifuge at 500 rpm for 20 min with 1 mL cell suspension above 5 mL 45% Percoll (Cat# 17‐0891‐01, GE Healthcare) in the middle and 5 mL 60% Percoll at the bottom in a 15‐mL tube. Primary tumor cells were collected from the cell layer in the interface above 45% Percoll and further purified by Tumor Cell Isolation Kit (Cat# 130‐108‐339, Miltenyi). Mononuclear cells were collected from the cell layer at the interface between 45% and 60% Percoll. Human CD8^+^ T cells were purified using a human CD8 MicroBeads (Cat# 130‐045‐201, Miltenyi) and mouse CD8^+^ T cells were purified using a mouse CD8 (TIL) MicroBeads (Cat# 130‐116‐478, Miltenyi). Human tumor‐infiltrating cDC1 (Lineage^−^CD11c^+^MHC‐II^+^CD141^+^), human cDC1 in TdLN (Lineage^−^CD11c^+^MHC‐II^+^CD1a^+^), mouse tumor‐infiltrating cDC1 (CD45^+^CD11c^+^MHC‐II^+^CD103^+^) were sorted from single‐cell suspensions of mononuclear cell layer according to their surface markers using flow cytometry. During sorting, viable cells were identified using the LIVE/DEAD Fixable Yellow Dead Cell Stain Kit (Thermo Fisher Scientific). The harvested cells were immediately used for further experiments.

### Human Monocyte‐Derived Dendritic Cells

A previously described method was used to generate human monocyte‐derived DCs (moDCs).^[^ ^64^ ^]^ In brief, monocytes were isolated from the peripheral blood of healthy donors and cultured in DC culture medium containing 20 ng mL^−1^ IL‐4 (Cat# 200‐04, PeproTech) and 50 ng mL^−1^ GM‐CSF (Cat# 300‐03, PeproTech) for 6 days. The cultures were replaced with fresh medium every 3 days. DCs were treated with TCM, the soluble components or EVs of TMB^high^CTL^high^ or TMB^high^CTL^low^ tumor supernatants, respectively, for 16–24 h, followed by incubation with 200 µg mL^−1^ tumor lysates or 250 µg mL^−1^ OVA protein (Cat# 9006‐59‐1, MCE) or MUC1 recombinant protein (Cat# HY‐P78740, MCE) according to the experiment design. In some cases, before being pulsed with antigen, DCs were pretreated with DDO‐5936 at the indicated concentration for 24 h to block the binding of HSP90 and CDC37, with MG132 (Cat# M8699, Sigma, 5 µm) or Lactacystin(Cat# L6785, Sigma, 2.5 µg mL^−1^) to block the cytosolic pathway, with cathepsin inhibitor leupeptin (Cat# L2884, Sigma, 100 µg mL^−1^) for 8 h to block vacuolar pathway of antigen cross‐presentation, with Eeyarestatin I (EeyI, Cat# E1286, Sigma, 3 µm) to inhibit ERAD pathway, respectively. In other cases, before cocultured with mCherry‐CDC37‐containing tumor EVs, DCs were pretreated with Cytochalasin D (Cat# C2618, Sigma, 2 µm) and Ethylisopropylamiloride (EIPA, Cat# L593754, MCE, 50 µm), Genistein (Cat#G6776, Sigma, 200 µm) and LY294002 (Cat# 440 202, Sigma, 50 µm) or Chlorpromazine (Cat# 215 921, Sigma, 10 µm) and Dynasore (Cat# D7693, Sigma, 100 µm) to inhibit macropinocytosis, caveolin‐mediated endocytosis or clathrin‐mediated endocytosis, respectively.

### Generation of BM‐DCs

The mice were humanely euthanized, and femurs and tibias were collected. Bone marrow cells were extracted from the femurs and tibias. After purification using a mouse Monocyte Isolation kit (BM) (Cat# 130‐100‐629, Miltenyi), cells were cultured in medium consisting of RPMI 1640 with 10% FBS and 20 ng mL^−1^ mouse GM‐CSF (Cat# 315‐03, Peprotech) and 10 ng mL^−1^ mouse IL‐4 (Cat# 214‐14, Peprotech) for 6 days to obtain BMDCs.

### Dendritic Cell Transfection/ Transduction

CDC37^KO^‐DCs were generated by transducing DCs with lentiCRISPR V2‐Homo‐CDC37‐sg1/2‐copGFP (NCBI ID: 11 140). CFP‐CDC37‐overexpressing DCs and GFP‐RAB5 overexpressing DCs were generated by transfection with plasmids of pcDNA3.1(+)‐Homo‐CDC37‐TagCFP (NCBI ID: 11 140) and pCDH‐CMV‐Homo‐RAB5A‐EGFP‐EF1‐puro (NCBI ID: 5868), respectively. sgRNA sequence targeting HSP90aa1 was cloned into pSpCas9(BB)‐2A‐EGFP(pX458) CRISPR/Cas9 expression vector. Briefly, DCs were plated on a 96‐well plate (round bottom) at a concentration of 1 × 10^5^ cells per well and transduced with Cas9 lentivirus and sgRNA lentivirus against CDC37 at a MOI of 20 with the addition of 8 µg mL^−1^ Polybrene (Cat# TR‐1003, Sigma) for 12–16 h. After 48 h, GFP positive cells were sorted by flow cytometry for subsequent analysis. For transient transfection, DCs were electroporated with 2 µg of pcDNA3.1(+)‐Homo‐CDC37‐TagCFP and 2 µg of pCDH‐CMV‐Homo‐RAB5A‐EGFP‐EF1‐puro plasmid, or siRNAs targeting PSME2 or CHIP, or sgRNAs targeting HSP90, using a single 150 V electrical pulse for 10 ms (ECM 830, BTX Harvard Bioscience) in Opti‐MEM. Following electroporation, the cells were incubated in complete medium for 2 days, after which cells expressing CFP, YFP, and EGFP were simultaneously sorted by flow cytometry. Sequence of sgHSP90 is listed as follows:

HSP90aa1‐sg: GACCCAAGACCAACCGATGG

### Generation of Tumor Antigen‐Specific T Cells

Human cDC1 isolated from tumor or TdLN, as well as moDCs with indicated treatment as described above were co‐cultured with autologous naïve CD8^+^ T cells (5:1) purified from peripheral blood, respectively, for 3 days. The CD69 expression of CD8^+^ T cells was measured by flow cytometry after 24 h incubation, while the expression of MUC1‐pentamer and the production of GZMB and IFN‐γ of CD8^+^T cells were measured by flow cytometry after 72 h incubation.

TiDCs isolated from mice bearing WT or Cdc37^KD^ EO771‐OVA tumors were co‐cultured with naïve CD8^+^ T cells (5:1) isolated from spleen of OT‐I mice for 3 days. Percentages of OVA‐tetramer^+^CD8^+^ T cells and CD69 expression and GZMB production in CD8^+^ T cells were measured by flow cytometry after 24–72 h incubation.

### Flow Cytometric Sorting and Analysis

Suspended cells were stained for Live/Dead Fixable Viability Dye (FVD‐eFluor780, Cat# 65‐0865‐18, eBioscience) in PBS for 30 min at 4 °C to distinguish live and dead cells. To block non‐specific binding, mouse and human cells were incubated with FcR blocking reagent for mice (Cat# 130‐092‐575, Miltenyi) and humans (Cat# 130‐059‐901, Miltenyi), respectively. For intracellular cytokine staining, cells were re‐stimulated with a Cell Stimulation Cocktail (Cat# 00‐4970‐03, eBioscience, containing PMA and ionomycin) in the presence of a protein transport inhibitor cocktail containing Brefeldin A and Monensin (Cat# 00‐4980‐03, Thermo Fisher Scientific). Cells were stained for fluorochrome‐conjugated monoclonal antibodies targeting surface markers in PBS with 2% FBS and 2 mm EDTA or Flow Cytometry staining buffer (Cat# 00‐4222‐57, Thermo Fisher Scientific). The monoclonal antibodies were as follows: anti‐human CD8 (APC, Cat# 980 904, BioLegend, 1:20), MUC1‐pentamer (PE, Cat# 208‐2A‐G, Proimmune, 1:20), anti‐human CD69 (BV421, Cat# 310 930, BioLegend 1:20), anti‐human CD11c (FITC, Cat# 337 214, BioLegend, 1:20), anti‐human CD80 (APC, Cat# 305 220, BioLegend,1:20), anti‐human CD86 (PB, Cat# 305 423, BioLegend, 1:20); anti‐mouse H‐2K^b^ bound to SIINFEKL (APC, Cat# 141 606, BioLegend, 1:20), anti‐mouse CD8 (APC, Cat# 162 306, BioLegend, 1:20), OVA‐tetramer (PE, Cat# TS‐5001‐1C, MBL, 1:20), anti‐mouse CD11b (APC, Cat# 101 205, BioLegend, 1:20), anti‐mouse F4/80 (FITC, Cat# 123 107), anti‐mouse CD45 (BV650, Cat# 103 151, BioLegend, 1:20), anti‐mouse CD11c (BV421, Cat# 117 329, BioLegend, 1:20), anti‐mouse I‐A/I‐E (FITC, Cat# 107 605, BioLegend, 1:20), anti‐mouse CD3 (Pacific Blue, Cat# 100 333, BioLegend, 1:20), anti‐mouse CD19 (APC, Cat# 152 409, BioLegend, 1:20). Antibodies were incubated for 30 min at 4 °C, followed by cell fixation and permeabilization using IC fixation buffer (Cat# FB001, Thermo Fisher Scientific) and permeabilization buffer (Cat# 00‐8333‐56, Invitrogen). Then, cells were stained with anti‐human IFN‐γ (PE, Cat# 383 304, BioLegend, 1:20), anti‐human/mouse GZMB (Pacific Blue, Cat# 372 218, BioLegend, 1:20), anti‐human/mouse/ratαSMA (Cat# MAB1420‐SP, 0.25 ug/10^6^ cells), in 1×permeabilization buffer at room temperature for 1 h according to the manufacturer's instructions. For some experiments, Propidium Iodide (Cat# 00‐6990‐50, Thermo Fisher Scientific) was used to detect the cell death. The samples were analyzed by the Beckman CytoFLEX Flow cytometer. Data were analyzed by FlowJo software.

### Cytotoxicity Assays

The cytotoxicity assay for CD8^+^ T cells was conducted based on previously reported.^[^ ^64^ ^]^ In brief, CD8^+^ T cells primed by DCs were co‐cultured with CellTracker Deep Red (CTDR)‐labeled target cells (5 µm, Cat# C34565, Thermo Fisher) at a ratio of 10:1 in DMEM medium containing 10% FBS and 25 U mL^−1^ IL‐2 (Cat# 200‐02, PeproTech) at 37 °C with 5% CO_2_. After 12 to 18 h of incubation, both adherent and non‐adherent cells were harvested, centrifuged, resuspended in 500 µL PBS buffer, and stained with Propidium Iodide (1:50 final dilution, Cat# 421 301, BioLegend), followed by detection by flow cytometry (Beckman CytoFLEX flow cytometer). Data were analyzed by FlowJo software.

### Antigen Translocation From Endosome to Cytoplasm—β‐Lactamase Experiments

DCs were loaded with 1 mm of the CCF4‐probe (Cat# K1029, Thermo Fisher Scientific) sensitive to FRET measurements during 1 h. Cells were then washed and incubated at 37 °C with 2 mg mL^−1^ of purified β‐lactamase (Cat# ENZ‐351, Prospec) for 120 min. The fluorescence intensities of cleaved and uncleaved CCF4 in gated live single cells were detected with the 405 nm excitation laser and 450 and 535 nm emission filters, respectively, by flow cytometry. Histograms represent the fluorescence intensity ratio between 450 and 535 nm emission filters. Additionally, β‐lactamase export to cytosol was analyzed by microscopy. DCs were incubated with CCF4 cytosolic probe and loaded with 2 mg mL^−1^ of β‐lactamase for 120 min at 37 °C. After fixation by 4% paraformaldehyde, DCs were imaged by LSM880 confocal microscope. The ratio of 450 to 535 nm detected by flow cytometry, or the shift from green to blue fluorescence observed under a fluorescent microscope, represents the efficiency of antigen export into the cytosol.

### Cytochrome‐C (CytC) Experiments

DCs were incubated with 9 mg mL^−1^ cytC (Cat# 9007‐43‐6, Sigma) in the presence of 10 mm NH4Cl in complete DMEM for 20 h at 37 °C. The cells were then harvested, stained with fluorochrome‐labeled Annexin V and PI using an apoptosis detection kit (Cat# 640 932, BioLegend) as described previously,^[^ ^41^ ^]^ and analyzed by flow cytometry. Early apoptotic DCs (AnnexinV^+^PI^−^) were analyzed to assess the efficiency of antigen export into the cytosol.

### Antigen Degradation Assay

Amine‐modified polystyrene beads (Cat# 9003‐53‐6, Polysciences) were activated with 8% glutaraldehyde for 4 h at room temperature, followed by conjugation with 0.5 mg mL^−1^ OVA (Cat# 9006‐59‐1, MCE) overnight at 4 °C. After quenching with 0.5 m glycine in PBS, the beads were used in phagocytosis assays. DCs were incubated with bead‐bound OVA (bbOVA) for 10 min to allow phagocytosis, then washed with cold PBS and subsequently incubated at 37 °C for 2 h to allow degradation of internalized OVA in phagosomes (T = 2 h), or stained immediately as the T = 0 time point. External bbOVA, which was stained with anti‐OVA antibody (Cat# 27117‐9, Abcam) and an Alexa Fluor 488‐conjugated secondary antibody (Alexa‐488, Cat# A21206, Thermo Fisher Scientific), would be excluded in the later analysis. Subsequently, the cells were disrupted using syringes, centrifuged, and the intact OVA on phagosomal bbOVA was stained with anti‐OVA antibody, followed by labeling with an Alexa Fluor 647‐conjugated secondary antibody (Alexa‐647, Cat# A32795, Thermo Fisher Scientific). The Alexa‐488^−^ bb‐OVA was gated for further analysis and only the mean fluorescence intensity (MFI) of Alexa‐647 in this gate was analyzed to represent the intact OVA on beads which were not degraded in phagosomes.

### Antigen Uptake Assay

DCs were exposed to 3 µm latex beads (Cat# S37223, Thermo Fisher Scientific) covalently coupled with Alexa‐488‐labeled OVA using glutaraldehyde cross‐linking. This exposure occurred for 10 min at 37 °C at indicated cells‐to‐beads ratio. Subsequently, the cells underwent three washes with a serum gradient at 150 g (4 °C), followed by a chase period at 37 °C. Following hypoosmotic lysis and fixation, the phagosomes consisting of Alexa‐488 fluorescence were measured using flow cytometry. The percentage of Alexa‐488 positive cells among live cells represents uptake of OVA‐beads by DCs.

### Extracellular Vesicle (EV) Isolation and Characterization

TMB^hi^CTL^lo^ or TMB^hi^CTL^hi^ tumor tissues were cut into 1–2 mm^3^ fragments and digested for 30 min at 37 °C in RPMI‐1640 medium (Sigma Aldrich) containing DNase I (40 U ml^−1^, Roche). The tissue suspension was filtered through a 70‐µm filter and centrifuged sequentially at 300 g for 20 min, 3000 g for 20 min, and 10000 g for 60 min at 4 °C to remove cells, debris, and large vesicles. The clarified supernatant was filtered through a 0.22‐µm filter and subjected to ultracentrifugation at 100000 g for 90 min at 4 °C to collect EVs. The EV pellet was washed in PBS, centrifuged again at 100000 g for 90 min at 4 °C. The EV morphology was assessed by electron microscopy after negative staining with 1% uranyl acetate. Particle size distribution was measured using a ZetaView instrument. EV‐specific markers were confirmed by immunoblotting using antibodies against Alix, CD63, and CD81.

Cell culture medium of MDA‐MB‐468 without serum was collected and subjected to sequential centrifugation steps at 3,00 g for 10 min at 4 °C, 2000 g for 10 min at 4 °C and 10000 g for 30 min at 4 °C to remove cells and debris. The resulting supernatant was passed through a 0.22‐µm filter, followed by ultracentrifugation at 100000 g for 90 min at 4 °C to pellet EVs and finally resuspended in 30 µL PBS.

### Endosome and Phagosome Purification

For phagosome purification, DCs were cultured in a CO_2_‐independent medium without fetal bovine serum (FBS) and incubated with 3 µm magnetic beads at 18 °C for 20 min, followed by a transfer to 37 °C for another 15 min. Then, DCs were centrifuged at 4 °C with a force of 300 g for 4 min, which was repeated three times. Subsequently, the cells were resuspended in a homogenization buffer (PBS with 8% sucrose, 3 mm imidazole, 2 mm DTT, 5 mg ml^−1^ DNase, and protease inhibitor cocktail). The cell membrane structure was disrupted by 30 repetitive aspirations of the cell suspension utilizing a 22G syringe needle, achieving a disruption ratio greater than 70% using trypan blue (Cat# T8154, Sigma) staining. The lysed suspension was transferred to an EP tube and placed on a magnetic rack. After a 5‐min adsorption on ice, the supernatant was removed to obtain phagosomes containing magnetic beads, which were subsequently washed three times with pre‐cooled washing solution (10 mm HEPES, 110 mm KCl, 10 mm NaCl, 5 mm MgCl_2_, and 2 mm DTT in H_2_O), and centrifuged at 4 °C at 13,400 g for 15 min.

For endosome purification, DCs were incubated with 100 nm latex beads (Cat# S37204, Thermo Fisher Scientific) at 37 °C for 15 min and immediately washed with ice‐cold PBS. The cells were centrifuged at 4 °C at 500 g for 5 min and resuspended in a homogenization buffer (Sucrose 250 mm, Imidazole 3 mm, EDTA 1 mm, Cycloheximide 0.03 mm and protease inhibitor cocktail, HB). The cell suspension was repetitively aspirated utilizing a 27G syringe needle, until 90% of the cells were disrupted, while the nuclei remained intact. Afterward, the cell supernatant was collected by centrifugation at 500 g for 5 min at 4 °C. The homogenized sample was mixed with an equal volume of 62% sucrose solution to obtain a final sucrose concentration of 40%. Later, the sample was overlaid with 35%, 25%, and 10% sucrose solution in order and centrifuged at 100000 g for 75 min at 4 °C. The organelles containing latex beads were collected from the interface between the 25% and 10% sucrose solution layers, diluted with ice‐cold 3 mm imidazole (pH 7.4) at a 1:4 ratio. Then the tube was filled up to 12 mL with ice‐cold HB and centrifuged at 100000 g for 75 min at 4 °C to isolate early endosomes.

### Co‐Immunoprecipitation

DC endosomes were isolated as described above and subsequently lysed in Pierce IP lysis buffer (Cat# 87 787, Thermo Fisher Scientific) containing protease inhibitor (Cat# 78 446, Thermo Fisher Scientific). After centrifugation at 14 000 g for 15 min at 4 °C, the supernatant was collected and incubated with Pierce Protein A/G Magnetic Beads (Cat# 88 802, Thermo Fisher Scientific) and rabbit anti‐human CDC37 (Cat# 10218‐1‐AP8, Proteintech, 1:50), rabbit anti‐human HSP90 antibody (Cat#13171‐1‐AP, Proteintech, 1:50) or rabbit anti‐His antibody (Cat# 9367, Cell Signaling Technology, 1:100) for 1 h at room temperature. Dynabeads‐antigen‐antibody complex was collected after centrifugation at 14 000 rpm for 5 s and washed with IP lysis buffer three times, followed by analyzing the co‐immunoprecipitates by MS or immunoblotting.

### LC–MS/MS Analysis

Online chromatography of tryptic peptides, derived from co‐immunoprecipitation proteins, was performed using the Easy nLC 1200 system (Thermo Fisher Scientific). Lyophilized peptide fractions were reconstituted in ddH_2_O with 0.1% formic acid, and 2 µL aliquots were loaded onto a nanoViper C18 trap column (Acclaim PepMap 100, 75 µm × 2 cm). Trapping and desalting were performed using 20 µL of 100% solvent A (0.1% formic acid). Peptides were eluted over 120 min using a gradient of 5–38% solvent B (80% acetonitrile, 0.1% formic acid) on a PepMap RSLC analytical column (75 µm × 25 cm C18, 2 µm, 100 Å). Tandem mass spectrometry (MS/MS) data were acquired using a Thermo Fisher Fusion mass spectrometer equipped with a NanoFlex ion source. Data collection was carried out with an ion spray voltage of 2.2 kV and an interface heater temperature of 250 °C. Full MS scans ranged from 350 to 1250 m/z with a resolution of 60000 and a maximum injection time of 100 ms. MS2 scans were performed on precursor ions with charge states of 2–5 using higher‐energy collision dissociation (HCD) at a normalized collision energy of 28. Rapid acquisition was carried out in the ion trap with an AGC target of 8000 and a maximum injection time of 50 ms. Dynamic exclusion was set to 60 s. The MS data were deposited to the ProteomeXchange Consortium (PXD069576).

### Western Blotting

Cells were lysed using RIPA buffer (Cat# 89 900, Thermo Fisher Scientific) containing protease inhibitor (Cat# 78 446, Thermo Fisher Scientific). Protein concentration was determined using the BCA detection kit (Cat# 23 225, Thermo Fisher Scientific). Protein samples were loaded on SDS‐PAGE gels for electrophoresis and transferred to PVDF membranes (Cat# IPVH00010, Millipore). Membranes were blocked in 5% BSA for 30 min at room temperature and then incubated at 4 °C with the following primary antibodies: rabbit anti‐human HSP90 (Cat# 4877, CST, 1:1000), mouse anti‐human CDC37 (Cat# 66 420, Proteintech, 1:1000), mouse anti‐human MUC‐1 (Cat# 4532, Immunoway, 1:1000), rabbit anti‐human Alix (Cat# 12422‐1‐AP, Proteintech, 1:1000), mouse anti‐human CD81 (Cat# 66866‐1‐Ig, Proteintech, 1:1000), rabbit anti‐human CD63 (Cat# 5702, Abcam, 1:1000), rabbit anti‐human EEA1 (Cat# 4245, Abcam, 1:1000), rabbit anti‐human Na/K ATPase (Cat# 1845Y, Abcam, 1:1000), rabbit anti‐human PSME2 (Cat#ab183727, Abcam, 1:1000), mouse anti‐human CHIP (Cat# sc‐133066, Santa Cruz, 1:500), mouse anti‐human GAPDH (Cat# 60 004, Proteintech, 1:10 000). Membranes were washed three times and then incubated with HRP‐conjugated anti‐rabbit antibody (Cat# 7074, Cell Signaling Technology, 1:3000) or HRP‐conjugated anti‐mouse antibody (Cat# 7076, Cell Signaling Technology, 1:3000) for 1 h at room temperature. Signals on the membranes were enhanced by chemiluminescence (ECL, Cat# 34 580, Thermo Fisher Scientific) and images were captured by the Bio‐Rad GelDoc Go system. In some experiments to ensure consistent loading of EV protein samples across different groups, the WB membrane was stained using Ponceau S solution (Cat# 6226‐79‐5, R&D) for 5–10 min at room temperature and washed with distilled water until the protein bands were clearly visible. After imaging the stained membranes, they were destained with distilled water and subjected to blocking and antibody incubation as described above.

### qRT‐PCR Analysis

Total RNA was extracted using TRIzol (Cat# 15 596 018, Thermo Fisher Scientific). RNA concentration and quality were assessed by the NanoDrop 2000 spectrophotometer. Subsequently, total RNA was reverse transcribed into cDNA with PrimeScript RT reagent Kit (Cat# RR047A, Takara), followed by quantitative RT‐PCR (qRT‐PCR) using TB Green Premix Ex Taq II kit (Cat# CN830S, Takara) according to the manufacturer's instruction. Data were collected and analyzed with a LightCycler 480 instrument (Roche). All primer sequences are listed in the Table S4 (Supporting Information), key resource table.

### Live Cell Imaging

To monitor the dynamic process of CDC37 internalization into DC endosomes, 1 × 10^5^ DCs transfected with GFP‐RAB5 were seeded per dish and treated with mCherry‐CDC37‐overexpressing SKBR3 cell‐derived EVs at 37 °C under light‐avoiding conditions. After 15 min of incubation, the cells were observed under the LSM880 confocal microscope. A time series was recorded at 180‐s intervals, capturing 8 time points to monitor the changes and co‐localization of GFP and mCherry fluorescence signals within the cells, then acquired images at 1 and 2 h to observe the intracellular fluorescence signals of GFP and mCherry.

To monitor the dynamic process of OVA binding to and dissociating from HSP90, 1 × 10^5^ DCs transfected with GFP‐HSP90 were seeded under the same conditions and treated with EVs derived from non‐fluorescent MDA‐MB‐468 tumor cells, followed by treatment with DDO‐5936 for 24 h and 5 µm MG132 for 8 h. Subsequently, DCs were pulsed with mCherry‐OVA antigen in the presence of MG132 and incubated in the dark for 15 min. After three washes with pre‐cooled PBS, cells were observed under the LSM880 confocal microscope at room temperature. A time series was recorded with a 90‐s interval, capturing 6 time points to track the binding and subsequent dissociation of GFP‐HSP90 and mCherry‐OVA fluorescence signals within the cells.

### Fluorescence Resonance Energy Transfer (FRET)

DCs transfected with HSP90‐YFP and RAB5‐GFP plasmids were exposed to tumor‐derived EVs containing CDC37‐CFP, with or without DDO‐5936 treatment. For CFP–YFP FRET imaging, three scans were performed sequentially. The first scan captured emission in the 475–535 nm range (CFP) and >520 nm range (FRET) under 405 nm excitation, followed by the second scan in the 500–520 nm range (GFP) under 488 nm excitation. The third scan used 514 nm excitation, and >520 nm YFP emission was measured. Images were acquired using a Zeiss LSM880 laser‐scanning confocal microscope. Regions of interest (ROI) were defined based on RAB5‐positive areas to analyze FRET between HSP90‐YFP and CDC37‐CFP within the endosomes. FRET efficiency (FRET_eff_) was calculated using the formula: FRET_eff_ = FRET_corr_/(FRET_corr_ + donor) × 100%, where FRET_corr_ represents the corrected pixel intensity, and the donor corresponds to the donor channel intensity.

### Nikon SIM Super‐Resolution Imaging

The SIM images were acquired using a Nikon N‐SIM system. The blue imaging channel for DAPI with emission bandwidth at 420–495 nm upon excitation at 405 nm, the green imaging channel for GFP with emission bandwidth at 500–550 nm upon excitation at 488 nm, the red imaging channel for mCherry with emission bandwidth at 570–640 nm upon excitation at 561 nm were utilized. The imaging data analysis and thermal map construction were performed via analysis with ImageJ.

### Single‐Cell RNA Sequencing (scRNA‐seq) and Bioinformatics Analysis

Single‐cell RNA sequencing (scRNA‐seq) data from treatment‐naïve breast cancer were obtained from GSE161529,^[^ ^48^ ^]^ and data from PD1‐treated breast cancer were obtained from the publicly available database (http://biokey.lambrechtslab.org/) following the data access instructions described in the original article.^[^ ^65^ ^]^ Both raw and processed data were downloaded from the repositories specified in the original publications. All analyses were performed using 10×Genomics CellRanger (v3.0.2) pipeline. Subsequent single‐cell RNA‐seq analysis was performed in R package “Seurat” (v5.0.1). Raw data were filtered according to the methodological standards described in the original paper.^[^ ^48^ ^]^ Data were normalized and scaled within each sample, and canonical correlation analysis was performed to integrate data across samples. Dimensionality reduction was performed with principal component analysis (PCA), and significant principal components were used for graph‐based clustering with the Louvain algorithm. Uniform manifold approximation and projection (UMAP) was applied for visualization. Cluster annotation was guided by canonical marker gene expression and cross‐validated with the cell type assignments reported in the original datasets. Expression of CDC37 was assessed across major cell types using the “FindMarkers” function in Seurat. Differential expression analysis was performed within annotated clusters.

### Statistical Analysis

Statistical analyses were performed using GraphPad Prism 9 (GraphPad) and SPSS Statistics (v.26) software. All continuous data were presented as means ± standard deviation (SD). For comparison between two groups, *P* values were calculated using two‐tailed Student's t tests for continuous variables and chi‐square test for categorical variables. For comparison among multiple groups, *P* values were calculated using two‐tailed one‐way ANOVA, with Dunnett's test for control comparisons and Tukey's test for pairwise comparisons. Kaplan‐Meier survival curves were plotted for survival analysis, and the log‐rank (Mantel‐Cox) test was used to compare survival curves. X‐tile statistical software was used to determine an optimal cutoff point in TCGA database analysis by a minimal *P* value approach as previously reported.^[^ ^103^ ^]^ Multivariable Cox regression analysis was used to identify the independent prognostic factors. Spearman's correlation was performed to assess the relationship between TMB and GZMB^+^CD8^+^ T cells, while Pearson's correlation was applied to analyze the relationship between CDC37 and MUC1‐pentamer^+^CD8^+^ T cells or GZMB^+^CD8^+^ T cells. Bars in the experiments indicate SD as mentioned in figure legends based on a minimum of three independent experiments. CDC37 expression in tumor cells from TNBC patients classified as responders or non‐responders to anti‐PD‐1 therapy based on scRNA‐seq data was evaluated using two‐sided Wilcoxon rank‐sum test. CDC37 expression across multiple cell types from TNBC patients, based on scRNA‐seq data, was compared to tumor epithelial cells using the Kruskal–Wallis H test, followed by pairwise two‐tailed Wilcoxon rank‐sum tests with Benjamini–Hochberg (BH) correction for multiple comparisons. A *P* value of less than 0.05 was considered statistically significant.

## Conflict of Interest

The authors declare no conflict of interest.

## Author Contributions

R.W., Y.Z., and X.M. contributed equally to this work. R.W., D.H., and E.S. performed conceptualization and wrote the original draft. R.W., D.H., X.M., Y.Z., Y.X., and Q.O., performed methodology. R.W., X.M., Y.Z., and N.Z. performed investigation. R.W., Y.Z., X.M., N.M., and H.C. performed visualization. D.H. and E.S. performed funding acquisition, project administration, and supervision.

## Supporting information



Supporting Information

Supplemental Table 2

Supplemental Video 1‐3

## Data Availability

The data that support the findings of this study are available from the corresponding authors upon reasonable request. The data deposited and made public are compliant with the regulations of the Ministry of Science and Technology of the People's Republic of China. All data are available in the main text or the Supplementary materials. The data that support the findings of this study are openly available in [Genome Sequence Archive] at [https://www.ngdc.cncb.ac.cn/gsa/], reference number [HRA013712].
