# Supplementary material for: Tumor‐Derived CDC37 Inhibits Antigen Cross‐Presentation in Dendritic Cells and Impairs Anti‐Tumor Immunity in Breast Cancer
Source: Adv Sci (Weinh). 2025 Nov 10;13(5):e06518. doi: 10.1002/advs.202506518 (PMC12849997; doi:10.1002/advs.202506518)

**Supporting Information**

Supporting Information is available from the Wiley Online Library or from the author.

Tumor-derived CDC37, shuttled via extracellular vesicles (EVs), stabilizes the antigen/HSP90 complex within endosomes of dendritic cells (DCs) and impedes antigen release into the cytosol, resulting in defective antigen cross-presentation by DCs and diminished generation of tumor-specific cytotoxic T lymphocytes. Targeting CDC37 restores anti-tumor immunity and reverses resistance to immune checkpoint blockade.

Tumor-derived CDC37 inhibits antigen cross-presentation in dendritic cells and impairs anti-tumor immunity in breast cancer

*Ruxin Wang^1,3,4^, Yunyi Zhang^1,3,4^, Xiangyu Meng^1,3,4^, Jianli Zhao^1,3^, Yue Xing^1,3^, Qian Ouyang^1,3^, Ning Zhang^1,3^, Huiping Chen^1,3^, Nanyan Miao^1,3^, Erwei Song^1,2,3,*^, Di Huang^1,3,5,*^*


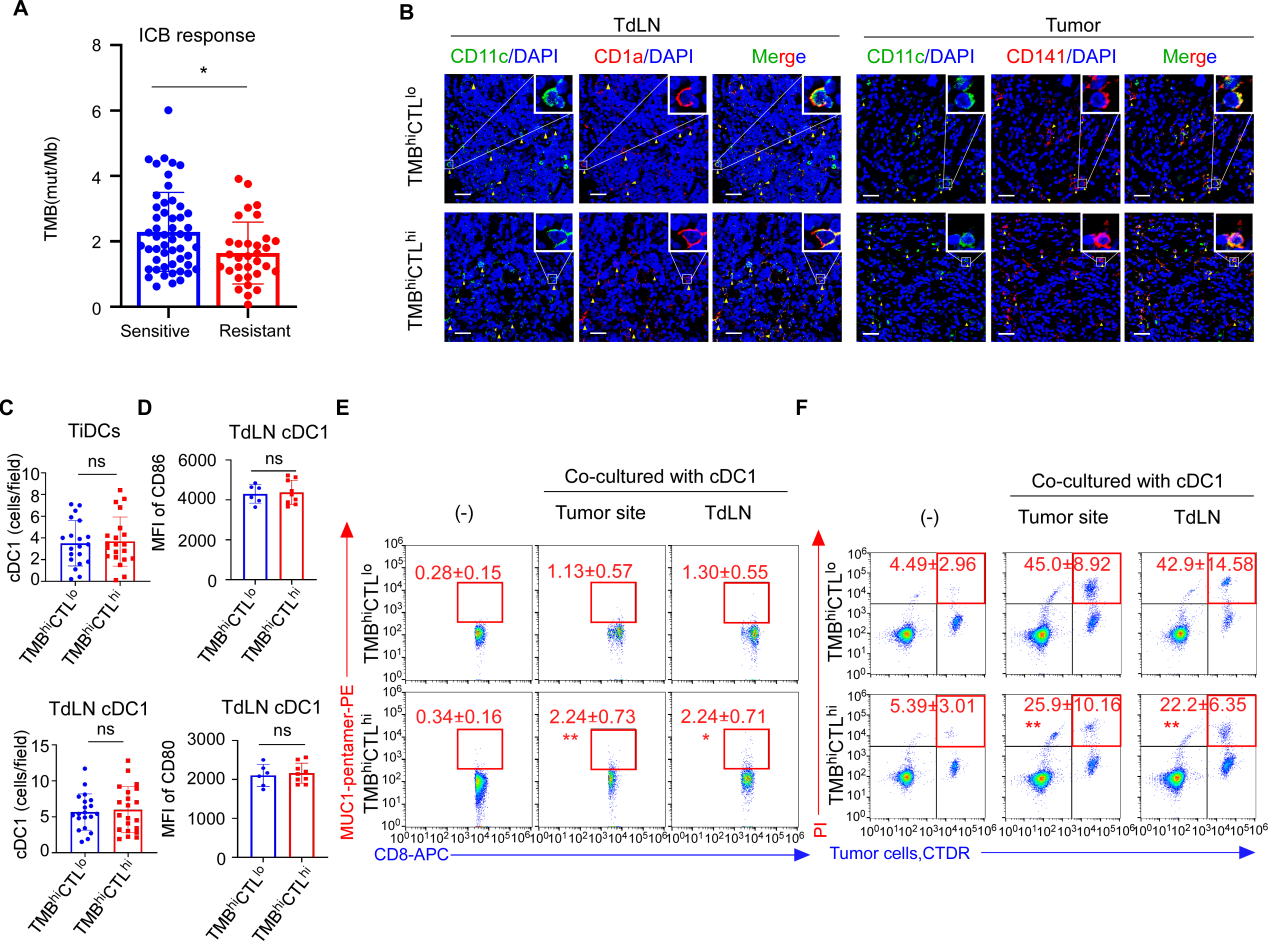


**Figure S1: Poor CTL infiltration in tumors with high TMB was not attributed to PD-L1 status in the tumor or the quantity of mature cDC1.**

(**A**). Quantification of TMB values in breast cancer patients that were sensitive (n = 52) or resistant (n = 32) to ICB therapy.

(**B,C**). Representative images (**B**) and quantification (**C**) of cDC1 (indicated by arrows) in TdLN (denoted by CD11c^+^CD1a^+^ co-staining) or in tumor site (denoted by CD11c^+^CD141^+^ co-staining) of breast cancer patients with TMB^hi^CTL^hi^ tumors (n= 21) or the ones with TMB^hi^CTL^lo^ tumors (n= 20). Asterisks denote the area of higher magnification images shown at the top right corner. Scale bar, 50 μm.

(**D**). Quantification of CD86 and CD80 in cDC1 in tumor and TdLN of breast cancer patients with TMB^hi^CTL^hi^ (n = 9) and TMB^hi^CTL^lo^ (n = 6), determined by flow cytometry. MFI, mean fluoresent intensity.

(**E,F**). Naive CD8^+^ T cells were cultured alone (-) or primed by cDC1 isolated from tumor site or TdLN of TMB^hi^CTL^hi^ (n= 9) or TMB^hi^CTL^lo^ (n= 6) breast cancer patients. (**E**) Representative flow cytometric plots and quantification of MUC1-pentamer staining in the *in vitro* primed CD8^+^ T cells. (**F**) The death of tumor cells pre-stained with CellTracker Deep Red (CTDR) induced by *in vitro* primed CTLs was examined by propidium iodide (PI) uptake through flow cytometry. Numbers in the plots indicate percentages of PI^+^ tumor cells.

Results are mean ± s.d. of independent experiments producing similar results. **P* < 0.05, ***P* < 0.01, ns, no significance, compared with ICB sensitive group (A), or TMB^hi^CTL^lo^ group (C-F), were determined by two-tailed Student’s t test (A, C-F).


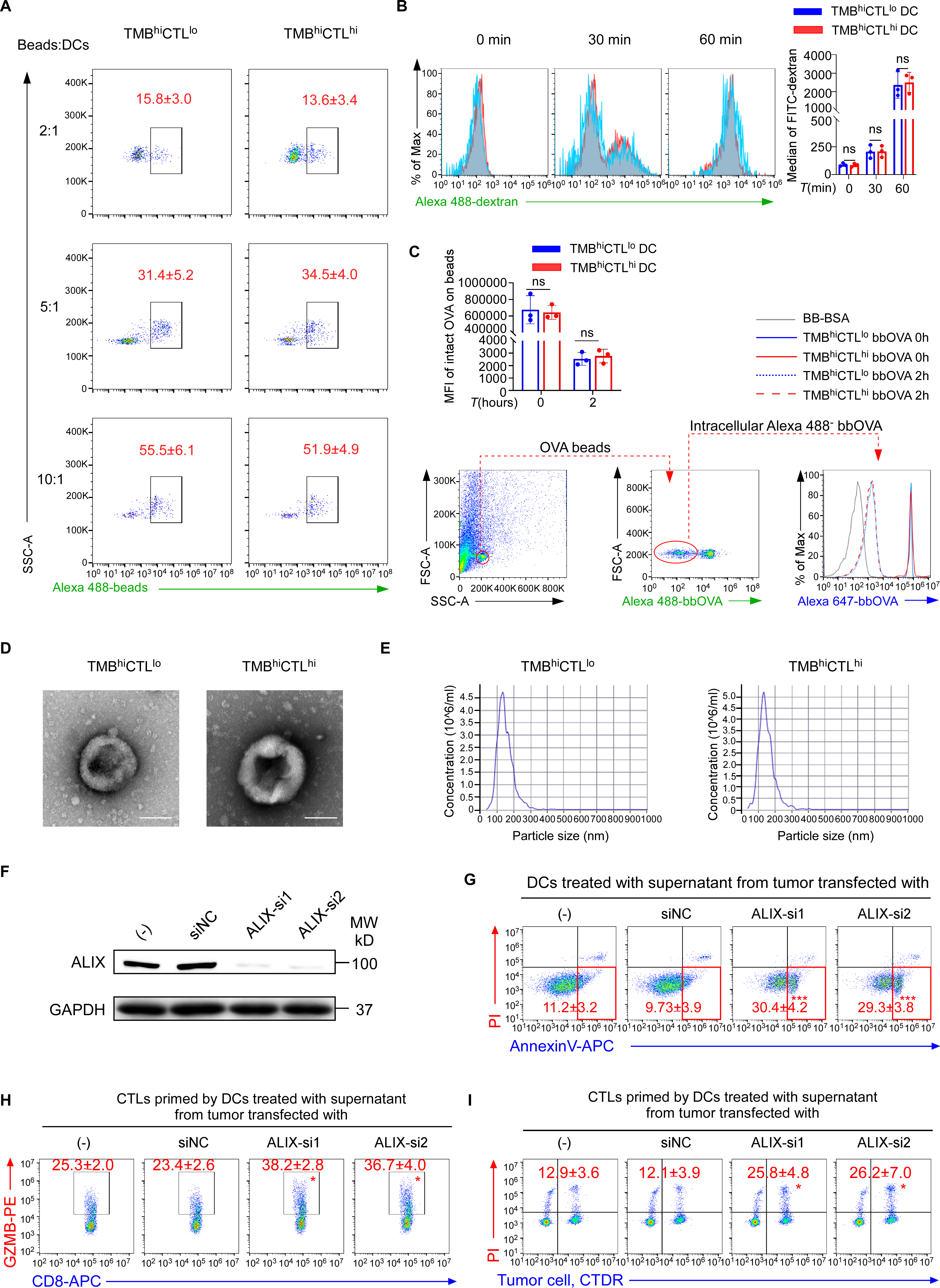


**Figure S2: The function of antigen capturing and phagosomal degradation were not influenced in TiDCs from TMB^hi^CTL^lo^ tumors.**

(**A**). Phagocytosis assay. TiDCs from TMB^hi^CTL^lo^ (n = 3) and TMB^hi^CTL^hi^ (n = 3) breast tumor were incubated for 10 min with various amounts of Alexa-488-labeled latex beads, respectively, and analyzed by flow cytometry in the presence of trypan blue to quench the fluorescence of extracellular beads. Representative plots and quantification of the percentages of phagocytic cells.

(**B**). Endocytosis assay. TiDCs from TMB^hi^CTL^lo^ (blue, n = 3) and TMB^hi^CTL^hi^ (red, n = 3) breast tumor were incubated for 0min to 60min with Alexa-488-labeled dextran, respectively, and analyzed by flow cytometry at the indicated time after incubation. Representative plots and quantification of median of fluoresent intensity of Alexa-488-labeled dextran internalized by DCs.

(**C**). TiDCs were incubated with bead bound OVA (bbOVA) for 10 min. 0 hr or 2 hrs after the incubation, the extracellular bbOVA were stained with Alexa-488 fluorescence, followed by lysis of phagosomes. Then the intact OVA in phagosomes was stained with Alexa-647 fluorescence and measured by FACS. Representative plots and quantification of Alexa-647 signal in Alexa-488^-^TiDCs of TMB^hi^CTL^lo^ (n=3) and TMB^hi^CTL^hi^ (n=3) tumors. MFI, mean fluoresent intensity.

(**D**). The morphology of sEVs from TMB^hi^CTL^lo^ and TMB^hi^CTL^hi^ breast cancer, detected with transmission electron microscopy (TEM). Scale bar = 100 nm.

(**E**). Size distribution of EVs from TMB^hi^CTL^lo^ and TMB^hi^CTL^hi^ breast cancer, detected through nanoparticle tracking analysis (NTA).

(**F-I**). DCs were treated by supernatant from MDA-MB-468 transfected with empty siRNA (siNC) or siRNAs targeting ALIX (ALIX-si1, ALIX-si2), and co-cultured with autologous naive CD8^+^ T cells. (**F**). Representative images of ALIX expression in MDA-MB-468, are shown by western blotting (n = 3 independent experiments). (**G**) Representative flow cytometric plots and quantification of AnnexinV^+^PI^-^ DCs after exogenous cytC treatment. (**H**) Representative flow cytometric plots and quantification of the percentages of GZMB^+^CD8^+^T cells primed by indicated DCs. **(I)** Tumor cell death induced by CTLs primed by indicated DCs was examined by PI uptake through flow cytometry and quantified. Numbers in the plots indicate percentages of PI+ tumor cells.

Results are mean ± s.d. of independent experiments producing similar results. ns, not significant, were determined by two-tailed Student’s t test (**A,B,C**). **P* < 0.05, ****P* < 0.001，compared with DC treated with MDA-MB-468 tumor EVs (**G,H,I**), were determined by two-tailed one-way ANOVA with Dunnett’s multiple-comparisons test (**G,H,I**).


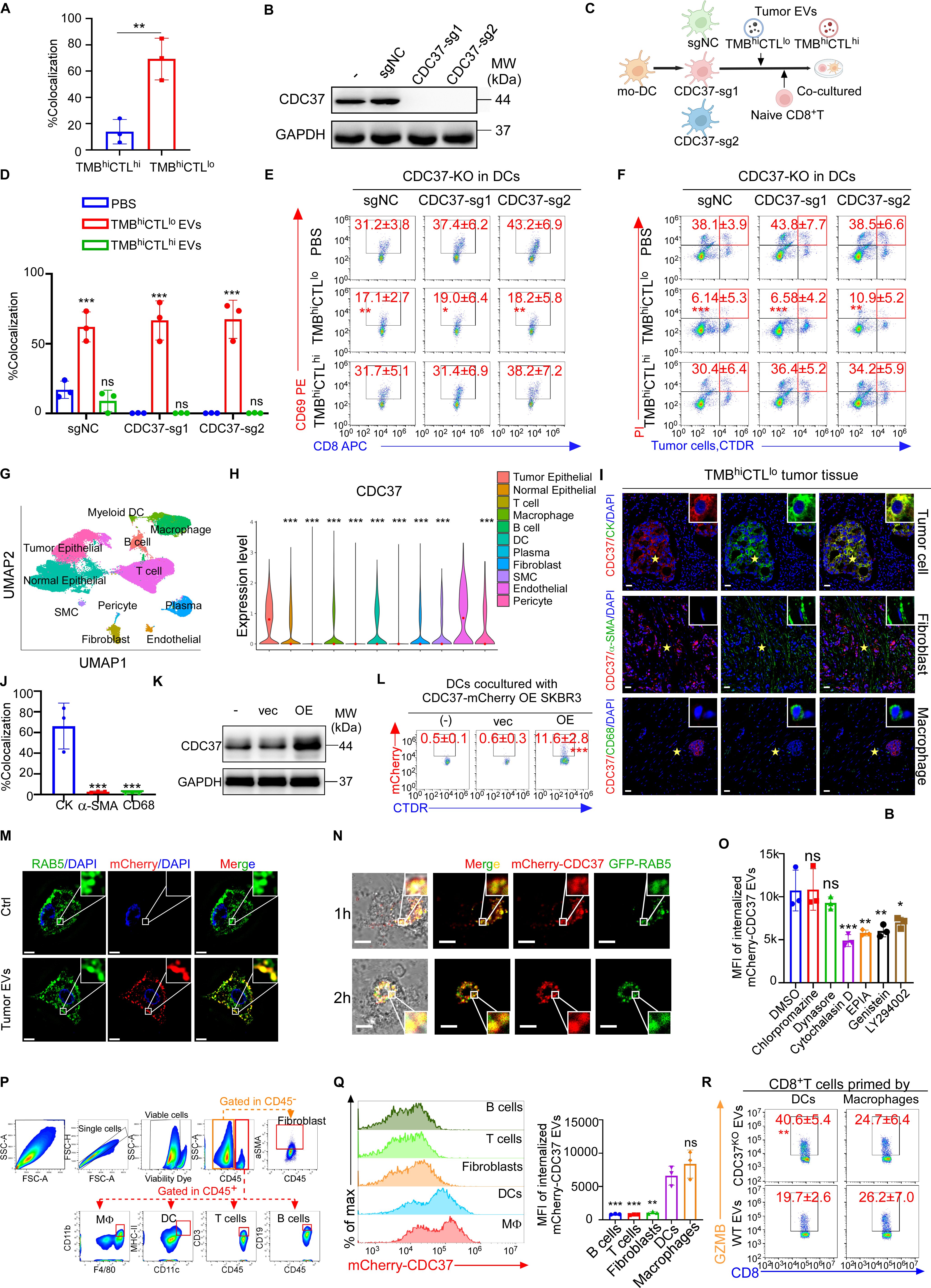


**Figure S3: Tumor EV-packaged CDC37 was shuttled to DC endosomes to suppress antigen cross-presentation.**

(**A**). Quantification of the area ratio of CDC37 colocalized with RAB5 relative to the total CDC37 in TiDCs from TMB^hi^CTL^lo^ (n = 3) or TMB^hi^CTL^hi^ (n = 3) breast tumor.

(**B-F**). DCs were transduced with empty sgRNA (sgNC), sgRNAs targeting CDC37 (CDC37-sg1, CDC37-sg2), pulsed with tumor lysates, treated with EVs from TMB^hi^CTL^lo^ (n = 3) and TMB^hi^CTL^hi^ (n = 3) breast tumor, respectively, and co-cultured with autologous naive CD8^+^ T cells. (**B**) Representative images of CDC37 expression in DCs, are shown by western blotting (n = 3 independent experiments). (**C**) Experimental schematic. (**D**) Quantification of the area ratio of CDC37 colocalized with RAB5 relative to the total CDC37 in indicated DCs. (**E**) Representative flow cytometric plots and quantification of the percentages of CD69^+^CD8^+^ T cells. (**F**) Tumor cell death induced by *in vitro* primed CTLs was examined by PI uptake through flow cytometry and quantified. Numbers in the plots indicate percentages of PI^+^ tumor cells.

(**G,H**) scRNA-seq profiles of total tissue cells from TNBC patients (n = 8), based on publicly available single-cell RNA-sequencing (scRNA-seq) data from breast cancer cohorts. Processed data were obtained from B. Pal etc., 2021,*Embo*.^[48]^.(**G**) UMAP embedding of the integrated dataset, with each dot representing a single cell and colors indicating annotated cell types. (**H**) Violin plots of CDC37 expression across the cell types identified in (**G**).

(**I,J**). Representative immunofluorescence images (**I**) and quantification (**J**) of CDC37 and CK/a-SMA/CD68 co-staining in the serial sections of cancer samples from TMB^hi^CTL^lo^ patients (n=3). Asterisks denote the area of higher magnification images shown at the top right corner. Scale bar, 50 μm.

(**K,L**). DCs pre-stained with CTDR were co-cultured with mCherry-CDC37 overexpressing (OE) SKBR3 tumor cells for 16hr. (**K**) Representative images of CDC37 expression in SKBR3 tumor cells transduced with lentivirus vector expressing mCherry-CDC37, are shown by western blotting. (n = 3 independent experiments). (**L**) The internalized mCherry-CDC37 in DCs was analyzed by flow cytometry. Representative flow cytometric plots and quantification of the percentages of mCherry-CDC37 signals in DCs.

(**M**). GFP-RAB5-overexpressing DCs were treated with tumor EVs of mCherry-CDC37 overexpressing SKBR3. Representative immunofluorescence images of CDC37 and RAB5 costaining in DCs. Scale bar, 5 mm.

(**N**). Representative live cell imaging for GFP-RAB5-overexpressing DCs treated with EVs from mCherry-CDC37 overexpressing SKBR3 cancer cells at indicated time points (n = 3 independent experiments). Scale bar, 5μm.

(**O**). Quantification of MFI of mCherry-CDC37 EV uptake by DCs after pretreatment with indicated endocytic pathway inhibitors.

(**P,Q**)**.**Single-cell suspensions were isolated from EO771 tumor grafts and incubated with mCherry-CDC37 tumor EVs. Flow cytometry was used to quantify the internalization of mCherry-CDC37 in intratumoral DCs, macrophages, fibroblasts, B cells, and T cells. (**P**) Gating strategies for DCs, macrophages, fibroblasts, B cells, and T cells in EO771 tumor grafts. (**Q**) Representative flow cytometric histogram and quantification of MFI of mCherry-CDC37 EV uptake by intratumoral cell populations.

(**R**)**.** Representative flow cytometric plots and quantification of the percentages of GZMB^+^CD8^+^ T cells primed by DC or macrophages treated with EVs from wild type or CDC37^KO^ MDA-MB-468 breast cell line.

Results are mean ± s.d. of independent experiments producing similar results. ***P* < 0.01 compared with TBMhiCTLhi TiDCs were determined by two-tailed student’s t test (**A,R**). ***P* < 0.01, ****P* < 0.001, compared with DCs transduced with indicated sgRNA and treated with PBS (**D-F**), or CK^+^ cells (**J**), untreated SKBR3 cells (**L**), DMSO control (**O**) or DCs (**Q**) were determined by two-tailed one-way ANOVA with Dunnett’s multiple-comparisons test (**D-F, J, L, O, Q**). ***P<0.001, compared to tumor epithelial cells (**H**), determined by Kruskal–Wallis H test, followed by pairwise two-tailed Wilcoxon rank-sum tests with Benjamini–Hochberg (BH) correction for multiple comparisons (**H**).


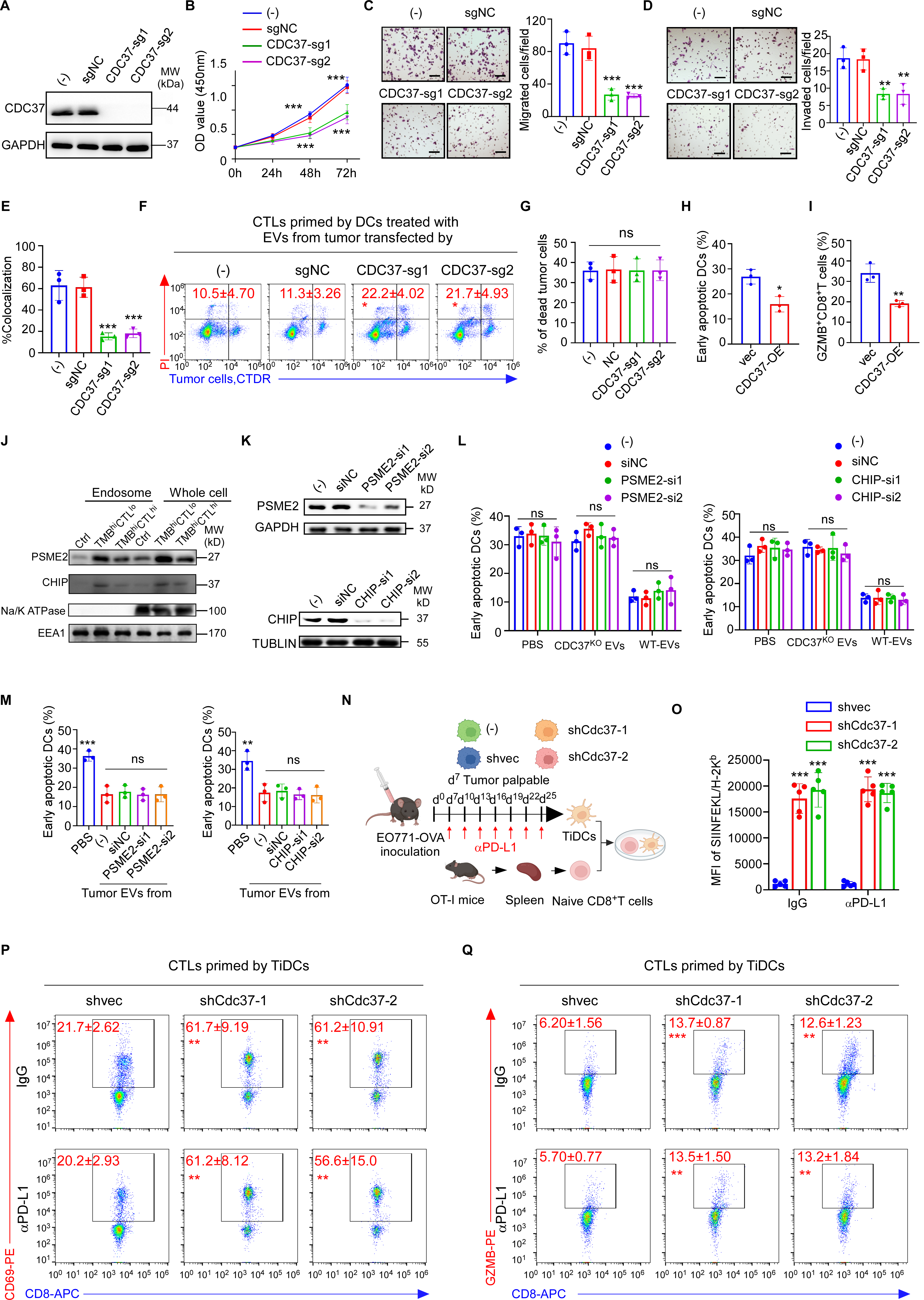


**Figure S4: Silencing CDC37 in tumor cells enhanced antigen cross-presentation by DCs in tumors more than directly affecting the tumor cells themselves.**

(**A-D**). MDA-MB-468 breast cancer cells were transduced with sgNC, CDC37-sg1 or CDC37-sg2. (**A**) Representative images of CDC37 expression in MDA-MB-468 breast cancer cells with indicated treatment, are shown by western blotting (n = 3 independent experiments). (**B**) Quantification of optical density (OD) of indicated MDA-MB-468 tumor cells subjected to CCK-8 assay after transduction (n=3 independent experiments). (**C,D**) Representative images and quantification of transwell migration (**C**) and invasion (**D**) assay of MDA-MB-468 after transduction (n=3 independent experiments). Scale bars, 100 µm.

(**E**). Quantification of the area ratio of CDC37 colocalized with RAB5 relative to the total CDC37 in DCs treated with indicated tumor EVs (n = 3 independent experiments).

(**F**). DCs were treated with indicated tumor EVs and co-cultured with autologous naive CD8^+^ T cells, respectively. Tumor cell death induced by *in vitro* primed CTLs was examined by PI uptake through flow cytometry and quantified. Numbers in the plots indicate percentages of PI^+^ tumor cells (n = 3).

(**G**) MDA-MB-468 were transduced with sgNC, sCDC37-sg1 or CDC37-sg2, respectively, and co-cultured with tumor-specific CD8⁺ T cells pre-activated by normal DCs loaded with tumor lysates. Tumor cell death induced by tumor-speicific CTLs was examined by PI uptake through flow cytometry and quantified. Numbers in the plots indicate percentages of PI+ tumor cells.

**(H-I)**. DCs were treated with tumor EVs from SKBR3 tumor cells transduced with control vector or CDC37-overexpression (CDC37-OE) lentivirus, respectively. **(H)** Quantification of early cell apoptosis induced by endosome-cytosol export of internalized cytC in DCs treated with indicated tumor-EVs. **(I)** Quantification of GZMB expression in the CD8^+^T cells primed by DCs treated with indicated tumor-EVs.

(**J**)**.** Representative images of PSME2 and CHIP expression in the endosomes and whole cell of DCs with indicated treatment, are shown by western blotting (n = 3 independent experiments). Na/K ATPase was used as a marker of cell membrane protein and EEA1 was used as a marker of endosomes. MW, molecular weight.

(**K,L**). DCs were transduced with negative control siRNA (siNC), siRNAs targeting PSME2 (PSME2-si1, PSME2-si2) or CHIP (CHIP-si1, CHIP-si2), and treated with PBS, EVs from wild-type or CDC37^KO^ MDA-MB-468, respectively. (**K**) Representative images of the PSME2 or CHIP expression in DCs, determined by western blotting. (**L**) Quantification of AnnexinV^+^PI^-^ staining in PSME2^KD^ or CHIP^KD^ DCs with indicated treatment and then treated with exogenous cytC..

(**M**). DCs were treated with PBS, tumor EVs from wild type MDA-MB-468 breast cancer cells (-) or MDA-MB-468 tumor cells transduced with siNC, PSME2-si1, PSME2-si2, CHIP-si1 or CHIP-si2, respectively. Quantification of AnnexinV^+^PI^-^ staining in DCs treated with indicated tumor EVs and then treated with exogenous cytC.

(**N-O**). EO771-OVA mouse breast cancer cells transduced with shvec, shCdc37-1 or shCdc37-2 were inoculated to the mammary fat pads of immunocompetent syngeneic C57BL/6 mice, and intraperitoneally administered the mice with anti-PD-L1 immunotherapy or IgG every three days initiated on the seventh day following tumor inoculation (n = 5 per group).

(**N**). Experimental schematic.

(**O**). Quantification of mean fluoresent intensity (MFI) of SIINFEKL-H-2K^b^ complex on the TiDCs isolated from indicated mouse model assessed by flow cytometry (n = 5 per group).

(**P,Q**). CD69 (**P**) and GZMB (**Q**) expression on OT-I CD8^+^ T cells primed by TiDCs isolated from indicated mouse model assessed by flow cytometry (n = 5 per group).

Results are mean ± s.d. of independent experiments producing similar results. ns, not significant, ***P* < 0.01, ****P* < 0.001, compared with wild type MDA-MB-468 (-) (**B-D, G**), DCs treated with untransfected tumor-EVs (-) (**E,F,M**), untransfected DCs (-) in corresponding group (**L**), TiDCs from shvec-transduced tumor with indicated treatments (**O,P,Q**), were determined by two-tailed one-way ANOVA with Dunnett’s multiple-comparisons test (**B-G, L,M, O-Q**). ns, not significant, were determined by two-tailed t Student’s t test **(H,I).**


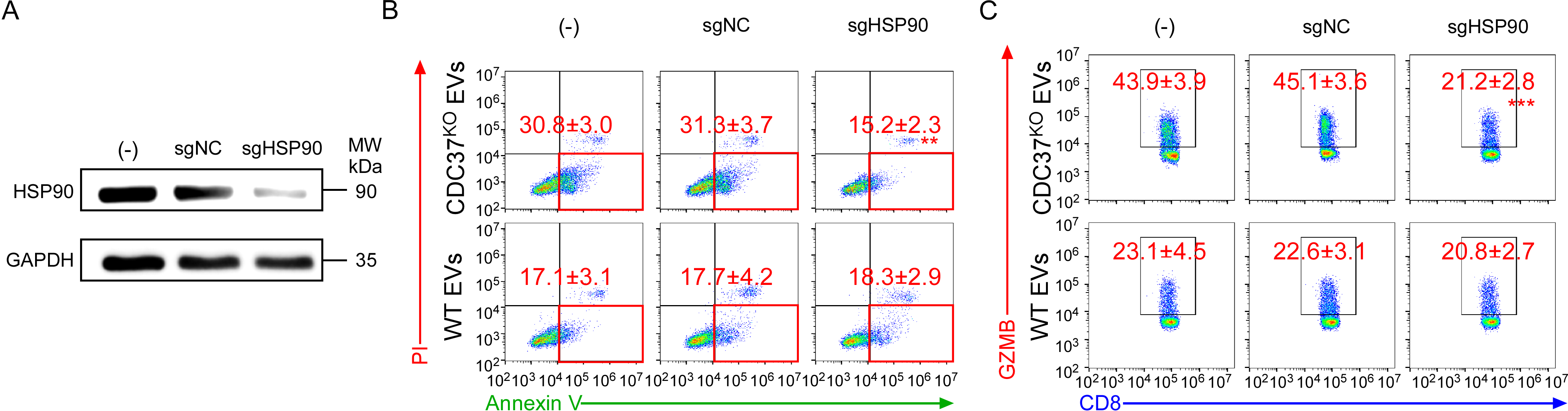


**Figure S5:** Silencing of HSP90 in DCs impairs antigen cross-presentation

(**A-C**)**.** DCs were transduced with empty sgRNA (sgNC), sgRNAs targeting HSP90 (sgHSP90), pulsed with tumor lysates, treated with EVs from wild-type or CDC37^KO^ MDA-MB-468, respectively, and co-cultured with autologous naive CD8^+^ T cells. (**A**) Representative images of HSP90 expression in DCs, are shown by western blotting (n = 3 independent experiments). (**B**) Representative flow cytometric plots and quantification of AnnexinV^+^PI^-^ DCs after exogenous cytochrome c (cytC) treatment. (**C**) Representative flow cytometric plots and quantification of the percentages of GZMB^+^CD8^+^T cells primed by indicated DCs.

Results are mean ± s.d. of independent experiments producing similar results. ns, not significant, **P* < 0.05, ***P* < 0.01, ****P* < 0.001，compared with wild-type DC (-) (**B,C**)were determined by two-tailed one-way ANOVA with Dunnett’s multiple-comparisons test (**B,C**).


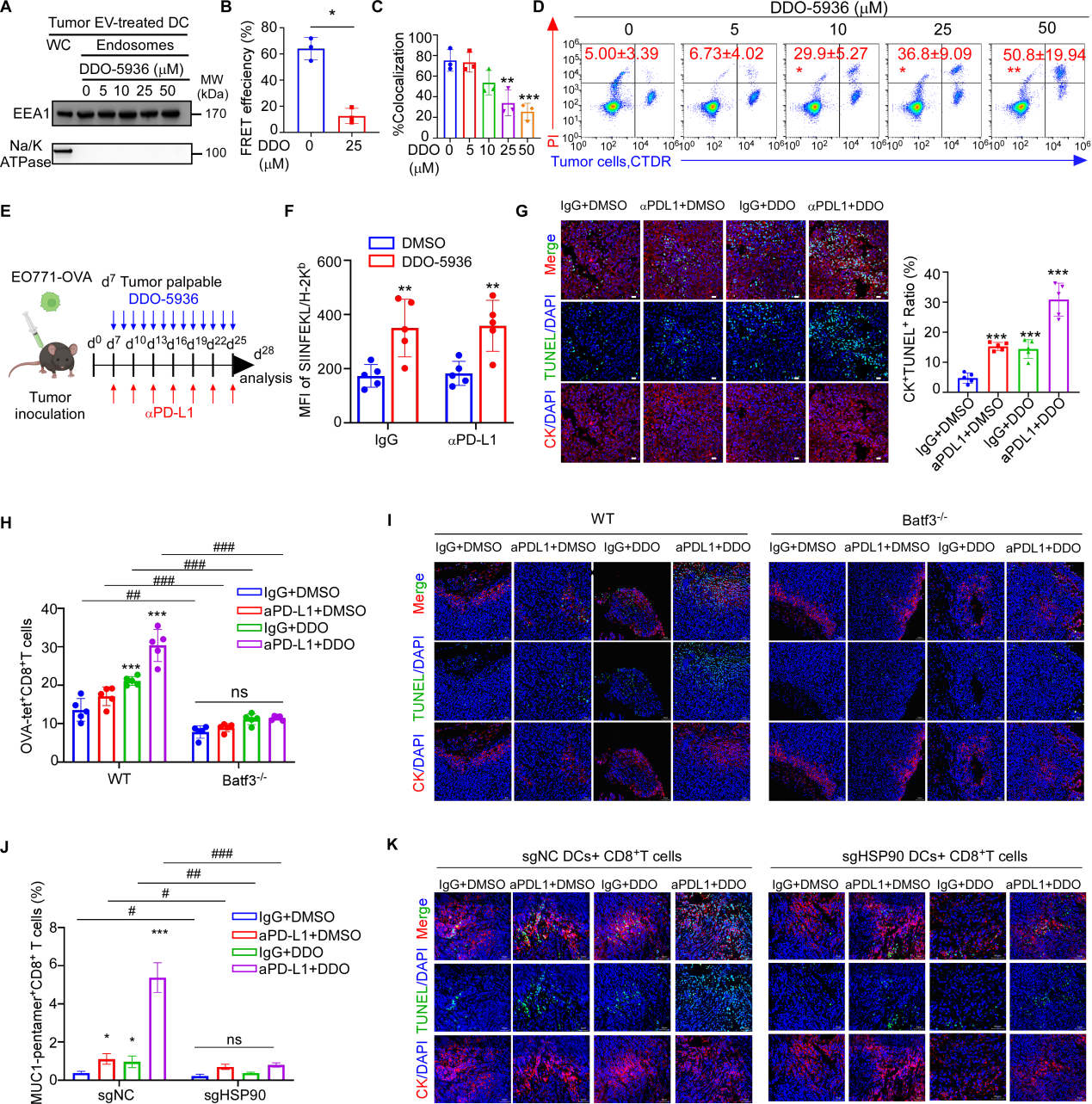


**Figure S6: Blocking the binding of CDC37 and HSP90 by small molecule inhibitor DDO-5936 promote the efficacy of ICB.**

(**A**). Western blot validation of whole cell (WC) or endosomal proteins extracted from DCs treated with EVs from MDA-MB-468, followed by DDO-5936 treatment at the indicated concentrations. (n = 3 independent experiments).

(**B**). Quantification of the percentages of FRET efficiency (FRET_eff_) signals relative to the total signal arising from the binding of CDC37-CFP to HSP90-YFP in DCs treated with CDC37-CFP-overexpressing SKBR3 tumor EVs and administrated with 25µM DDO-5936 (n = 3 independent experiments).

(**C**). Quantification of the area ratio of OVA colocalized with HSP90 relative to the total OVA in DCs pre-treated with MDA-MB-468 tumor EVs and DDO-5936 at indicated concentrations (n = 3 independent experiments).

(**D**). DCs were treated with MDA-MB-468 tumor EVs and administrated with DDO-5936 at indicated concentrations, and then co-cultured with autologous naive CD8^+^ T cells, respectively. Tumor cell death induced by *in vitro* primed CTLs was examined by PI uptake through flow cytometry and quantified. Numbers in the plots indicate percentages of PI^+^ tumor cells (n=3).

(**E-G**). EO771-OVA mouse breast cancer cells were inoculated to the mammary fat pads of immunocompetent syngeneic C57BL/6 mice, and intraperitoneally administered the mice with anti-PD-L1 immunotherapy or IgG every three days, along with daily DDO-5936 treatment, initiated on the seventh day following tumor inoculation (n = 5 per group).

(**E**). Experimental schematic.

(**F**). Quantification of MFI of SIINFEKL-H-2K^b^ complex on the TiDCs isolated from indicated mouse model assessed by flow cytometry (n = 5 per group).

(**G**). Representative immunofluorescence images and quantification of dead tumor cells denoted by co-staining with CK and TUNEL in the harvested grafts (n=5 per group). Scale bars, 50 µm.

(**H-I**). EO771-OVA mouse breast cancer cells were inoculated to the mammary fat pads of immunocompetent syngeneic C57BL/6 mice or Batf3-/- transgenic mice, and intraperitoneally administered the mice with anti-PD-L1 immunotherapy or IgG every three days along with daily DDO-5936 treatment, initiated on the seventh day following tumor inoculation (n = 5 per group). (**H**) Quantification of tumor-specific OVA-tetramer⁺ CD8⁺ T cells. Related to Figure. 6L.(**I**) Representative immunofluorescence images of dead tumor cells denoted by co-staining with CK and TUNEL in the harvested grafts. Scale bar, 50 µm. Related to Figure. 6M.

(**J-K**)**.** NSG mice bearing PDXs from TMB^hi^CTL^lo^ patient with high CDC37 tumors were transfused with autologous tumor lysate-pulsed DCs transduced with sgNC or sgHSP90 together with CD8^+^ T cells, followed by treatment with DDO-5936, anti–PD-L1, or the combination. **(J)** Quantification of tumor-specific MUC1-pentamer⁺ CD8⁺ T cells. Related to Figure. 6N. (**K**) Representative immunofluorescence images of dead tumor cells denoted by co-staining with CK and TUNEL in the harvested grafts. (n=3 per group). Related to Figure. 6O.

Results are mean ± s.d. of independent experiments producing similar results. **P* < 0.05, ***P* < 0.01, were determined by two-tailed Student’s t test (**A,F**). ns, not significant, **P* < 0.05, ***P* < 0.01, ****P* < 0.001, compared with DCs treated with DMSO (**B,D**), or untreated DC (T=0) (**C**), mice receiving IgG+DMSO (**G-K**), were determined by two-tailed one-way ANOVA with Dunnett’s multiple-comparisons test (**B-D, G-K**). ^#^*P* < 0.05, ^##^*P* < 0.01, ^###^*P* < 0.001, compared with WT mice (**H**), or mice receiving sgNC-DC (**J**), were determined by two-tailed one-way ANOVA with Tukey’s multiple-comparisons test.


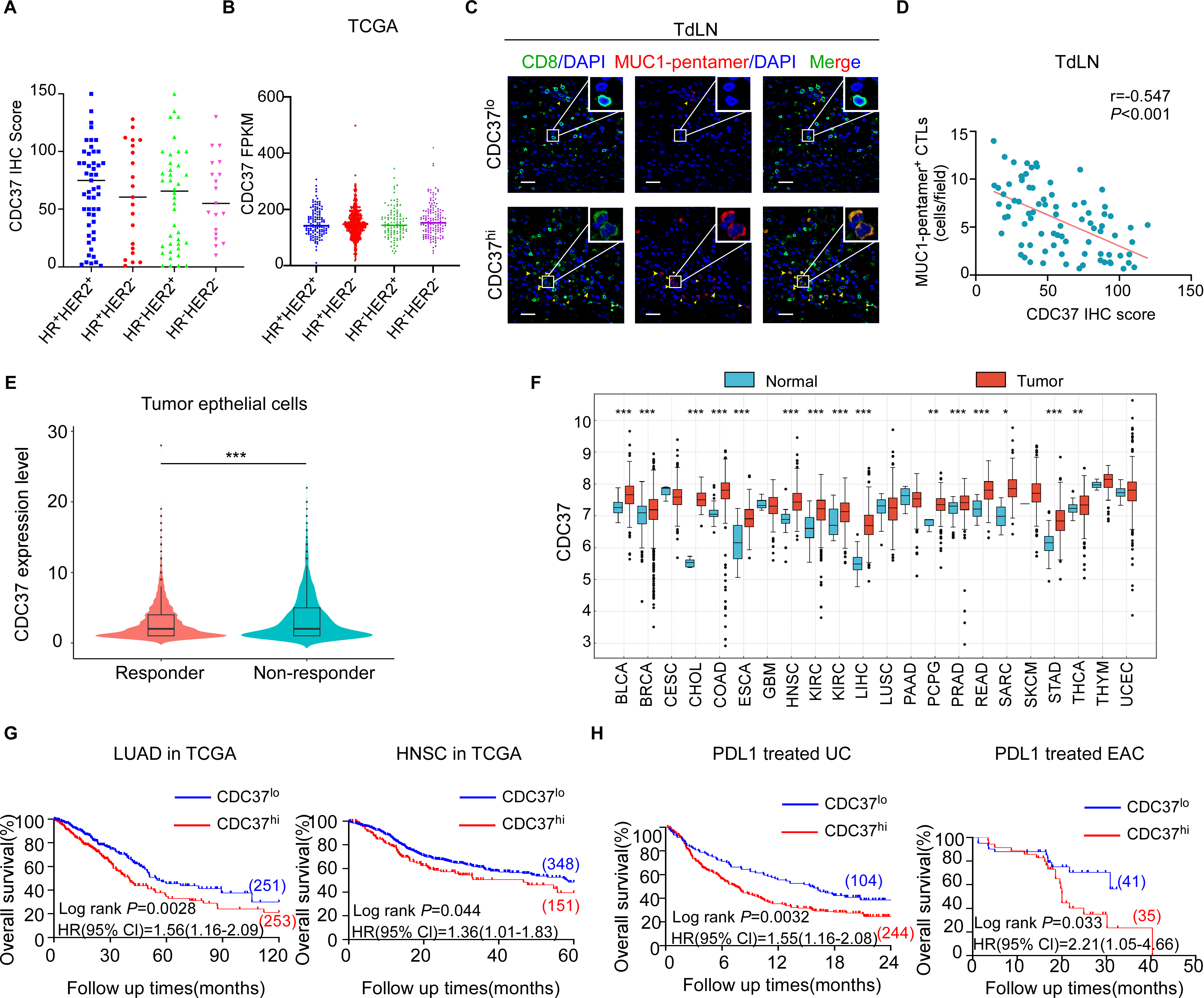


**Figure S7: CDC37 is associated with tumor-specific CTL in TdLNs, with no differences between breast cancer subtypes.**

(**A**). The CDC37 IHC scores in the tumor site from patients with different subtypes of breast cancer (mean ± s.d). HR^+^HER2^+^, n = 55; HR^+^HER2^-^, n = 21; HR^-^HER2^+^, n = 42; HR^-^HER2^-^, n = 19.

(**B**). The fragments per kilobase of exon model per million mapped fragments (FPKM) of CDC37 in the breast cancer patients from TCGA dataset with different subtypes (mean ± s.d). HR^+^HER2^+^, n = 143; HR^+^HER2^-^, n = 552; HR^-^HER2^+^, n = 40; HR^-^HER2^-^, n = 143.

(**C**). Representative images of the immunofluorescence staining for MUC1-pentamer^+^CD8^+^T cells in TdLNs from TNBC patients (n=84). Boxes denote the area of higher magnification images shown at the top right corner. Scale bar, 50 μm.

(**D**). Correlation between CDC37 IHC scores and the count of MUC1-pentamer^+^CD8^+^T cells in TdLNs of breast cancer patients, respectively (n = 84, Pearson’s correlation coefficient r and two-tailed *P* value are shown).

(**E**). Violin plots showing CDC37 expression in tumor epithelial cells from TNBC patients classified as responders (n = 6) or non-responders (n = 6) to anti-PD-1 therapy based on scRNA-seq data were obtained from publicly available sources from A. Bassez et al., 2021, Nat. Med.^[65]^

(**F**). Pan-cancer analysis, including Bladder Urothelial Carcinoma (BLCA), Breast Invasive Carcinoma (BRCA), Cervical Squamous Cell Carcinoma and Endocervical Adenocarcinoma (CESC), Cholangiocarcinoma (CHOL), Colon Adenocarcinoma (COAD), Esophageal Carcinoma (ESCA), Glioblastoma Multiforme (GBM), Head and Neck Squamous Cell Carcinoma (HNSC), Kidney Renal Clear Cell Carcinoma (KIRC), Kidney Renal Papillary Cell Carcinoma (KIRP), Liver Hepatocellular Carcinoma (LIHC), Lung Squamous Cell Carcinoma (LUSC), Pancreatic Adenocarcinoma (PAAD), Pheochromocytoma and Paraganglioma (PCPG), Prostate Adenocarcinoma (PRAD), Rectum Adenocarcinoma (READ), Sarcoma (SARC), Skin Cutaneous Melanoma (SKCM), Stomach Adenocarcinoma (STAD), Thyroid Carcinoma (THCA), Thymoma (THYM), and Uterine Corpus Endometrial Carcinoma (UCEC), showing CDC37 expression in various cancer types and their corresponding normal tissues based on TCGA datasets.

(**G**). Kaplan–Meier curves for overall survival in lung adenocarcinoma (LUAD) and head and neck squamous cell carcinoma (HNSC) cancer patients based on TCGA datasets with high (CDC37^hi^) and low (CDC37^lo^) CDC37 expression. Sample sizes for each group are indicated in the figure. Log-rank *P*, hazard ratio (HR), and 95% confidence interval (95% CI) are shown.

(**H**). Kaplan–Meier curves for overall survival in urothelial carcinoma (UC) and esophageal adenocarcinoma (ECA) patients receiving anti-PD-L1 therapy based on TCGA datasets, stratified by high (CDC37^hi^) and low (CDC37^lo^) CDC37 expression. Sample sizes for each group are indicated in the figure. Log-rank *P*, hazard ratio (HR), and 95% confidence interval (95% CI) are shown.

Results are mean ± s.d. of independent experiments producing similar results. ****P*<0.001, determined by the two-sided Wilcoxon rank-sum test (**E**). **P* < 0.05, ***P* < 0.01, ****P* < 0.001, *****P* < 0.0001 compared with corresponding normal tissues, were determined by Man-Whitney U test. (**F**).

**Table S1. Predictive values of tumor-specific CTLs and TMB for ICB responsiveness**

|  |  | **All patients (n=84)** | | | **TMB^hi^ patients (n=41)** | | |
| --- | --- | --- | --- | --- | --- | --- | --- |
|  |  | **OR** | **95%CI** | ***P* value^c^** | **OR** | **95%CI** | ***P* value^c^** |
| **GZMB^+^CTL (cells/field)** | Univariate | 1.620 | 1.297-2.110 | <0.001^***^ | 1.622 | 1.175-2.430 | 0.0078^**^ |
|  | Multivariate^a^ | 1.785 | 1.357-2.523 | 0.002^**^ | 1.982 | 1.215-3.969 | 0.0187^*^ |
| **MUC1-pent^+^ CTL**  **(cells/field)** | Univariate | 2.637 | 1.847-4.157 | <0.001^***^ | 2.513 | 1.553-4.830 | 0.0011^**^ |
|  | Multivariate^a^ | 1.845 | 1.384-2.618 | <0.001^***^ | 4.008 | 1.797-14.99 | 0.0073^**^ |
| **Dichotomized TMB (Median)** | Univariate | 2.721 | 1.131-6.782 | 0.0278^*^ | - | - | - |
|  | Multivariate^b^ | 3.417 | 1.09411.88 | 0.0407^*^ | - | - | - |

Abbreviation: CI, confidence interval; OR, odds ratio; TMB, tumor mutational burden; GZMB, granzyme B; MUC1-pent, MUC1-pentamer, CTL, cytotoxic T lymphocytes.

^a^ Including age, tumor size, lymphatic metastasis, stage, TMB, PD-L1 status.

^b^ Including age, tumor size, lymphatic metastasis, stage, GZMB^+^CTL, PD-L1 status.

^c^Univariate and multivariate logistic regression models adjusted for prespecified variables were used. All *P*-values are two-sided and *P* ≤ 0.05 was considered as significant. ^*^*P*<0.05, ^**^*P* < 0.01, ^***^*P* < 0.001, statistically significant.

**Table S3. Correlation of CDC37 expression in tumor slices with clinical characteristics in 138 cases of triple negative breast cancer patients**

| **Characteristics** | **No. of patient** | **CDC37 expression^a^** | | ***P* value^b^** | |
| --- | --- | --- | --- | --- | --- |
|  |  | **Low(≤ 51)** | **High (> 51)** |  |  |
| **Age** |  | 69 | 69 | |  |
| ≤50 | 75 | 34 (45.33%) | 41 (54.67%) | | 0.232 |
| >50 | 63 | 35 (55.56%) | 28 (44.44%) | |  |
| **Tumor size** | 138 |  |  | |  |
| T1-T2 | 117 | 64 (54.70%) | 53 (45.30%) | | 0.009^**^ |
| T3-T4 | 21 | 5 (23.81%) | 16 (76.19%) | |  |
| **Lymph node status** |  |  |  | |  |
| N0-N1 | 117 | 60 (51.28%) | 57 (48.72%) | | 0.477 |
| N2-N3 | 21 | 9 (42.86%) | 12 (57.14%) | |  |
| **Metastasis** |  |  |  | |  |
| M0 | 132 | 67 (50.76%) | 65(49.24%) | | 0.404 |
| M1 | 6 | 2 (33.33%) | 4 (66.67%) | |  |
| **TNM stage** |  |  |  | |  |
| I-II | 111 | 59 (53.15%) | 52 (46.85%) | | 0.133 |
| III-IV | 27 | 10 (37.04%) | 17 (62.96%) | |  |
| **Histological grade** |  |  |  | |  |
| DCIS-II | 15 | 8 (53.33%) | 7 (46.67%) | | 0.784 |
| III | 123 | 61 (49.59%) | 62 (50.41%) | |  |

Abbreviation: T, tumor stage; N, nodal stage; M, distant metastasis;

^a^CDC37 expression in the breast cancer tissues, defined as IHC score, was evaluated by immunohistochemical staining (IHC). The cutoff point of CDC37 expression was demarcated according to the median value of CDC37 IHC score.

^b^All *P* values were calculated with two-sided Chi-square test. ^**^*P* < 0.01, statistically significant.

**Table S4. Cox regression analysis overall survival (OS), according to CDC37 expression and clinicopathologic factors, in 138 cases of triple negative breast cancer patients.**

| **Characteristics** | **Univariate analysis** | | | **Multivariate analysis^b^** | | |
| --- | --- | --- | --- | --- | --- | --- |
|  | **HR** | **95%CI** | ***P* value** | **HR** | **95%CI** | ***P* value** |
| **CDC37 expression^a^**  (High vs low) | 2.240 | 1.376-5.096 | 0.004^**^ | 2.167 | 1.068-4.399 | 0.032^*^ |
| **Tumor size**  $($T1-2 vs T3-4) | 7.618 | 4.035-14.383 | <0.001^***^ | 4.069 | 1.731-9.561 | <0.001^***^ |
| **Lymph node status**  (N0-N1 vs N2-N3) | 6.048 | 3.276-11.372 | <0.001^***^ | 3.418 | 0.840-13.898 | 0.086 |
| **Metastasis**  (M0 vs M1) | 8.878 | 3.2001-11.429 | <0.001^***^ | 1.126 | 0.345-3.673 | 0.844 |
| **TNM stage**  (I-II vs III-IV) | 5.684 | 3.067-10.5334 | <0.001^***^ | 1.390 | 0.356-5.435 | 0.636 |
| **Histological grade**  (DCIS-II vs III) | 2.824 | 0.682-11.687 | 0.152 | - | - | - |
| **Age**  (≤50 vs >50) | 0.507 | 0.267-0.964 | 0.038^*^ | 0.323 | 0.156-0.672 | 0.002^**^ |

Abbreviation: HR: hazard ratio, CI: confidence interval; T, tumor stage; N, nodal stage; M, distant metastasis; DCIS, ductal carcinoma in situ.

^a^CDC37 expression was demarcated according to the median value of CDC37 IHC score, evaluated by IHC staining, and considered as categorical variables.

^b^Multivariable Cox regression analysis was performed using stepwise selection (Forward: LR) based on the partial likelihood ratio.

All *P* values determined by univariate and multivariate Cox regression analyses were conducted with two-sided tests. ^*^*P*<0.05, ^**^*P* < 0.01, ^***^*P* < 0.001, statistically significant.

**Table S5. Key resource table**

| **REAGENT or RESOURCE** | | | **SOURCE** | **IDENTIFIER** | |
| --- | --- | --- | --- | --- | --- |
| **Antibodies** | | | | | |
| Mouse anti-human CDC37 | | | Proteintech | Cat# 66420-1-Ig | |
| Anti-human HLA-A*02-restricted MUC1-Pentamer-PE | | | Proimmune | Cat# 208-2A-G | |
| Rabiit anti-human Granzyme B | | | Abcam | Cat# 22645-206 | |
| Mouse anti-human CD8 alpha | | | Abcam | Cat# 17147 | |
| Goat Anti-Mouse IgG H&L(HRP) | | | Abcam | Cat# 6789 | |
| Rabbit anti-human CD11c | | | Abcam | Cat# 52638 | |
| Mouse anti-human CD1a | | | Abcam | Cat# 201337 | |
| Mouse anti-human CD141 | | | Abcam | Cat# 6980 | |
| Rabbit anti-human RAB5 | | | Abcam | Cat# 21801 | |
| Rabbit anti-human HSP90 | | | CST | Cat# 4877 | |
| Rabbit anti-human HSP90 | | | Proteintech | Cat#13171-1-AP | |
| Rabbit anti human/mouse wide spectrum Cytokeratin | | | Abcam | Cat# 9377 | |
| Rabbit anti human α-Smooth Muscle Actin | | | CST | Cat# 19245 | |
| Mouse anti-human CD68 | | | Abcam | Cat# 955 | |
| Rabbit anti-human CDC37 | | | Proteintech | Cat# 10218 | |
| Rabbit anti-His | | | CST | Cat# 9367 | |
| Mouse anti-human MUC-1 | | | Immunoway | Cat# 4532 | |
| Rabbit anti-humuan Alix | | | Proteintech | Cat# 12422 | |
| Mouse anti-human CD81 | | | Proteintech | Cat# 66866 | |
| Rabbit anti-human CD63 | | | Abcam | Cat# 5702 | |
| Rabbit anti-human EEA1 | | | Abcam | Cat# 4245 | |
| Rabbit anti-human Sodium Potassium ATPase | | | Abcam | Cat# 1845Y | |
| Rabbit anti-human PSME2 | | | Abcam | Cat# ab183727 | |
| Mouse anti-human CHIP | | | Santa Cruz | Cat# sc-133066 | |
| Mouse anti-human GAPDH | | | Proteintech | Cat# 60004 | |
| HRP-linked anti-rabbit IgG | | | CST | Cat# 7074 | |
| HRP-linked anti-mouse IgG | | | CST | Cat# 7076 | |
| Live/Dead Fixable Viability Dye | | | eBioscience | Cat# 65-0865-18 | |
| Donkey anti-Mouse IgG (H+L), Alexa Fluor™ 488 | | | Thermo Fisher | Cat# A21202 | |
| Donkey anti-Mouse IgG (H+L), Alexa Fluor™ 647 | | | Thermo Fisher | Cat# A31571 | |
| Donkey anti-Rabbit IgG (H+L), Alexa Fluor™ 555 | | | Thermo Fisher | Cat# A31572 | |
| Donkey anti-Rabbit IgG (H+L), Alexa Fluor™ 488 | | | Thermo Fisher | Cat# A21206 | |
| Donkey anti-Rabbit IgG (H+L), Alexa Fluor™ 647 | | | Thermo Fisher | Cat# A32795 | |
| Donkey anti-Mouse IgG (H+L), Alexa Fluor™ 555 | | | Thermo Fisher | Cat# A31570 | |
| Goat anti-Rabbit IgG (H+L), Alexa Fluor™ 750 | | | Thermo Fisher | Cat# A21039 | |
| Mouse anti-human CD8-APC | | | BioLegend | Cat# 980904 | |
| Mouse anti-human CD69-Brilliant Violet 421™ | | | BioLegend | Cat# 310930 | |
| Mouse anti-human/mouse Granzyme B-Pacific Blue^TM^ | | | BioLegend | Cat# 372218 | |
| Rat anti-human IFN-γ-PE | | | BioLegend | Cat# 383304 | |
| Mouse anti-human CD11c -FITC | | | BioLegend | Cat# 337214 | |
| Mouse anti-human CD141-APC | | | BioLegend | Cat# 344106 | |
| Mouse anti-human CD1a-APC | | | BioLegend | Cat# 985604 | |
| Mouse anti-human HLA-DR-PE | | | BioLegend | Cat# 327008 | |
| Mouse anti-human CD80-APC | | | BioLegend | Cat# 305220 | |
| Mouse anti-human CD86- Pacific Blue^TM^ | | | BioLegend | Cat# 305423 | |
| Mouse anti-mouse H-2K^b^ bound to SIINFEKL-APC | | | BioLegend | Cat# 141606 | |
| T-Select H-2Kb OVA Tetramer-SIINFEKL-PE | | | MBL | Cat# TS-5001-1C | |
| Rat anti-mouse CD8a-APC | | | BioLegend | Cat# 162306 | |
| Rat anti-mouse IFN--PE | | | BioLegend | Cat# 505808 | |
| Hamster anti-mouse CD11c-FITC | | | BioLegend | Cat# 117306 | |
| Rat anti-mouse CD45- Brilliant Violet 421™ | | | BioLegend | Cat# 147719 | |
| Rat anti-mouse CD103-PE | | | BioLegend | Cat# 110904 | |
| Rat anti-mouse CD19-APC | | | BioLegend | Cat# 152409 | |
| Armenian Hamster anti-mouse CD3- Pacific Blue | | | BioLegend | Cat# 100333 | |
| Rat anti-mouse I-A/I-E-FITC | | | BioLegend | Cat# 107605 | |
| Rat anti-mouse CD11c- Brilliant Violet 421™ | | | BioLegend | Cat# 117329 | |
| Rat anti-mouse CD45- Brilliant Violet 650™ | | | BioLegend | Cat# 103151 | |
| Rat anti-mouse F4/80-FITC | | | BioLegend | Cat# 123107 | |
| Rat anti-mouse CD11b-APC | | | BioLegend | Cat# 101205 | |
| Mouse anti-human/mouse/rat αSMA | | | R＆D | Cat# MAB1420-SP | |
| Purified Rat Anti-Mouse CD16/CD32 antibody | | | BD Biosciences | Cat# 553141 | |
| Propidium Iodide | | | Thermo Fisher | Cat# 00-6990-50 | |
| **Chemicals, Peptides, and Recombinant Proteins** | | | | | |
| Polybrene | | | Sigma | Cat# TR-1003 | |
| Lipofectamine 3000 | | | Invitrogen | Cat# L3000075 | |
| Ficoll | | | TBDscience | Cat#LTS1007 | |
| Percoll | | | GE Healthcare | Cat# 17-0891-01 | |
| Anti-mouse PD-L1(B7-H1) | | | BioXcell | Cat# BE0101 | |
| InVivoMAb rat IgG2b isotype control | | | BioXcell | Cat# BE0090 | |
| DDO-5936 | | | MCE | Cat# 139301 | |
| 4% paraformaldehyde | | | Thermo Fisher | Cat# FB002 | |
| DAB | | | Gene Tech | Cat# GK500710 | |
| Hoechst 33342 | | | Thermo Fisher | Cat# H21492 | |
| Collagenase I | | | Worthington | Cat# LS004197 | |
| Collagenase IV | | | Worthington | Cat# LS004188 | |
| DNase I | | | Roche | Cat# 10104159001 | |
| 10 3 TrypLE Selected Enzyme | | | GIBCO | Cat#A1217701 | |
| Recombinant Human IL-4 | | | Peprotech | Cat# 200-04 | |
| Recombinant Human GM-CSF | | | Peprotech | Cat# 300-03 | |
| Recombinant Mouse IL-4 | | | Peprotech | Cat# 214-14 | |
| Recombinant Mouse GM-CSF | | | Peprotech | Cat# 315-03 | |
| Ovalbumins | | | MCE | Cat# 9006-59-1 | |
| MUC1 protein | | | MCE | Cat# HY-P78740 | |
| LPS | | | Sigma-Aldrich | Cat#L2880 | |
| MG-132 | | | Sigma-Aldrich | Cat# M8699 | |
| cathepsin inhibitor leupeptin | | | Sigma-Aldrich | Cat# L2884 | |
| Lactacystin | | | Sigma-Aldrich | Cat# L6785 | |
| Eeyarestatin I | | | Sigma-Aldrich | Cat# E1286 | |
| Cytochalasin D | | | Sigma-Aldrich | Cat# C2618 | |
| Ethylisopropylamiloride | | | MCE | Cat# L593754 | |
| LY294002 | | | Sigma-Aldrich | Cat# 440202 | |
| Chlorpromazine | | | Sigma-Aldrich | Cat# 215921 | |
| Dynasore | | | Sigma-Aldrich | Cat# D7693 | |
| cytC,from equine heart | | | Sigma-Aldrich | Cat# 9007-43-6 | |
| CCF4/AM | | | Thermo Fisher | Cat# K1029 | |
| purified β-lactamase | | | Prospec | Cat# ENZ-351 | |
| Sulfate latex, 8% w/v, 3μm | | | Thermo Fisher | Cat# S37223 | |
| Sulfate latex, 8% w/v 0.1 μm | | | Thermo Fisher | Cat# S37204 | |
| Trypan blue | | | Sigma-Aldrich | Cat# T8154 | |
| Deep Red CellTtracker Dye | | | Thermo Fisher | Cat#C34565 | |
| CD8 MicroBeads,human | | | Miltenyi Biotec | Cat# 130-045-201, | |
| CD8 (TIL)MicroBeads,mouse | | | Miltenyi Biotec | Cat# 130-116-478 | |
| Monocyte Isolation kit (BM) | | | Miltenyi Biotec | Cat# 130-100-629 | |
| FcR Blocking Reagent,human | | | Miltenyi Biotec | Cat# 130-059-901 | |
| FcR Blocking Reagent,mouse | | | Miltenyi Biotec | Cat# 130-092-575 | |
| TRIzol Reagent | | | Thermo Fisher | Cat# 15596018 | |
| Dimethyl sulfoxide | | | Sigma-Aldrich | Cat#D2650 | |
| Ripa lysis buffer,10X | | | Thermo Fisher | Cat# 89900 | |
| Pierce^TM^ IP lysis buffer | | | Thermo Fisher | Cat# 87787 | |
| Pierce^TM^ Protein A/G Magnetic Beads | | | Thermo Fisher | Cat# 88802 | |
| Ponceau S solution | | | R＆D | Cat# 6226-79-5 | |
| Aqueous solution | | | Sigma-Aldrich | Cat# V5265 | |
| eBioscience™ Flow Cytometry Staining Buffer | | | Thermo Fisher | Cat# 00-4222-57 | |
| IC fixation buffer | | | Thermo Fisher | Cat# FB001 | |
| eBioscience™ permeabilization buffer | | | Thermo Fisher | Cat# 00-8333-56 | |
| Amine-modified polystyrene beads | | | Polysciences | Cat# 9003-53-6 | |
| PVDF membranes | | | Millipore | Cat# IPVH00010 | |
| **Critical Commercial Assays** | | | | | |
| LIVE/DEAD Fixable Yellow Dead Cell Stain Kit | | | Thermo Fisher | Cat# L34959 | |
| Tumor Cell Isolation Kit | | | Miltenyi Biotec | Cat# 130-108-339 | |
| eBioscience^TM^ Cell Stimulation Cocktail | | | Thermo Fisher | Cat# 00-4970-03 | |
| PrimeScriptTM RT reagent Kit | | | Takara | Cat# RR047A | |
| TB Green® Premix Ex Taq™ II FAST qPCR | | | Takara | Cat# CN830S | |
| Annexin V Apoptosis Detection Kit | | | BioLegend | Cat# 640932 | |
| Halt^TM^ Protease & PhosphataseInhibitor Cocktail (100×) | | | Thermo Fisher | Cat# 78446 | |
| SuperSignal™ West Pico PLUS Chemiluminescent Substrate | | | Thermo Fisher | Cat# 34580 | |
| TUNEL Assay Kit-FITC | | | Abcam | Cat# 66108 | |
| Pierce BCA Protein Assay Kit | | | Thermo Fisher | Cat# 23225 | |
| Cell Counting Kit-8 | | | Beyotime | Cat# C0038 | |
| **Software and Algorithms** | | | | | |
| FlowJo | | | FlowJo | https://www.flowjo.com/ | |
| GraphPad Prism | | | GraphPad Software, Inc | https://www.graphpad.com/ | |
| ZEN 2021 light Edition | | | Zeiss |  | |
| ImageJ | | | NIH | https://imagej.nih.gov/ij/ | |
| Prism 9.0 | | | GraphPad Software | https://www.graphpad.com/ | |
| SPSS v.26 | | | N/A | http://www.spss.com.cn/ | |
| **Primer sequences for qRT-PCR** | | | | | |
| **Name** | | **Forward** | | | **Reverse** |
| CDC37 | 5’-AACACAAGACCTTCGTGGAAAA-3’ | | | 5’-TAATTGGCTGTCTCCTCGCAC-3’ | |
| HSP90aa1 | 5’-AGGAGGTTGAGACGTTCGC-3’ | | | 5’-AGAGTTCGATCTTGTTTGTTCGG-3’ | |
| GADPH | 5’-GGAGCGAGATCCCTCCAAAAT-3’ | | | 5’-GGCTGTTGTCATACTTCTCATGG-3’ | |
| RAB5 | 5’-AGACCCAACGGGCCAAATAC-3’ | | | 5’-GCCCCAATGGTACTCTCTTGAA-3’ | |
| MUC1 | 5’-TGCCGCCGAAAGAACTACG-3’ | | | 5’-TGGGGTACTCGCTCATAGGAT-3’ | |
| Mouse Cdc37 | 5’-ACAGCCAGAAATACCTGTCGG-3’ | | | 5’-GGTCAGCGGTCTTGATCTTGG-3’ | |
| Mouse Gapdh | 5’-AGGTCGGTGTGAACGGATTTG-3’ | | | 5’-GGGGTCGTTGATGGCAACA-3’ | |

Tumor EV-shuttled CDC37 locks antigen/HSP90 interaction and impairs antigen cross-presentation in DCs, reducing tumor-specific CTLs and ICB efficacy in breast cancer, and targeting CDC37 restores anti-tumor immunity and reverse ICB resistance.

**Tumor-derived CDC37 inhibits antigen cross-presentation in dendritic cells and impairs anti-tumor immunity in breast cancer**


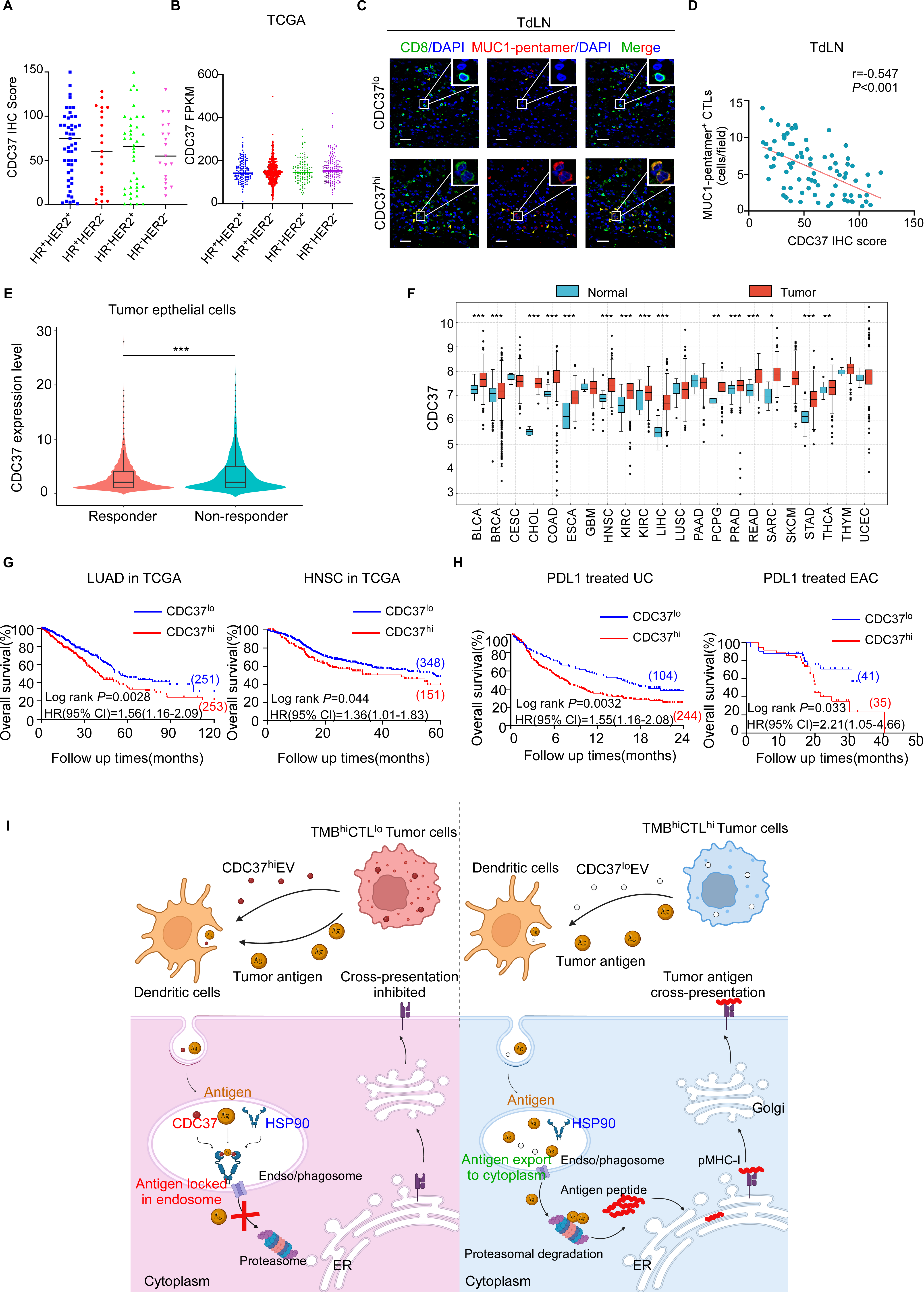

Supplement: Supplementary file 1 — Supporting Information [file ADVS-13-e06518-s003.docx]
